# Discovery of unprecedented prenylated indole piperazines and pyrazines through cryptic biosynthetic gene cluster heterologous expression

**DOI:** 10.1007/s13659-026-00601-7

**Published:** 2026-04-03

**Authors:** Ziou Zha, Dan He, Jianguo Song, Zhenhua Guan, Jiapei Han, Chang Liu, Xinyu Wang, Yongchun Zhu, Hucheng Zhu, Wencai Ye, Qin Li, Yonghui Zhang, Yuan Zhou

**Affiliations:** 1 Hubei Key Laboratory of Natural Medicinal Chemistry and Resource Evaluation, School of Pharmacy, Tongji Medical College, Huazhong University of Science and Technology, Wuhan, 430030 Hubei People’s Republic of China; 2 State Key Laboratory of Bioactive Molecules and Druggability Assessment, Jinan University, Guangzhou, 510632 Guangdong People’s Republic of China; 3 Department of Pharmacy, Shenzhen People’s Hospital (The Second Clinical Medical College, Jinan University, The First Affiliated Hospital), Southern University of Science and Technology, Shenzhen, 518055 Guangdong People’s Republic of China

**Keywords:** *Aspergillus flavipes*, Genome mining, Biosynthesis, Piperazine alkaloids, Dimethylallyl tryptophan synthases

## Abstract

Prenylation modifications of natural products typically introduce greater structural complexity and enhance their biological activities. Yet, the modification of piperazine alkaloids by dimethylallyl tryptophan synthases (DMATS) remains unreported. In this study, we identified and activated a silent DMATS-containing piperazine biosynthetic gene cluster (BGC), *flz*, in *Aspergillus flavipes* via heterologous expression and in vitro enzymatic assay, leading to the isolation and identification of sixteen metabolites. Among these, twelve are new, including five tryptophan-valine-derived alkaloids (**2**–**6**) and seven previously unreported prenylated analogs (**8**–**13**, **17**). Notably, **8** and **9** represent novel prenylated piperazines featuring a unique 6-5-5-6 ring system. Most significantly, we uncovered a versatile DMATS, FlzE, capable of catalyzing mono-prenylation on flexible substrates, such as piperazine, pyrazine, and diketopiperazine, at multiple sites in either regular or reverse manners. This study not only expands the chemical space of indole alkaloid derivatives but also provides a versatile and engineerable biocatalyst for the prenylation of both natural and synthetic products.

**Graphical Abstract:**

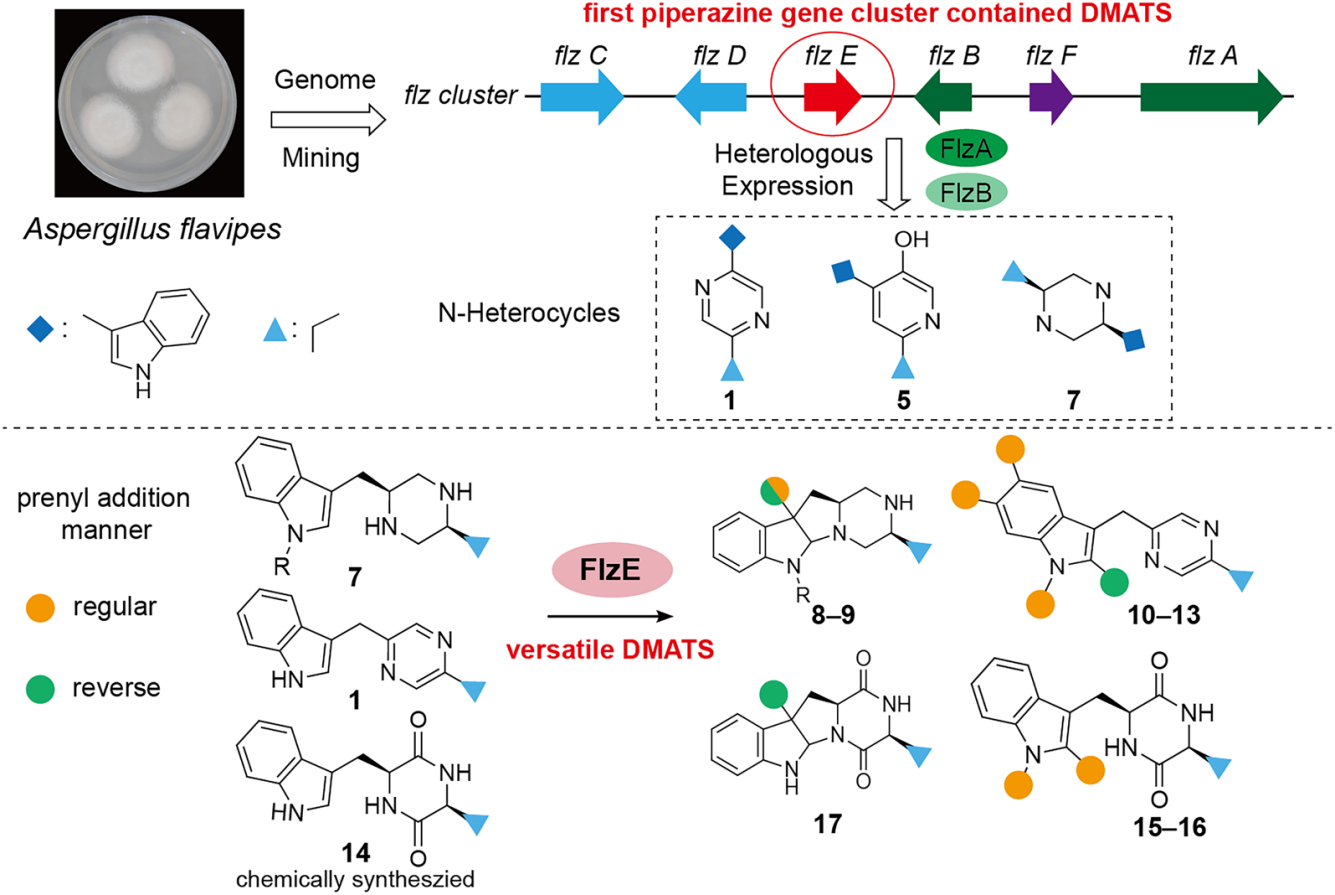

**Supplementary Information:**

The online version contains supplementary material available at 10.1007/s13659-026-00601-7.

## Introduction

**Fig. 1:**
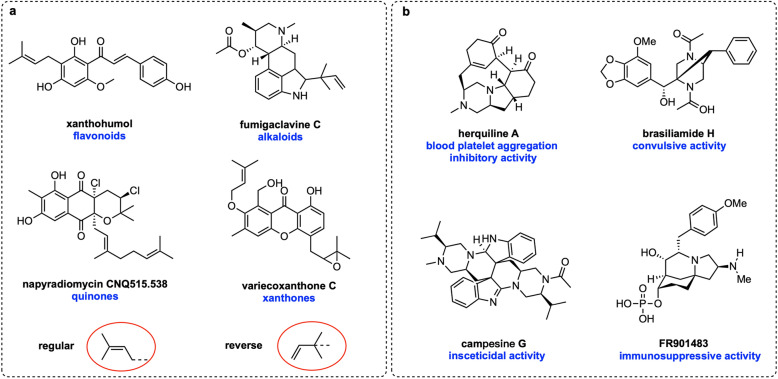
**a** The reported representative prenylated NPs, with two distinct prenylation manners highlighted in red circles. **b** Representative structures of fungal-derived piperazines and their corresponding bioactivities

Prenyltransferases (PT) are widely distributed across living organisms and are involved in essential primary and secondary metabolic pathways [1, 2]. Prenylation modifications of natural products (NPs, e.g., flavonoids, alkaloids, xanthones, and quinones) often introduces greater structural complexity into these NPs, significantly boosting their biological and pharmacological activities (Fig. 1a) [3, 4]. For instance, xanthohumol exhibits remarkable cancer chemopreventive and anti-HIV-1 activity [5, 6], while fumigaclavine C demonstrates anti-adipogenic and hepatoprotective effects [7, 8]. Dimethylallyl tryptophan synthases (DMATSs) are members of the soluble ABBA-PT superfamily, which mainly employs dimethylallyl diphosphate (DMAPP) as the prenyl donor and aromatic compounds as acceptors [9]. The prenylation reactions can occur in a regular manner (regular prenylation), where prenyl moieties are connected via their primary carbon to an acceptor, or in a reverse manner (reverse prenylation), via their tertiary carbon atoms (Fig. 1a) [10]. Notably, DMATS exhibit substantial substrate promiscuity, rendering them appealing candidates for development as versatile biocatalysts in chemoenzymatic synthesis [11].

Piperazines constitute a significant class of nitrogen-containing heterocycle compounds, finding extensive applications in pharmaceuticals, agrochemicals, dyes, preservatives, antioxidants, and surfactants [12]. A multitude of piperazine natural products have been isolated from fungi [13–15], many of which exhibit remarkable pharmacological activities (Fig. 1b). For example, herquiline A inhibits platelet aggregation [16], whereas brasiliamide H displays convulsive activity [17]. Despite the characterization of a variety of piperazine alkaloids, only eleven fungal biosynthetic gene clusters (BGCs) responsible for their production have been documented (Figure S1) [16]. Functional characterization on these BGCs reveals that construction of the piperazine core relies on two key enzymes: (1) a single module nonribosomal peptide synthetase (NRPS) with A-T-R domains (A, adenylation; T, thiolation; R, reductase); and (2) an NmrA-like reductase. The NRPS enzyme can activate two amino acids as thioesters, reduce the thioesters to the corresponding amino aldehydes, and finally generate an unstable intermediate. This intermediate is then reduced to the piperazine core by the NmrA-like reductase using two equivalents of NADPH [16]. Modifications to the piperazine skeletons are primarily introduced through oxidation, cyclization, and acylation reactions, catalyzed by CYP450s, Fe(II)/2-oxoglutarate-dependent (Fe/2OG) oxygenases, acetyltransferases, and nonribosomal peptide synthetases [16, 18–20]. However, prenylated piperazine alkaloids catalyzed by DMATSs have yet to be reported.

In this study, we identified a silent DMATS-containing piperazine BGC (designated *flz*) in *Aspergillus flavipes* by using prenyltransferase FgaPT2 as a probe. Heterologous expression of two core biosynthetic genes (*flzA* and *flzB*) and a tailoring DMATS encoding gene (*flzE*) in *Aspergillus nidulans*, combined with an in vitro chemoenzymatic assay, activated the biosynthesis of sixteen metabolites. These were subsequently isolated via mass spectrometry-guided fractionation and structurally characterized using integrated NMR, ECD, and X-ray crystallographic analyses, revealing twelve previously undescribed (**2**–**6**, **8**–**13**, and **17**) and four known (**1**, **7**, and **15**–**16**) tryptophan-valine-derived alkaloids. Among these new compounds, seven are prenylated derivatives, encompassing piperazines (**8**–**9**), pyrazines (**10**–**13**), and diketopiperazines (**17**). Among the five unprenylated new ones, three (**2**–**4**) were confirmed to be post-modified by endogenous enzymes in *A. nidulans*. Beyond the discovery of a substantial number of new compounds, our study demonstrated that FlzE is a unique DMATS capable of catalyzing mono-prenylation on flexible indole-containing substrates, such as piperazine, pyrazine, and diketopiperazine, at multiple sites in either regular or reverse manners. In summary, the activation of *flz* gene cluster and the characterization of the DMATS FlzE not only broaden the chemical diversity of prenylated alkaloids but also lay the foundation for the development of a versatile and engineerable biocatalyst for the prenylation of both natural and synthesized products that harbor the indole moiety.

## Results and discussion

### Identification of a DMATS-containing Gene Cluster Producing Pyrazines and Piperazines

**Fig. 2:**
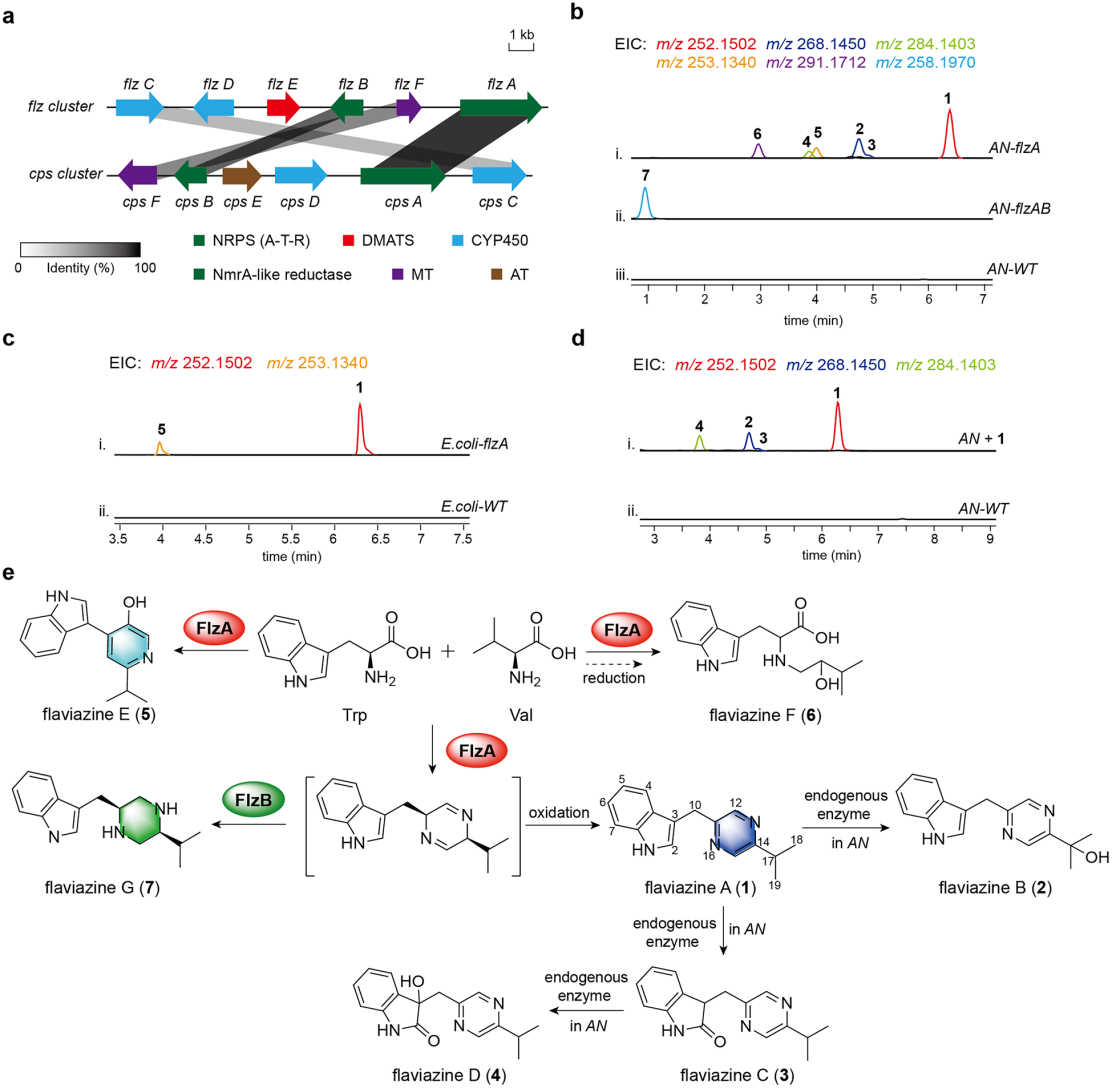
**a** Comparative representation of the *flz* gene cluster from *A. flavipes* and the *cps* gene cluster from *Aspergillus campestris*. **b** LC–MS analysis of the *A. nidulans* control and the transformants *AN-flzA* and *AN-flzAB*. EIC, extracted ion chromatography. **c** LC–MS analysis of the *E. coli* control and the transformant *E. coli-flzA.* **d** LC–MS analysis of the *A. nidulans* fed with **1** and the untreated control. **e** Proposed biosynthetic pathway of compounds **1**–**7**; compounds **2**–**6** are new

In a previous study, we sequenced the genome of an *A. flavipes* strain sourced from the intertidal zone. Bioinformatics analysis (via the antiSMASH website [21]) identified 78 secondary metabolite BGCs in its genome, [22] indicating significant potential for natural product synthesis. To explore the capability of *A. flavipes* to produce prenylated alkaloids, we targeted a silent indole derivative BGC, designated as *flz*, using the DMATS-type enzyme FgaPT2 from *Aspergillus fumigatus* as a probe (Fig. 2a and Table S3) [23]. The *flz* gene cluster contains two core biosynthetic genes: *flzA*, encoding an NRPS with A-T-R domains, and *flzB*, encoding an NmrA-like reductase. Sequence alignment revealed that *flzA* and *flzB* share 70% and 62% amino acids identity, respectively, with the two-gene cassette *cpsA* and *cpsB* in the *cps* BGC, which is solely reported to be responsible for the biosynthesis of (*S,S*)-trypyl-valyl piperazine derivatives (Fig. 2a) [18]. Unlike the core genes, several genes encoding tailoring enzyme, including two CYP450 genes (*flzC* and *flzD*) and a methyltransferase gene (*flzF*), displayed limited sequence identities (<50%) with those in *cps* cluster (Fig. 2a). Notably, the *flz* cluster contains a DMATS gene (*flzE*), which is absent from all the reported piperazine BGCs, suggesting its potential to synthesize unprecedented prenylated piperazines (Figure S1) [16].

To activate the production of the *flz* cluster, *flzA* was cloned under the *gpdA* promoter and transformed into the heterologous host *Aspergillus nidulans* LO8030 (*AN*) [24], yielding an overexpression strain *AN-flzA*. Liquid chromatography-mass spectrometry (LC–MS) analysis of the *AN-flzA* culture, compared to the *AN* wild control, revealed new ion peaks at *m/z* 252.1502 ([M + H]^+^), 268.1450 ([M + H]^+^), 284.1403 ([M + H]^+^), 253.1304 ([M + H]^+^), and 291.1712 ([M + H]^+^) (Fig. 2b, trace i). Following large-scale fermentation of the *AN-flzA* strain, six compounds were isolated and structurally characterized as flaviazine A (**1**), flaviazine B (**2**), flaviazine C (**3**), flaviazine D (**4**), flaviazine E (**5**), and flaviazine F (**6**) by NMR and single crystal X-ray diffraction analysis (Figs. 2e, S6–S51 and Tables S4–S9).

Compound **1** features a pyrazine skeleton formed through the condensation of one molecule L-Tyr aldehyde and one molecule L-Val aldehyde [18]. Compounds **2**, **3**, and **4** are oxygenated derivatives of compound **1** on the C-17, C-2, and both C-2/C-3 positions, respectively. Given that the NRPS FlzA lacks oxidation functionality towards the pyrazine substrate, we hypothesize that these products arise from modifications by endogenous oxidases in *A. nidulans*. To confirm this, **1** was fed to wild-type *A. nidulans*, and as expected, compounds **2**–**4** were significantly produced (Fig. 2d). Intriguingly, flaviazine E (**5**), featuring a pyridine skeleton derived from the same two amino acid precursors, was also identified in the *AN-flzA* strain. This indicated that the single-module NRPS, FlzA, can employ structurally distinct amino acids as building blocks to construct divergent nitrogen-containing heterocyclic skeletons. When *flzA* was expressed in *E. coli*, only **1** and **5** were detected in the recombinant strain, further confirming the above deductions (Fig. 2c). Compound **6**, a linear amino acid condensate with a C-14 hydroxyl group, was not produced in *E. coli*, implying its formation requires multiple endogenous enzymes, such as reductase and oxygenase, in *A. nidulans*.

Given that the formation of the piperazine skeleton necessitates the involvement of an NmrA-like reductase [16], we subsequently transformed the NmrA-like reductase-encoding gene,* flzB*, into the *AN* strain harboring *flzA*, yielding the *AN-flzAB* strain. As expected, LC–MS analysis of the fermentation extracts from *AN-flzAB* predominantly revealed a new ion peak at *m/z* 258.1970 ([M + H]^+^). Following large-scale fermentation and isolation, this compound was purified and subsequently characterized as the previously reported (*S, S*)-trypyl-valyl piperazine (**7**) (Fig. 2b, trace-ii, and Fig. 2e) through NMR analysis (Table S10 and Figures S52–S56).

### Functional characterization of the tailoring enzymes in the *flz* gene cluster

**Fig. 3:**
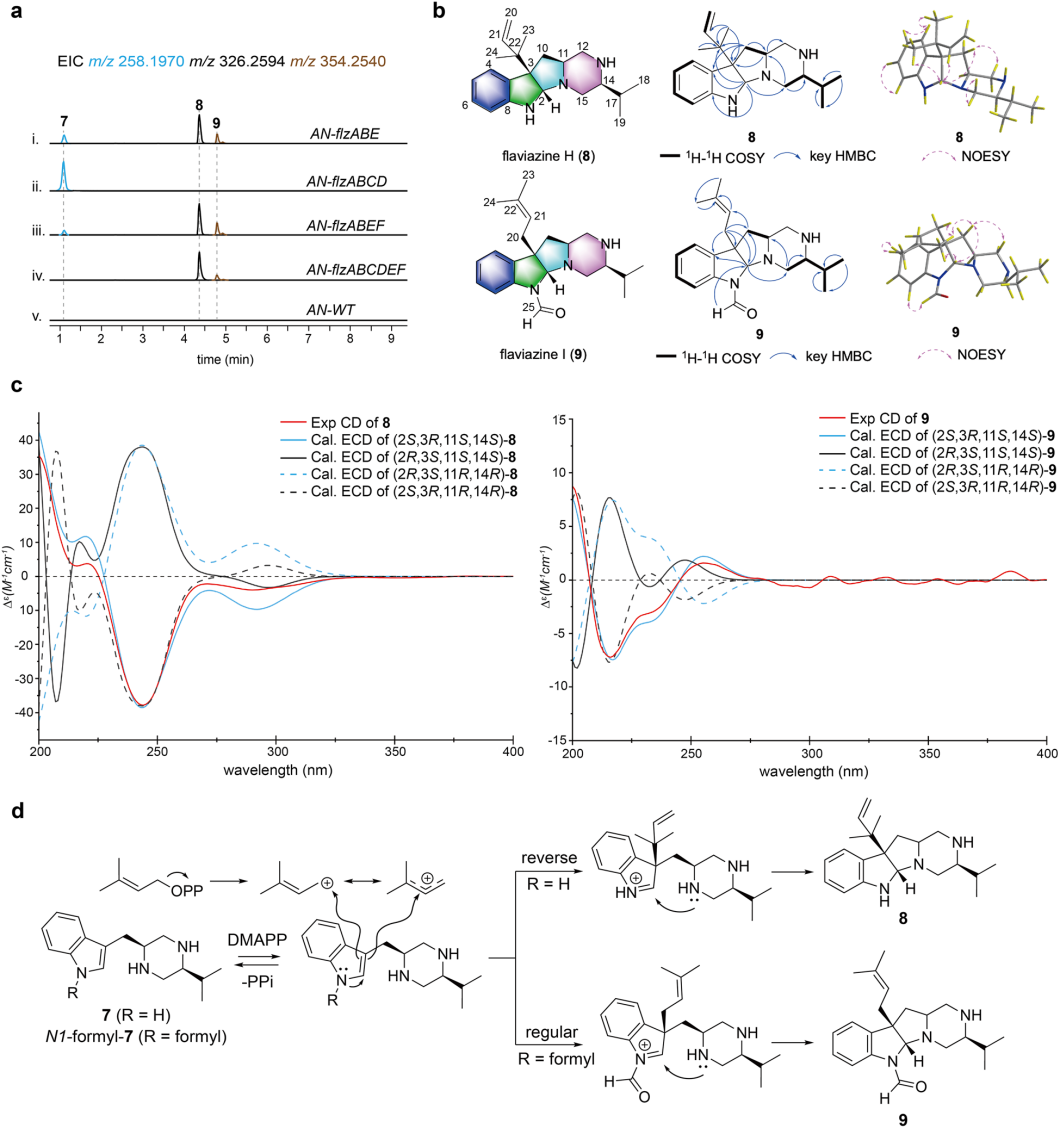
**a** LC–MS analysis of the *A. nidulans* control and the different combination transformants. **b** Structures of compounds **8** and** 9**, along with key ^1^H–^1^H COSY, HMBC, and NOESY correlations. **c** Experimental and calculated ECD spectra of **8** and **9** in MeOH. **d** Proposed catalytic mechanism of FlzE for the formation of compounds **8** and **9**

After confirming the core genes involved in synthesizing the piperazine backbone compound **7**, we focus on the modification steps catalyzed by post-tailoring enzymes within the *flz* cluster. The DMATS-encoding gene *flzE*, unique to the *flz* cluster compared to other piperazine BGCs, was first introduced into the *AN-flzAB* strain. LC–MS analysis of the extracts from the *AN-flzABE* strain, compared to the *AN-flzAB* control, revealed two new peaks at *m/z* 326.2594 ([M + H]^+^, **8**) and 354.2545 ([M + H]^+^, **9**) (Fig. 3a, trace i). The observed quasi-molecular ion peak of **8**, which is 68 Da higher than **7** (*m/z* 258.1970) suggested a molecular formula of C_21_H_31_N_3_, indicating the presence of a dimethylallyl group in compound **8**. The predicted molecular formula of **9** (C_22_H_31_N_3_O, 28 Da more than **8**) suggested it was a formyl-substituted **8**. Following large-scale fermentation and purification, the structures of **8** and** 9** were determined through extensive NMR analysis and ECD calculations (Fig. 3b, c).

The ^1^H and ^13^C NMR data (Table 1 and Figures S57–S65) of **8**, assigned by DEPT and HSQC experiments, exhibited signals for two singlet methyl groups at *δ*_H_ 0.92 (3H, H-23) and 1.05 (3H, H-24), one olefin proton at *δ*_H_ 5.99 (H-21), and two terminal olefin protons at *δ*_H_ 5.08 (2H, H-20), along with corresponding carbon resonances at *δ*_C_ 114.0 (C-20), 146.0 (C-21), 42.6 (C-22), 19.3 (C-23), and 18.7 (C-24). These data confirmed the “reverse” attachment of the dimethylallyl moiety to the backbone structure. Compared to the ^13^C NMR spectrum of compound 7, the absence of a carbon–carbon double bond signals between C-2 and C-3 in the indole ring, along with one more degree of unsaturation compared to **7**, suggests the formation of an extra ring. In the HMBC experiment, correlations from H-2 (*δ*_H_ 4.13) to C-15 (*δ*_C_ 50.8) in the piperazine ring and to the *sp*^3^ quaternary C-22 in the dimethylallyl group, from H-11 (*δ*_H_ 2.55), CH_3_-21, and CH_3_-22 to the *sp*^3^ quaternary C-3 (*δ*_C_ 62.8), from H-10 (*δ*_H_ 1.62 and 2.52) to C-22 revealed the formation of a C-N bond between C-2 and N-16 and the attachment of the dimethylallyl moiety to C-3. The relative configuration of C-2 and C-3 was determined by analyzing the NOESY spectrum, where key correlations between H-2 and CH_3_-22, as well as H-2 and CH_3_-23, indicated that the dimethylallyl moiety and H-2 were co-facial (Fig. 3b). To determine the absolute configuration of **8**, we compared the experimental ECD spectra with their TD-DFT-calculated values. Four stereoisomers of compound **8** (2*S*/3*R*/11*S*/14*S*, 2*R*/3*S*/11*S*/14*S*, 2*R*/3*S*/11*R*/14*R*, and 2*S*/3*R*/11*R*/14*R*) were subjected to ECD calculation using Gaussian 09 program with the TD-DFT-B3LYP/6–311 + + G(d,p) level of theory on a B3LYP/6–311 + + G(d) optimized geometry through the polarizable conductor calculation model (SMD) in MeOH [25]. The ECD spectrum of 2*S*,3*R*,11*S*,14*S*-**8** matched well with the experimental spectrum (Fig. 3c). Thus, the structure of **8**, named flaviazine H, featuring a novel 6–5-5–6 tetracyclic piperazine scaffold, was deduced as shown. The proposed biosynthetic mechanism of **8** commences with a reverse prenylation at the C-3 position, which triggers an intramolecular electronic rearrangement, generating a key imine intermediate. Subsequent nucleophilic attack by the lone pair electrons on N-16 at the C-2 position of the imine results in C–N bond formation (Fig. 3d).

**Table 1:** ^1^H NMR (600 MHz; *δ* in ppm, *J* in Hz) and ^13^C NMR (125 MHz) data of compounds **8** and** 9** (in CD_3_OD)

No.	**8**		**9**	
	*δ*_H_ (mult, *J*)	*δ*_C_	*δ*_H_ (mult, *J*)	*δ*_C_
2	4.13 (s)	86.0	4.80^2^	85.1
3		62.8		55.8
4	7.10 (d, 6.9)	126.7	7.33 (d, 7.9)	112.0
5	6.72 (m)	119.8	7.29 (t, 7.5)	125.7
6	7.05 (m)	129.0	7.26 (t, 7.7)	129.3
7	6.62 (d, 6.6)	111.7	7.17 (d, 7.4)	126.5
8		151.4		148.9
9		135.9		140.2
10	2.52 (m^a^)	38.7	2.44 (m^b^)	38.2
	1.62 (d, 11.8)		2.34 (d, 12.1)	
11	2.55 (m^a^)	57.7	2.80 (m)	58.0
12	2.62 (dd, 11.8, 4.6)	49.7	2.64 (m)	49.6
	3.39 (m^1^)		3.39 (m)	
14	2.90 (m)	62.1	2.80 (m)	62.2
15	2.29 (m)	50.8	2.49 (m^b^)	52.3
	3.53 (br.d, 12.0)		3.68 (d, 13.1)	
17	1.87 (m)	30.9	1.85 (m)	30.6
18	1.05 (d, 6.9)	19.3	1.05 (d, 6.8)	19.3
19	1.05 (d, 6.9)	18.7	1.01 (d, 6.9)	18.8
20	5.08 (dd, 17.6 14.2)	114.0	2.34 (d, 12.4)	41.0
	5.04 (dd, 17.6, 14.2)		1.72 (m)	
21	5.99 (br.t, 14.3)	146.0	6.72 (t, 7.2)	119.5
22		42.6		136.8
23	0.92 (s)	23.0	1.53 (s)	18.2
24	1.05 (s)	23.0	1.62 (s)	26.0
25			8.60 (s)	162.1

^a,b^Overlapped signals

^1^Overlapped by solvent peak

^2^Overlapped by water peak

Comparing the NMR spectra of **9** with those **8**, the presence of a formyl group in **9** was confirmed by the identification of an additional proton resonance at *δ*_H_ 8.60 (H-25) and its corresponding carbon signal at *δ*_C_ 162.1 (C-25) (Table 1 and Figures S66-S74). Moreover, the substitution of the formyl group at the N-1 position was inferred from the HMBC correlation between H-25 and C-2 (*δ*_C_ 85.1). Another significant difference between the structures of **9** and **8** is that the dimethylallyl moiety was added to the C-3 position in a regular manner, as indicated by the proton signals of two deshielded singlet methyl at *δ*_H_ 1.53 (3H, H-23) and 1.62 (3H, H-24), one methylene at *δ*_H_ 1.72 (1H, Ha-20) and 2.34 (1H, Hb-20), and one olefin at *δ*_H_ 6.72 (H-21) in the ^1^H NMR spectrum of **9**. The co-facial orientation of H-2 and the dimethylallyl moiety was deduced from the key correlation between H-2 (*δ*_H_ 4.80) and H-23 observed in the NOESY experiment (Fig. 3b). The absolute configuration of **9** was determined using the same procedure as that of **8**. Four candidate stereoisomers of compound **9** (2*S*/3*R*/11*S*/14*S*, 2*R*/3*S*/11*S*/14*S*, 2*R*/3*S*/11*R*/14*R*, and 2*S*/3*R*/11*R*/14*R*) were applied to ECD calculation, and the ECD spectrum of 2*S*,3*R*,11*S*,14*S*-**9** matched well with the experimental spectrum (Fig. 3c). Thus, the structure of **9**, namely, flaviazine I, was deduced as shown. The biosynthesis of **9** follows a similar mechanism to that of **8**, with the differences that the substrate of prenylation in **9** is a formylated **7** and the double bond undergoes nucleophilic attack at the C-1 carbon cation of the dimethylallyl moiety (Fig. 3d).

To explore the function of the remaining tailoring enzymes within the *flz* cluster, three *A.nidulans* strains, *AN-flzABCD*, *AN-flzABEF*, and *AN-flzABCDEF*, were engineered. However, LC–MS analysis of the three strains revealed no new peaks (Fig. 3a, trace ii–v), indicating that the methyltransferase (FlzF) and CYP450s (FlzC and FlzD) encoding genes in *flz* are pseudogenes not involved in the biosynthetic pathway. This observation reveals a distinct biosynthetic route of the *flz* gene cluster from other known piperazine BGCs, where methyltransferase and oxygenase play essential roles in piperazine derivatives biosynthesis [16]. The likely reason for the uniqueness is that during a horizontal gene transfer event, the *flzE* gene was translocated into the piperazine biosynthetic gene cluster of *A. flavipes*. Over long-term evolution, the epistatic selective pressure exerted by FlzE led to the progressive loss of activity in other tailoring genes [26, 27].

### In vitro enzymatic assays of FlzE expand the chemical space of prenylated alkaloids

**Fig. 4:**
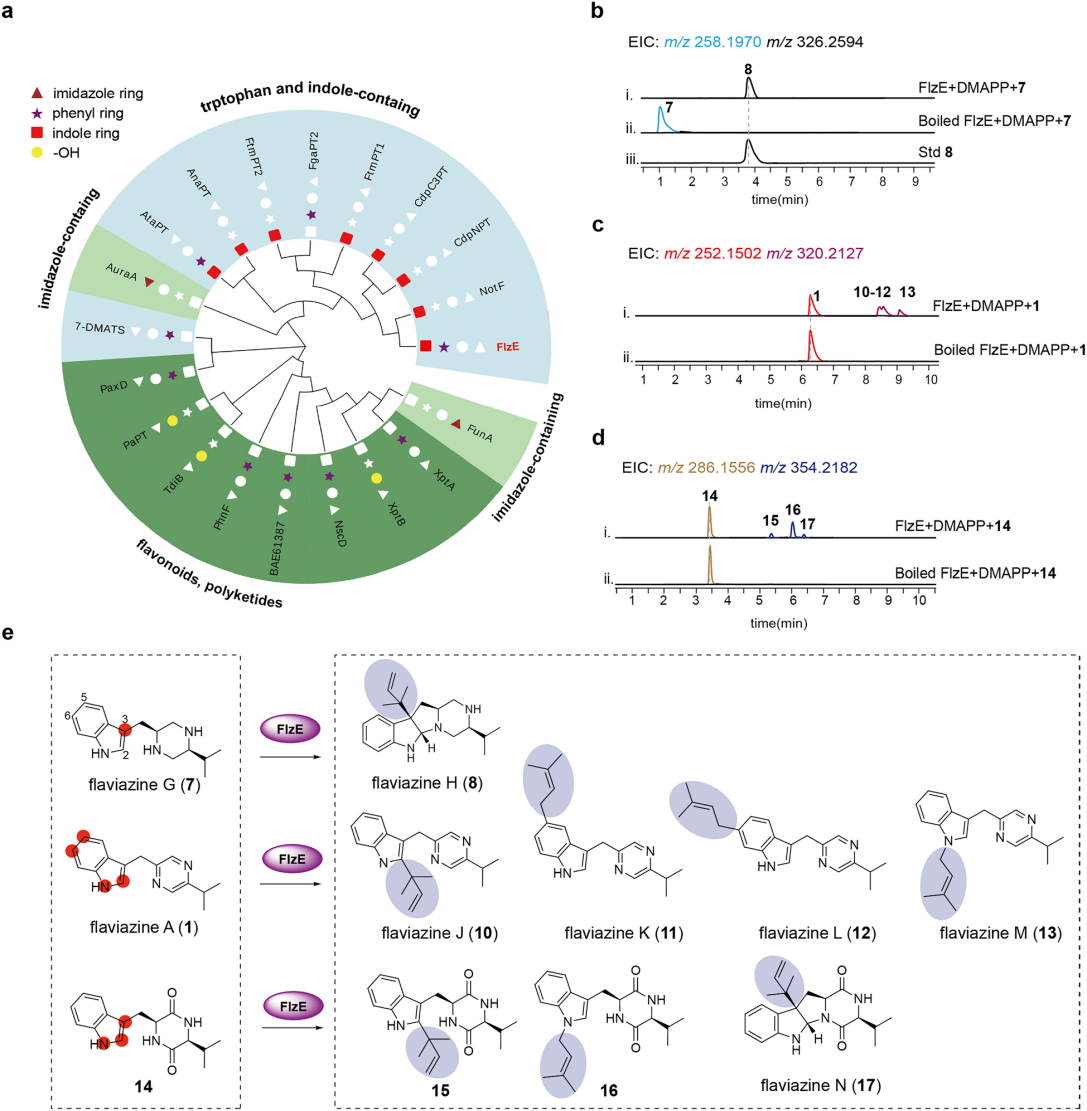
**a** Phylogenetic tree analysis of FlzE. The symbols denoted the structural units of prenyl modification. **b** LC–MS analysis of the in vitro assays of FlzE toward compound **7***.* **c** LC–MS analysis of the in vitro assays of FlzE toward compound **1***.* **d** LC–MS analysis of the in vitro assays of FlzE toward compound **14**. **e** The substrates FlzE catalyzed and their corresponding products. Compounds **10**–**13** and **17** were new

The discovery of reverse and regular prenylation manners in compounds **8** and **9**, respectively, prompted us to further explore the catalytic potential of FlzE. Phylogenetic analysis of FlzE alongside known DMATSs showed that FlzE clustered with DMATSs that predominantly prenylate indole rings using tryptophan or indole-containing compounds as acceptors, consistent with our experimental observations (Fig. 4a and Figure S2). It is well-documented that certain DMATSs, such as AtaPT[11] and AnaPT [28], are capable of prenylating not only native substrates but also non-native molecules with diverse structures at multiple sites, thereby diversifying the range of prenylated products. To expand the chemical space of tryptophan-valine-derived alkaloid derivatives, we employed natural pyrazine (**1**) and chemically synthesized diketopiperazine (**14**) as substrates to evaluate the catalytic versatility of FlzE.

We first utilized piperazine **7** as the native substrate to assess the feasibility of in vitro enzymatic catalysis. Recombinant N-terminally His-tagged FlzE, expressed and purified from *Escherichia coli* (Figure S3), was incubated with **7** in the presence of the co-substrate dimethylallyl diphosphate (DMAPP). LC–MS analysis of the reaction mixtures showed complete consumption of substrate **7** and the exclusive production of the prenylated product **8** (Fig. 4b)*.* Subsequently, when substrate **1** was incubated with FlzE and DMAPP, LC–MS analysis revealed multiple peaks with an identical quasi-molecular ion at *m/z* 320.2127 ([M + H]^+^, 68 Da more than **1**) (Fig. 4c). Following a large-scale incubation, four previously unreported prenylated pyrazine alkaloids, namely flaviazine J (**10**), flaviazine K (**11**)**,** flaviazine L (**12**), and flaviazine M (**13**), were purified and identified (Tables S11–S14, and Figs. 4e, S75–S106). Compound **10** exhibited reverse-prenylation at the C-2 position of **1**, while **11**, **12**, and **13** were regularly prenylated at C-5, C-6, and the N-1 positions, respectively.

LC–MS analysis of the incubation mixture containing FlzE, DMAPP, and **14** revealed three mass peaks (**15**–**17**) with an identical adduct ion at *m/z* 354.2182 ([M + H]^+^, 68 Da more than **14**), with only minor substrate consumption (Fig. 4d). Large-scale incubation and purification yielded three compounds, which were identified as one new prenylated diketopiperazine (**17**) and two known ones (**15** and **16**), by extensive NMR analysis and ECD calculation (Tables S16-S18, and Figs. 4e, S108–S124). Compound **15** featured a reverse-prenylated moiety at the C-2 position, and **16** was regularly prenylated at the N-1 position. Notably, compound **17**, named flaviazine N, was identified as a reverse-prenylated diketopiperazine alkaloid with a 6/5/5/6 ring system.

To elucidate the mechanistic basis of FlzE’s versatility, we compared FlzE with other DMATSs that catalyze tryptophan or indole-containing compounds and analyzed their reported crystal structures (Figure S4). The results showed that DMATS possesses highly conserved amino acid residues, including a tyrosine shield required for the reaction environment (Y188, Y257, Y340, Y398, and Y409 in FlzE) and conserved glutamate (E85 in FlzE) that forms a hydrogen bond with the N-1 atom of indole [1, 23]. In contrast, residues that interact with substrates are not conserved in different DMATS (S92, M94, W182, and Y205 in FtmPT1; T108 in CdpNPT; and I100 in NotF) [23, 29, 30]. These non-conserved residues, along with flexible loop regions, may suggest that different substrates adopt distinct conformations in the active pocket of FlzE, leading to variations in the distance between the active sites of indole and the C-1/C-3 of the prenyl donor. Collectively, these structural features provide a plausible explanation for the observed divergence in the mechanism of FlzE prenylation.

## Conclusion

This study represented the identification and activation of a distinctive BGC for DMATS-containing piperazine alkaloid. Through heterologous expression and in vitro enzymatic assays, the silent gene cluster *flz* was successfully activated, leading to the isolation and identification of five novel tryptophan-valine-derived alkaloids and seven previously undescribed prenylated analogs. The core biosynthetic enzymes, FlzA and FlzB, were shown to utilize two structurally distinct amino acid precursors to synthesize diverse alkaloid scaffolds, including piperazine, pyrazine, and pyridine. Functional studies unveiled FlzE as an unusual DMATS: (1) it specifically prenylated the indole C-3 position of native piperazine substrates, triggering intramolecular cyclization to form novel skeletons, such as compounds **8** and **9**, which feature a 6–5-5–6 ring system; (2) it facilitated multi-site prenylation of non-native pyrazines and diketopiperazines, producing a variety of prenylated derivatives. However, the enzymatic mechanism underlying its versatile prenylation capability warrants further exploration.

Based on the known activities of structurally similar compounds, such as tryprostatin B with anti-tubulin activity and fumitremorgin C as a potent and specific chemosensitizing agent [31, 32], these new prenylated alkaloids discovered in this study expanded the chemical space of indole alkaloids and enriched the pool of chemical candidates for innovative drug discovery. Furthermore, the exploration of FlzE’s catalytic properties lays the groundwork for elucidating its enzymatic mechanism and opens the potential to engineer this enzyme into a precise prenylation biocatalyst.

## Experimental section

### Strains and culture conditions

All strains and plasmids used and generated in this study are listed in Table S1. *A. flavipes* (QM507) was derived from the intertidal zone of the Yangtze River in Wuhan, Hubei Province, China. The sequence data for this strain have been submitted to the EMBL/GenBank under accession No. KP339510. A voucher sample (ID: QM507) was preserved in the herbarium of the Huazhong University of Science and Technology, China [33]. To extract genomic DNA (gDNA), *A. flavipes* was cultured in PDB liquid medium (4 g/L potato starch, 20 g/L dextrose, Becton, Dickinson and Company, USA) at 28 °C for seven days. *Aspergillus nidulans* LO8030 was used as the host for the heterologous expression of the* flz* gene cluster. *A. nidulans* was cultured on solid CD medium (10 g/L glucose, 50 ml/L 20× nitrate salts, 1 ml/L trace elements, 20 g/L agar) at 37 °C for 3–4 days for sporulation, or in liquid or CD-ST medium (20 g/L starch, 20 g/L tryptone, 50 ml/L 20× nitrate salts, 1 ml/L trace elements) at 28 °C for 4 days for heterologous expression and compounds production. *Saccharomyces cerevisiae* strain BJ5464-NpgA was used as the host for heterologous recombination to construct the *A. nidulans* overexpression plasmids*. S. cerevisiae* was grown in yeast peptone dextrose (YPD) medium (20 g/L glucose, 20 g/L tryptone, 10 g/L yeast extract) at 30 °C. *Escherichia coli* DH5α was used for plasmid construction, *Escherichia coli* BAP1 was used for FlzA expression [34], and *Escherichia coli* BL21 for protein expression. All *E. coli* strains were cultured at 37 °C for cloning or 16 °C for protein expression.

### Chemicals and chemical analysis

All LC–MS analysis was performed on an Agilent 1290 HPLC system equipped with a 6545 Q-TOF spectrometer (Agilent Eclipse Plus, C_18_ column, 3.5 µm, 100 × 2.1 mm, gradient CH_3_CN/H_2_O (with 0.1% formic acid) = 5/95 ~ 98/2, 0.0 ~ 11.0 min; isocratic CH_3_CN/H_2_O (with 0.1% formic acid) = 100/0, 11.0 ~ 13.0 min; isocratic CH_3_CN/H_2_O (with 0.1% formic acid) = 5/95, 13.0 ~ 17.0 min, flow rate = 0.5 mL/min), using positive mode electrospray ionization. Data were evaluated with the Agilent MassHunter Qualitative Analysis B.07.00 software. Analysis of secondary metabolites was performed on an Agilent series 1220 HPLC (Agilent Technologies, USA) with an Agilent ZORBAX SB-C_18_ column (250 × 4.6 mm, 5 µm). Semi-preparative HPLC was performed on an Agilent series 1220 HPLC (Agilent Technologies, USA) with an Agilent ZORBAX SB-C_18_ column (250 × 9.4 mm, 5 µm). ^1^H, ^13^C, and 2D NMR spectra were obtained at a Bruker AM-600 NMR spectrometer. Silica gel (100–200 mesh, 200–300 mesh, Qingdao Marine Chemical Inc., China) was used in the chromatography processes.

### Construction of *A. nidulans* expression plasmids

For the construction of *A. nidulans* expression plasmids, fragments of *flzA–E* were amplified from gDNA with two homologous arms by PCR. The *glaA*, *gpdA*, *amyB* promoters were amplified from different vectors pYTU, pYTR, and pYTP, respectively, using primer pairs *glaA*-F/R, *gpdA*-F/R, and *amyB*-F/R. Plasmid pYTU was digested with NotI and SmiI, and plasmids pYTR and pYTP were digested with BamHI and SmiI to serve as vectors for gene insertion. Expression plasmids were constructed via yeast homologous recombination in *S. cerevisiae* BJ5464-NpgA. Circular plasmids were then extracted from yeast and transformed into *E. coli* DH5α strain to obtain purified plasmids for transformation.

### Construction of *A. nidulans* expression plasmids

*A. nidulans* was cultured on solid CD medium containing 10 mM uridine, 5 mM uracil, 1 μg/mL pyridoxine HCl, and 0.25 μg/mL riboflavin at 37 °C for 4 days. Spores were collected in 10% glycerol and inoculated into 30 mL of liquid CD medium. The culture was then incubated at 37 °C and 250 rpm for 6 h. After germination, cultures were centrifuged at 4 °C and 7,000 rpm for 10 min to harvest mycelia. The mycelia were washed twice with 15 mL of osmotic buffer (1.2 M MgSO_4_·7H_2_O, 10 mM sodium phosphate, pH 5.8) at 4 °C, 5,000 rpm for 10 min, and then resuspended in 10 mL of osmotic buffer containing 30 mg of Lysing Enzymes (Sigma) and 20 mg of Yatalase (Takara). The suspension was transferred into a 50 mL Erlenmeyer flask and cultured at 37 °C, 80 rpm for 4 h. The culture fluid was poured into a sterile 50 mL centrifugal tube and gently overlaid with 10 mL of trapping buffer (0.6 M sorbitol, 0.1 M Tris–HCl, pH 7.0), and centrifuged again at 4 °C, 5,000 rpm for 15 min. The protoplast layer was transferred and dispersed into twice the volume of STC buffer (1.2 M sorbitol, 10 mM CaCl_2_, 10 mM Tris–HCl, pH 7.5), and then centrifuged at 4 °C, 5,000 rpm for 10 min. The supernatant was removed, and STC buffer was added to resuspend the protoplast for transformation. To obtain heterologous expression strains in *A. nidulans*, 10 µL of plasmids (pZ7301-73) was added to 100 µL of *A. nidulans* protoplast. After incubation on ice for 60 min, 1.25 mL of 60% PEG solution was added, and the mixture was incubated at room temperature for 20 min. The mixture was cultured on regeneration dropout solid CD-SD medium (CD medium supplemented with 1.2 mM sorbitol) at 37 °C for 2–3 days to induce sporulation. Transformants were transferred to solid CD medium at 37 °C for 3–4 days. Spores were then incubated on solid CD-ST medium (20 g/L starch, 20 g/tryptone, 50 ml/L nitrate salts, 1 ml/L trace elements and 20 g/L agar) at 28 °C for 4 days or in liquid CD-ST medium (20 g/L starch, 20 g/tryptone, 50 ml/L nitrate salts and 1 ml/L trace element) at 28 °C and 250 rpm for 4 days. Products from all combination strains for *A. nidulans* heterologous expression were extracted with methanol, dried in vacuo, and dissolved in methanol for LC–MS analysis.

### Construction of plasmid of His-tagged FlzE and expression in *E. coli*

The gene *flzE* was amplified from cDNA of *A. flavipes* by using primer pair pET28a-FlzE-F/R. The EcoRI and XhoI-digested fragments of the pET28a plasmid were subjected to Gibson assembly (NEBuilder HiFi DNA Assembly Master Mix, New England BioLabs) with *flzE* to construct pET28a-FlzE. The plasmid was introduced into *E. coli* BL21 (DE3) competent cell via heat shock transformation. A single colony was then cultured overnight in Luria–Bertani (LB) medium supplemented with 50 µg/mL kanamycin at 37 °C. Cells were then transferred to fresh LB medium containing 50 µg/mL kanamycin and cultured at 37 °C until an optical density at OD600 of 0.6 was reached. At this point, 1 mol/L Isopropyl β-dthiogalactopyranoside (IPTG) was added to achieve a final concentration of 0.5 mmol/L, and the cultures were then continued for an additional 20 h at 16 °C to induce the target protein expression. All purification steps were carried out at 4 °C. The cultured cells were resuspended in a lysis buffer (pH 8.0) composed of 50 mmol/L NaH_2_PO_4_·H_2_O, 10% (v/v) glycerol, 300 mmol/L NaCl, and 10 mmol/L imidazole. Cells were lysed by sonication, and the insoluble debris was removed by centrifugation at 12,000 g for 30 min. The supernatant was then loaded onto a Ni–NTA Resin (Thermo Fisher Scientific) column. The resin was washed with 50 column volumes of Lysis buffer containing 40 mmol/L imidazole, after which the target protein was eluted with Lysis buffer containing 250 mmol/L imidazole. The protein solution was concentrated using Amicon Ultra-15 centrifugal filter devices (10K MWCO, Millipore). The target protein was subsequently eluted in a buffer composed of 50 mmol/L Tris–HCl (pH 7.5) and 10% (v/v) glycerol and stored at −80 °C. Protein purity was assayed by SDS-PAGE, and the results are summarized in Figures S3. Protein concentration was determined using a BCA protein quantification kit (Shanghai Beyotime Biotechnology Co., Ltd).

### In Vitro assays of FlzE

To investigate the function of FlzE, an enzymatic assay (with a total volume of 100 µL) was conducted using a mixture composed of Tris–HCl buffer (50 mM, pH 7.5), 1 mM DMAPP, 1 mM **10** and 10 µM FlzE. The assay was incubated at 37 °C for 12 h. Subsequently, 200 µL of methanol was added to quench the reaction. Following centrifugation at 13,300 rpm for 30 min, the resulting supernatant was analyzed using LC–MS.

### Chemical synthesis of compound 14

To verify the substrate promiscuity of FlzE, we attempted to synthesis compound **14** via chemical methods. To a solution of protected-amino acid (1equiv) and tryptophan methyl ester hydrochloride (1equiv) in dry DCM (0.2 M) was added DIPEA (2 equiv), HOBt (1.1 equiv), and EDCI‧HC1 (1.2 equiv) at 0 ℃. After 1 h, the mixture was warmed to room temperature and stirred overnight. Water was added and the mixture was extracted with DCM. The combined organic layer was washed with 10% HCl, sat. NaHCO_3_, and brine, dried over anhydrous Na_2_SO_4_, and concentrated. The resulting residue was purified by silica gel flash chromatography (hexanes/ethyl acetate) to give the dipeptide. A solution of amide (2.0 mmol) in dry CH_2_Cl_2_ was treated with HCOOH at rt for 3 h. Solvent was then evaporated and the reaction mixture was dissolved in 2-butanol:toluene (3:1) followed by addition of triethylamine. The mixture was allowed to reflux for 16 h. After the evaporation of solvent, diketopiperazines precipitated as a white solid, which was filtered off, washed with MeOH, and used for next step without further purification.

### Isolation and purification of compounds

The *AN-flzA* recombinant was cultured in 8 L solid CD-ST medium at 28 °C for 7 days, and the culture was extracted with methanol three times. The organic solvent was evaporated to dryness under vacuum to obtain the crude extracts (130.0 g), which were subjected to silica gel column chromatography (200–300 mesh) using a DCM/MeOH gradient elution (150:1, 100:1, 50:1, 10:1, 5:1, 1:1, 1:2) to obtain seven fractions (Frs. 1–7). Fraction 1 (DCM/MeOH 150:1) was purified by semi-preparative HPLC equipped with an Agilent ZORBAX SB-C_18_ column to yield **1** (20 mg; ACN–H_2_O (with 0.1% formic acid), 60:40, v/v, 2.0 mL/min, *t*_*R*_ = 8 min). Fraction 3 (DCM/MeOH 50:1) was fractionated on a silica gel column eluted with DCM/MeOH (100:1, 80:1, 50:1) to yield three major fractions (Fr.3.1–Fr.3.3). Fraction 3.3 (DCM/MeOH 50:1) was purified by semi-preparative HPLC equipped with Agilent ZORBAX SB-C_18_ column (ACN–H_2_O (with 0.1% formic acid), 35:65, v/v, 2.0 mL/min) to yield **2** (9 mg; *t*_*R*_ = 17 min) and **3** (3 mg; *t*_*R*_ = 25 min). Fraction 5 (DCM/MeOH 5:1) was fractionated on a silica gel column eluted with DCM/MeOH (25:1, 10:1, 8:1, 5:1) to yield four major fractions (Fr.5.1–Fr.5.4). Fraction 5.4(DCM/MeOH 5:1) was purified by semi-preparative HPLC equipped with Agilent ZORBAX SB-C_18_ column (ACN–H_2_O (with 0.1% formic acid), 30:70, v/v, 2.0 mL/min) to yield **4** (8 mg; *t*_*R*_ = 17 min) and **5** (3 mg; *t*_*R*_ = 26 min). Fraction 6 (DCM/MeOH 1:1) was fractionated on a silica gel column eluted with DCM/MeOH (10:1, 5:1, 2:1,1;1) to yield four major fractions (Fr.6.1–Fr.6.4). Fraction 6.4(DCM/MeOH 1:1) was purified by semi-preparative HPLC equipped with Agilent ZORBAX SB-C_18_ column to yield **6** (1.4 mg; ACN–H_2_O (with 0.1% formic acid), 20:80, v/v, 2.0 mL/min, *t*_*R*_ = 10 min).

The *AN-flzAB* recombinant was cultured in 4 L solid CD-ST medium at 28 °C for 7 days, and the culture was extracted with methanol three times. The organic solvent was evaporated to dryness under vacuum to obtain the crude extracts (30.0 g), which were subjected to silica gel column chromatography (200–300 mesh) using a DCM/MeOH gradient elution (10:1, 5:1, 1:1, 1:2, 1:5) to obtain seven fractions (Frs. 1–5). Fraction 4 (DCM/MeOH 1:2) was purified by semi-preparative HPLC equipped with Agilent ZORBAX SB-C_18_ column to yield **7** (20 mg; ACN–H_2_O (with 0.1% formic acid), 10:90, v/v, 2.0 mL/min, *t*_*R*_ = 6 min).

The *AN-flzABE* recombinant was cultured in 4 L solid CD-ST medium at 28 °C for 7 days, and the culture was extracted with methanol three times. The organic solvent was evaporated to dryness under vacuum to obtain the crude extracts (30.0 g), which were subjected to silica gel column chromatography (200–300 mesh) using a DCM/MeOH gradient elution (50:1, 10:1, 5:1, 1:1) to obtain four fractions (Frs. A-D). Fraction D (DCM/MeOH 5:1) was purified by semi-preparative HPLC equipped with Agilent ZORBAX SB-C_18_ column (ACN–H_2_O (with 0.1% formic acid), 38:62, v/v, 2.0 mL/min) to yield **8** (1 mg; *t*_*R*_ = 18 min) and **9** (0.9 mg; *t*_*R*_ = 22 min).

The isolation and purification of compounds **10** − **13** was conducted using a mixture composed of Tris–HCl buffer (50 mM, pH 7.5), 1 mM **1** and 10 µM FlzE. The assay was incubated at 37 °C for 12 h. Subsequently, methanol was added to quench the reaction, and the culture was extracted with ethyl acetate three times. The organic solvent was evaporated to dryness under vacuum to obtain the crude extracts. Which was purified by semi-preparative HPLC equipped with Agilent ZORBAX SB-C_18_ column (ACN–H_2_O (with 0.1% formic acid), 75:25, v/v, 2.0 mL/min) to yield to yield **10** (1 mg; *t*_*R*_ = 13 min), **11** (0.9 mg; *t*_*R*_ = 20 min), **12** (1.2 mg; *t*_*R*_ = 22 min), **13** (0.8 mg; *t*_*R*_ = 23 min).

Compounds **15** − **17** were isolated and purified from an enzymatic assay, conducted using a mixture composed of Tris–HCl buffer (50 mM, pH 7.5), 1 mM **14** and 10 µM FlzE. The assay was incubated at 37 °C for 12 h. Subsequently, methanol was added to quench the reaction, and the culture was extracted with ethyl acetate three times. The organic solvent was evaporated to dryness under vacuum to obtain the crude extracts. Which was purified by semi-preparative HPLC equipped with Agilent ZORBAX SB-C_18_ column (ACN–H_2_O (with 0.1% formic acid), 55:45, v/v, 2.0 mL/min) to yield **15** (2 mg; *t*_*R*_ = 13 min), **16** (3 mg; *t*_*R*_ = 20 min), **17** (2 mg; *t*_*R*_ = 22 min).

Flaviazine B (**2**): yellow powder, UV (MeOH) *λ*_max_ (Abs) = 220 (0.67), 273 (0.31) nm; IR (KBr) *ν*_max_ = 3361, 2920, 2850, 1631, 1457, 1357, 1340, and 734 cm^−1^; ^1^H and ^13^C NMR data see Table S5 and Figures S14-S21; HRMS (ESI-TOF) *m/z*: [M + H]^+^ 268.1463 (calcd for C_16_H_18_N_3_O, 268.1450).

Flaviazine C (**3)**: yellow powder; ^1^H and ^13^C NMR data see Table S6 and Figures S22-S26; HRMS (ESI-TOF) *m/z*: [M + H]^+^ 268.1472 (calcd for C_16_H_18_N_3_O, 268.1450).

Flaviazine D (**4**): yellow powder, UV (MeOH) *λ*_max_ (Abs) = 210 (0.79), 270 (0.29) nm; IR (KBr) *ν*_max_ = 3359, 3203, 1772, 1622, 1471, 1038, and 751 cm^−1^; ^1^H and ^13^C NMR data see Table S7 and Figures S27-S34; HRMS (ESI-TOF) *m/z*: [M + H]^+^ 284.1421 (calcd for C_16_H_18_N_3_O_2_, 284.1399).

Flaviazine E (**5**): brown powder, UV (MeOH) *λ*_max_ (Abs) = 200 (0.74), 220 (0.73), 282 (0.21), 315 (0.25) nm; IR (KBr) *ν*_max_ = 3364, 2920, 2850, 1606, 1424, and 741 cm^−1^; ^1^H and ^13^C NMR data see Table S8 and Figures S35-S43; HRMS (ESI-TOF) *m/z*: [M + H]^+^ 253.1365 (calcd for C_16_H_17_N_2_O, 253.1341).

Flaviazine F (**6**): white powder, UV (MeOH) *λ*_max_ (Abs) = 203 (0.36), 220 (0.54), 280 (0.09) nm; IR (KBr) *ν*_max_ = 3255, 2920, 2850, 1671, 1595, 1433, 1399, and 736 cm^−1^; ^1^H and ^13^C NMR data see Table S9 and Figures S44-S51; HRMS (ESI-TOF) *m/z*: [M + H]^+^ 291.1689 (calcd for C_16_H_23_N_2_O_3_, 291.1709).

Flaviazine H (**8**): white powder, UV (MeOH) *λ*_max_ (Abs) = 210 (0.53), 245 (0.27), 295 (0.10) nm; IR (KBr) *ν*_max_ = 3391, 3365, 2850, 1646, 1632, 1604, and 1384 cm^−1^; ^1^H and ^13^C NMR data see Table 1 and Figures S57-S65; HRMS (ESI-TOF) *m/z*: [M + H]^+^ 326.2596 (calcd for C_21_H_32_N_3_, 326.2596).

Flaviazine I (**9**): white powder, UV (MeOH) *λ*_max_ (Abs) = 210 (0.28), 247 (0.16), 287 (0.09) nm; IR (KBr) *ν*_max_ = 3365, 2920, 2850, 1631, 1598, 1384, and 1357 cm^−1^; ^1^H and ^13^C NMR data see Table 1 and Figures S66-S74; HRMS (ESI-TOF) *m/z*: [M + H]^+^ 354.2558 (calcd for C_22_H_32_N_3_O, 354.2545).

Flaviazine J (**10**): brown powder, UV (MeOH) *λ*_max_ (Abs) = 225 (0.57), 275 (0.29) nm; IR (KBr) *ν*_max_ = 3364, 2959, 2922, 2852, 1660, 1384, 1246, and 744 cm^−1^; ^1^H and ^13^C NMR data see Table S11 and Figures S75-S82; HRMS (ESI-TOF) *m/z*: [M + H]^+^ 320.2131 (calcd for C_21_H_26_N_3_, 320.2127).

Flaviazine K (**11**): brown powder, UV (MeOH) *λ*_max_ (Abs) = 225 (0.21), 275 (0.12) nm; IR (KBr) *ν*_max_ = 3362, 2960, 2921, 2851, 1659, 1631, 1601, 1383, and 1357 cm^−1^; ^1^H and ^13^C NMR data see Table S12 and Figures S83-S90; HRMS (ESI-TOF) *m/z*: [M + H]^+^ 320.2135 (calcd for C_21_H_26_N_3_, 320.2127).

Flaviazine L (**12**): brown powder, UV (MeOH) *λ*_max_ (Abs) = 225 (0.97), 275 (0.42) nm; IR (KBr) *ν*_max_ = 3362, 2964, 2921, 2851, 1659, 1631, 1601, 1384, and 1359 cm^−1^; ^1^H and ^13^C NMR data see Table S13 and Figures S91-S98; HRMS (ESI-TOF) *m/z*: [M + H]^+^ 320.2138 (calcd for C_21_H_26_N_3_, 320.2127).

Flaviazine M (**13**): brown powder, UV (MeOH) *λ*_max_ (Abs) = 225 (0.20), 275 (0.15) nm; IR (KBr) *ν*_max_ = 3364, 2961, 2921, 2851, 1659, 1632, 1601, 1384, 1224, and 741 cm^−1^; ^1^H and ^13^C NMR data see Table S14 and Figures S99-S106; HRMS (ESI-TOF) *m/z*: [M + H]^+^ 320.2130 (calcd for C_21_H_26_N_3_, 320.2127).

Compound **15**: white powder, ^1^H and ^13^C NMR data see Table S16 and Figures S108-S112; HRMS (ESI-TOF) *m/z*: [M + H]^+^ 354.2186 (calcd for C_21_H_28_N_3_O_2_, 354.2182).

Compound **16**: white powder, ^1^H and ^13^C NMR data see Table S17 and Figures S113-S114; HRMS (ESI-TOF) *m/z*: [M + H]^+^ 354.2182 (calcd for C_21_H_28_N_3_O_2_, 354.2182).

Flaviazine N (**17**): white powder, UV (MeOH) *λ*_max_ (Abs) = 210 (1.10), 245 (0.58), 300(0.20) nm; IR (KBr) *ν*_max_ = 3366, 2964, 2925, 1678, 1606, 1466, 1443, 1416, and 745 cm^−1^; ^1^H and ^13^C NMR data see Table S18 and Figures S115-S123; HRMS (ESI-TOF) *m/z*: [M + H]^+^ 354.2205 (calcd for C_21_H_28_N_3_O_2_, 354.2182).

## Supplementary Information

Additional file 1.

Additional file 2.

Additional file 3.

Additional file 4.

Additional file 5.

## Data Availability

The data that support the findings of this study are openly available in the Science Data Bank at.

## References

[1] Miller ET, Tsodikov OV, Garneau-Tsodikova S. Structural insights into the diverse prenylating capabilities of DMATS prenyltransferases. Nat Prod Rep. 2024;41:113–47. 37929638 10.1039/d3np00036b

[2] Yazaki K, Sasaki K, Tsurumaru Y. Prenylation of aromatic compounds, a key diversification of plant secondary metabolites. Phytochemistry. 2009;70:1739–45. 19819506 10.1016/j.phytochem.2009.08.023

[3] Winkelblech J, Fan A, Li S-M. Prenyltransferases as key enzymes in primary and secondary metabolism. Appl Microbiol Biotechnol. 2015;99:7379–97. 26216239 10.1007/s00253-015-6811-y

[4] Mukai R. Prenylation enhances the biological activity of dietary flavonoids by altering their bioavailability. Biosci Biotechnol Biochem. 2018;82:207–15. 29307271 10.1080/09168451.2017.1415750

[5] Gerhauser C, Alt A, Heiss E, Gamal-Eldeen A, Klimo K, Knauft J, et al. Cancer chemopreventive activity of Xanthohumol, a natural product derived from hop. Mol Cancer Ther. 2002;1:959–69. 12481418

[6] Wang Q, Ding Z-H, Liu J-K, Zheng Y-T. Xanthohumol, a novel anti-HIV-1 agent purified from Hops *Humulus lupulus*. Antiviral Res. 2004;64:189–94. 15550272 10.1016/j.antiviral.2004.08.005

[7] Yu W, Gao Y, Zhao Z, Long X, Yi Y, Ai S. Fumigaclavine C ameliorates liver steatosis by attenuating hepatic de novo lipogenesis via modulation of the RhoA/ROCK signaling pathway. BMC Complement Med Ther. 2023;23:288. 37587459 10.1186/s12906-023-04110-9 PMC10428638

[8] Yu W-G, He Y, Chen Y-F, Gao X-Y, Ning W-E, Liu C-Y, et al. Fumigaclavine C attenuates adipogenesis in 3T3-L1 adipocytes and ameliorates lipid accumulation in high-fat diet-induced obese mice. Korean J Physiol Pharmacol. 2019;23:161–9. 31080347 10.4196/kjpp.2019.23.3.161 PMC6488706

[9] Wang W, Wang P, Ma C, Li K, Wang Z, Liu Y, et al. Characterization and structural analysis of a versatile aromatic prenyltransferase for imidazole-containing diketopiperazines. Nat Commun. 2025;16:144. 39747040 10.1038/s41467-024-55537-8 PMC11696170

[10] Tanner ME. Mechanistic studies on the indole prenyltransferases. Nat Prod Rep. 2015;32:88–101. 25270661 10.1039/c4np00099d

[11] Chen R, Gao B, Liu X, Ruan F, Zhang Y, Lou J, et al. Molecular insights into the enzyme promiscuity of an aromatic prenyltransferase. Nat Chem Biol. 2017;13:226–34. 27992881 10.1038/nchembio.2263

[12] Brito AF, Moreira LKS, Menegatti R, Costa EA. Piperazine derivatives with central pharmacological activity used as therapeutic tools. Fundam Clin Pharmacol. 2019;33:13–24. 30151922 10.1111/fcp.12408

[13] Sakamoto K, Tsujii E, Abe F, Nakanishi T, Yamashita M, Shigematsu N, et al. FR901483, a novel immunosuppressant isolated from *Cladobotryum sp.* no. 11231 taxonomy of the producing organism, fermentation, isolation, physico-chemical properties and biological activities. J Antibiot (Tokyo). 1996;49:37–44. 8609083 10.7164/antibiotics.49.37

[14] Mohamed OG, Salim AA, Khalil ZG, Elbanna AH, Bernhardt PV, Capon RJ. Chrysosporazines F-M: P-glycoprotein inhibitory phenylpropanoid piperazines from an Australian marine fish derived fungus, *Chrysosporium sp.* CMB-F294. J Nat Prod. 2020;83:497–504. 31975579 10.1021/acs.jnatprod.9b01181

[15] Enomoto Y, Shiomi K, Hayashi M, Masuma R, Kawakubo T, Tomosawa K, et al. Herquline B, a new platelet aggregation inhibitor produced by *Penicillium herquei* Fg-372. J Antibiot (Tokyo). 1996;49:50–3. 8609085 10.7164/antibiotics.49.50

[16] Wang R, Piggott AM, Chooi Y-H, Li H. Discovery, bioactivity and biosynthesis of fungal piperazines. Nat Prod Rep. 2023;40:387–411. 36374102 10.1039/d2np00070a

[17] Paluka J, Kanokmedhakul K, Soytong M, Soytong K, Yahuafai J, Siripong P, et al. Meroterpenoid pyrones, alkaloid and bicyclic brasiliamide from the fungus *Neosartorya hiratsukae*. Fitoterapia. 2020;142:104485. 31982554 10.1016/j.fitote.2020.104485

[18] He Q, Zhang H-R, Zou Y. A cytochrome P450 catalyzes oxidative coupling formation of insecticidal dimeric indole piperazine alkaloids. Angew Chem Int Ed. 2024;63:e202404000. 10.1002/anie.202404000 38527935

[19] Pham M-T, Yang F-L, Liu I-C, Liang P-H, Lin H-C. Non-heme iron enzymes catalyze heterobicyclic and spirocyclic isoquinolone core formation in piperazine alkaloid biosynthesis. Angew Chem Int Ed. 2024;63:e202401324. 10.1002/anie.202401324 38499463

[20] Wang R, Liang J, Yang W, Vuong D, Kalaitzis JA, Lacey AE, et al. Heterologous biosynthesis of the sterol O-acyltransferase inhibitor helvamide unveils an α-ketoglutarate-dependent cross-linking oxygenase. Org Lett. 2024;26:1807–12. 38393343 10.1021/acs.orglett.3c04310

[21] Blin K, Shaw S, Steinke K, Villebro R, Ziemert N, Lee SY, et al. AntiSMASH 5.0: updates to the secondary metabolite genome mining pipeline. Nucleic Acids Res. 2019;47:W81–7. 31032519 10.1093/nar/gkz310 PMC6602434

[22] Liu Y, Li P, Qi C, Zha Z, Meng J, Liu C, et al. Cryptic piperazine derivatives activated by knocking out the global regulator LaeA in *Aspergillus flavipes*. Bioorg Med Chem. 2024;103:117685. 38503009 10.1016/j.bmc.2024.117685

[23] Metzger U, Schall C, Zocher G, Unsöld I, Stec E, Li S-M, et al. The structure of dimethylallyl tryptophan synthase reveals a common architecture of aromatic prenyltransferases in fungi and bacteria. Proc Natl Acad Sci. 2009;106:14309–14. 19706516 10.1073/pnas.0904897106 PMC2732893

[24] Chiang Y-M, Oakley CE, Ahuja M, Entwistle R, Schultz A, Chang S-L, et al. An efficient system for heterologous expression of secondary metabolite genes in *Aspergillus nidulans*. J Am Chem Soc. 2013;135:7720–31. 23621425 10.1021/ja401945a PMC3697937

[25] Frisch M, Trucks G, Schlegel H, Scuseria G, Robb M, Cheeseman J, et al. Gaussian 09. Gaussian Inc, Wallingford, CT. 2009;32:5648–52.

[26] Turakhia Y, Chen HI, Marcovitz A, Bejerano G. A fully-automated method discovers loss of mouse-lethal and human-monogenic disease genes in 58 mammals. Nucleic Acids Res. 2020;48:e91. 32614390 10.1093/nar/gkaa550 PMC7498332

[27] Sánchez-Serna G, Badia-Ramentol J, Bujosa P, Ferrández-Roldán A, Torres-Águila NP, Fabregà-Torrus M, et al. Less, but more: new insights from appendicularians on chordate fgf evolution and the divergence of tunicate lifestyles. Mol Biol Evol. 2025;42:msae260. 39686543 10.1093/molbev/msae260 PMC11733497

[28] Yin W-B, Cheng J, Li S-M. Stereospecific synthesis of aszonalenins by using two recombinant prenyltransferases. Org Biomol Chem. 2009;7:2202–7. 19421461 10.1039/b902413a

[29] Kelly SP, Shende VV, Flynn AR, Dan Q, Ye Y, Smith JL, et al. Data science-driven analysis of substrate-permissive diketopiperazine reverse prenyltransferase NotF: applications in protein engineering and cascade biocatalytic synthesis of (−)-eurotiumin A. J Am Chem Soc. 2022;144:19326–36. 36223664 10.1021/jacs.2c06631 PMC9831672

[30] Jost M, Zocher G, Tarcz S, Matuschek M, Xie X, Li S-M, et al. Structure−function analysis of an enzymatic prenyl transfer reaction identifies a reaction chamber with modifiable specificity. J Am Chem Soc. 2010;132:17849–58. 21105662 10.1021/ja106817c

[31] Cui CB, Kakeya H, Osada H. Novel mammalian cell cycle inhibitors, tryprostatins A, B and other diketopiperazines produced by *Aspergillus fumigatus*. II. Physico-chemical properties and structures. J Antibiot (Tokyo). 1996;49:534–40. 8698635 10.7164/antibiotics.49.534

[32] Rabindran SK, Ross DD, Doyle LA, Yang W, Greenberger LM. Fumitremorgin C reverses multidrug resistance in cells transfected with the breast cancer resistance protein. Cancer Res. 2000;60:47–50. 10646850

[33] Li P, Meng J, Zhang X, Zhang X, Ye Y, Zhao Y, et al. Cooperative redox reactions encoded by two gene clusters enable intermolecular cycloaddition cascade for the formation of meroaspochalasins. Angew Chem Int Ed. 2025;64:e202502766. 10.1002/anie.202502766 40129378

[34] Pfeifer BA, Admiraal SJ, Gramajo H, Cane DE, Khosla C. Biosynthesis of complex polyketides in a metabolically engineered strain of *E. coli*. Science. 2001;291:1790–2. 11230695 10.1126/science.1058092

