# Supplementary material for: Discovery of unprecedented prenylated indole piperazines and pyrazines through cryptic biosynthetic gene cluster heterologous expression
Source: Nat Prod Bioprospect. 2026 Apr 3;16(1):49. doi: 10.1007/s13659-026-00601-7 (PMC13047030; doi:10.1007/s13659-026-00601-7)
Supplement: Supplementary file 1 — Additional file 1. [file 13659_2026_601_MOESM1_ESM.docx]

**Discovery of Unprecedented Prenylated Indole Piperazines and Pyrazines through Cryptic Biosynthetic Gene Cluster Heterologous Expression**

Ziou Zha,^a,1^ Dan He,^a,1^ Jianguo Song,^b,1^ Zhenhua Guan^c^, Jiapei Han^a^, Chang Liu^a^, Xinyu Wang^a^, Yongchun Zhu^a^, Hucheng Zhu^a^, Wencai Ye,^b^ Qin Li,^a,^* Yonghui Zhang,^a,^* Yuan Zhou^a,^*

*^a^Hubei Key Laboratory of Natural Medicinal Chemistry and Resource Evaluation, School of Pharmacy, Tongji Medical College, Huazhong University of Science and Technology, Wuhan 430030,* *Hubei Province, People’s Republic of China*

*^b^State Key Laboratory of Bioactive Molecules and Druggability Assessment, Jinan University, Guangzhou 510632, Guangdong Province, People’s Republic of China*

*^c^Department of Pharmacy, Shenzhen People's Hospital (The Second Clinical Medical College, Jinan University, The First Affiliated Hospital), Southern University of Science and Technology, Shenzhen, 518055, Guangdong Province, People’s Republic of China*

*Corresponding Author: [liqin2023@hust.edu.cn](mailto:liqin2023@hust.edu.cn). (Q.L.) zhangyh@mails.tjmu.edu.cn. (Y-H.Z.); zhouyuan@hust.edu.cn (Y.Z.)

^1^These authors contributed equally.

**1 Supplementary Tables** 7

Table S1. Strains or plasmids used in this study 7

Table S2. The primers used in this study 8

Table S3. Bioinformatics analysis of the *flz* gene cluster in this study 10

Table S4. NMR data of compound 1 in CD_3_OD 11

Table S5. NMR data of compound 2 in CD_3_OD 12

Table S6. NMR data of compound 3 in DMSO-*d*_6_ 13

Table S7. NMR data of compound 4 in CD_3_OD 14

Table S8. NMR data of compound 5 in CD_3_OD. 15

Table S9. NMR data of compound 6 in DMSO-*d*_6_ 16

Table S10. NMR data of compound 7 in CD_3_OD 17

Table S11. NMR data of compound 10 in CD_3_OD. 18

Table S12. NMR data of compound 11 in CD_3_OD 20

Table S13. NMR data of compound 12 in CD_3_OD 22

Table S14. NMR data of compound 13 in CD_3_OD. 24

Table S15. NMR data of compound 14 in DMSO-*d*_6_. 25

Table S16. NMR data of compound 15 in DMSO-*d*_6_. 26

Table S17. NMR data of compound 16 in DMSO-*d*_6_. 28

Table S18. NMR data of compound 17 in CD_3_OD. 30

**2 Supplementary Figures** 32

Figure S1. All the reported gene clusters for the biosynthesis of piperazine in fungi. 32

Figure S2. The reactions catalyzed by DMATSs in the phylogenetic tree. 33

Figure S3. SDS-PAGE analysis of the purified FlzE. 34

Figure S4. Sequence alignment of reported DMATS that catalyze tryptophan or indole-containing compounds and FlzE. 36

Figure S6. UV spectrum of compound 1 37

Figure S7. IR spectrum of compound 1 37

Figure S8. Crystal structure of compound 1 38

Figure S9. ^1^H NMR spectrum of compound 1 in CD_3_OD (600 MHz) 38

Figure S10. ^13^C NMR spectrum of compound 1 in CD_3_OD (150 MHz) 39

Figure S11. HSQC spectrum of compound 1 in CD_3_OD (600 MHz) 40

Figure S12. HMBC spectrum of compound 1 in CD_3_OD (600 MHz) 40

Figure S13. ^1^H-^1^H COSY spectrum of compound 1 in CD_3_OD (600 MHz) 41

Figure S14. HRESIMS spectrum of compound 2 42

Figure S15. UV spectrum of compound 2 42

Figure S16. IR spectrum of compound 2 42

Figure S17. ^1^H NMR spectrum of compound 2 in CD_3_OD (600 MHz) 43

Figure S18. ^13^C NMR spectrum of compound 2 in CD_3_OD (150 MHz) 44

Figure S19. HSQC spectrum of compound 2 in CD_3_OD (600 MHz) 45

Figure S20. HMBC spectrum of compound 2 in CD_3_OD (600 MHz) 45

Figure S21. ^1^H-^1^H COSY spectrum of compound 2 in CD_3_OD (600 MHz) 46

Figure S22. ^1^H NMR spectrum of compound 3 in DMSO-*d*_6_ (600 MHz) 47

Figure S24. HSQC spectrum of compound 3 in DMSO-*d*_6_ (600 MHz) 49

Figure S25. HMBC spectrum of compound 3 in DMSO-*d*_6_ (600 MHz) 49

Figure S26. ^1^H-^1^H COSY spectrum of compound 3 in DMSO-*d*_6_ (600 MHz) 50

Figure S27. HRESIMS spectrum of compound 4 51

Figure S28. UV spectrum of compound 4 51

Figure S29. IR spectrum of compound 4 51

Figure S30. ^1^H NMR spectrum of compound 4 in CD_3_OD (600 MHz) 52

Figure S31. ^13^C NMR spectrum of compound 4 in CD_3_OD (150 MHz) 53

Figure S32. HSQC spectrum of compound 4 in CD_3_OD (600 MHz) 54

Figure S33. HMBC spectrum of compound 4 in CD_3_OD (600 MHz) 54

Figure S34. ^1^H-^1^H COSY spectrum of compound 4 in CD_3_OD (600 MHz) 55

Figure S35. HRESIMS spectrum of compound 5 56

Figure S36. UV spectrum of compound 5 56

Figure S37. IR spectrum of compound 5 56

Figure S38. Crystal structure of compound 5 57

Figure S39. ^1^H NMR spectrum of compound 5 in CD_3_OD (600 MHz) 57

Figure S40. ^13^C NMR spectrum of compound 5 in CD_3_OD (150 MHz) 58

Figure S41. HSQC spectrum of compound 5 in CD_3_OD (600 MHz) 59

Figure S42. HMBC spectrum of compound 5 in CD_3_OD (600 MHz) 59

Figure S43. ^1^H-^1^H COSY spectrum of compound 5 in CD_3_OD (600 MHz) 60

Figure S44. HRESIMS spectrum of compound 6 61

Figure S45. UV spectrum of compound 6 61

Figure S46. IR spectrum of compound 6 61

Figure S47. ^1^H NMR spectrum of compound 6 in DMSO-*d*_6_(600 MHz) 62

Figure S48. ^13^C NMR spectrum of compound 6 in DMSO-*d*_6_ (150 MHz) 63

Figure S49. HSQC spectrum of compound 6 in DMSO-*d*_6_ (600 MHz) 64

Figure S50. HMBC spectrum of compound 6 in DMSO-*d*_6_ (600 MHz) 64

Figure S51. ^1^H-^1^H COSY spectrum of compound 6 in DMSO-*d*_6_ (600 MHz) 65

Figure S52. ^1^H NMR spectrum of compound 7 in CD_3_OD (600 MHz) 66

Figure S53. ^13^C NMR spectrum of compound 7 in CD_3_OD (150 MHz) 67

Figure S54. HSQC spectrum of compound 7 in CD_3_OD (600 MHz) 68

Figure S55. HMBC spectrum of compound 7 in CD_3_OD (600 MHz) 68

Figure S56. ^1^H-^1^H COSY spectrum of compound 7 in CD_3_OD (600 MHz) 69

Figure S57. HRESIMS spectrum of compound 8 70

Figure S58. UV spectrum of compound 8 70

Figure S59. IR spectrum of compound 8 70

Figure S60. ^1^H NMR spectrum of compound 8 in CD_3_OD (600 MHz) 71

Figure S61. ^13^C NMR spectrum of compound 8 in CD_3_OD (150 MHz) 72

Figure S62. HSQC spectrum of compound 8 in CD_3_OD (600 MHz) 73

Figure S63. HMBC spectrum of compound 8 in CD_3_OD (600 MHz) 73

Figure S64. ^1^H-^1^H COSY spectrum of compound 8 in CD_3_OD (600 MHz) 74

Figure S65. NOESY spectrum of compound 8 in CD_3_OD (600 MHz) 74

Figure S66. HRESIMS spectrum of compound 9 75

Figure S67. UV spectrum of compound 9 75

Figure S68. IR spectrum of compound 9 75

Figure S69. ^1^H NMR spectrum of compound 9 in CD_3_OD (600 MHz) 76

Figure S70. ^13^C NMR spectrum of compound 9 in CD_3_OD (150 MHz) 77

Figure S71. HSQC spectrum of compound 9 in CD_3_OD (600 MHz) 78

Figure S72. HMBC spectrum of compound 9 in CD_3_OD (600 MHz) 78

Figure S73. ^1^H-^1^H COSY spectrum of compound 9 in CD_3_OD (600 MHz) 79

Figure S74. NOESY spectrum of compound 9 in CD_3_OD (600 MHz) 79

Figure S75. HRESIMS spectrum of compound 10 80

Figure S76. UV spectrum of compound 10 80

Figure S77. IR spectrum of compound 10 80

Figure S78. ^1^H NMR spectrum of compound 10 in CD_3_OD (600 MHz) 81

Figure S79. ^13^C NMR spectrum of compound 10 in CD_3_OD (150 MHz) 82

Figure S80. HSQC spectrum of compound 10 in CD_3_OD (600 MHz) 83

Figure S81. HMBC spectrum of compound 10 in CD_3_OD (600 MHz) 83

Figure S82. ^1^H-^1^H COSY spectrum of compound 10 in CD_3_OD (600 MHz) 84

Figure S83. HRESIMS spectrum of compound 11 85

Figure S84. UV spectrum of compound 11 85

Figure S85. IR spectrum of compound 11 85

Figure S86. ^1^H NMR spectrum of compound 11 in CD_3_OD (600 MHz) 86

Figure S87. ^13^C NMR spectrum of compound 11 in CD_3_OD (150 MHz) 87

Figure S88. HSQC spectrum of compound 11 in CD_3_OD (600 MHz) 88

Figure S89. HMBC spectrum of compound 11 in CD_3_OD (600 MHz) 88

Figure S90. ^1^H-^1^H COSY spectrum of compound 11 in CD_3_OD (600 MHz) 89

Figure S91. HRESIMS spectrum of compound 12 90

Figure S92. UV spectrum of compound 12 90

Figure S93. IR spectrum of compound 12 90

Figure S94. ^1^H NMR spectrum of compound 12 in CD_3_OD (600 MHz) 91

Figure S95. ^13^C NMR spectrum of compound 12 in CD_3_OD (150 MHz) 92

Figure S96. HSQC spectrum of compound 12 in CD_3_OD (600 MHz) 93

Figure S97. HMBC spectrum of compound 12 in CD_3_OD (600 MHz) 93

Figure S98. ^1^H-^1^H COSY spectrum of compound 12 in CD_3_OD (600 MHz) 94

Figure S99. HRESIMS spectrum of compound 13 95

Figure S100. UV spectrum of compound 13 95

Figure S101. IR spectrum of compound 13 95

Figure S102. ^1^H NMR spectrum of compound 13 in CD_3_OD (600 MHz) 96

Figure S103. ^13^C NMR spectrum of compound 13 in CD_3_OD (150 MHz) 97

Figure S104. HSQC spectrum of compound 13 in CD_3_OD (600 MHz) 98

Figure S105. HMBC spectrum of compound 13 in CD_3_OD (600 MHz) 98

Figure S106. ^1^H-^1^H COSY spectrum of compound 13 in CD_3_OD (600 MHz) 99

Figure S107. ^1^H NMR spectrum of compound 14 in DMSO-*d*_6_ (600 MHz) 100

Figure S108. ^1^H NMR spectrum of compound 15 in DMSO-*d*_6_ (600 MHz) 101

Figure S109. ^13^C NMR spectrum of compound 15 in DMSO-*d*_6_ (150 MHz) 101

Figure S110. HSQC spectrum of compound 15 in DMSO-*d*_6_ (600 MHz) 102

Figure S111. HMBC spectrum of compound 15 in DMSO-*d*_6_ (600 MHz) 102

Figure S112. ^1^H-^1^H COSY spectrum of compound 15 in DMSO-*d*_6_ (600 MHz) 103

Figure S113. ^1^H NMR spectrum of compound 16 in DMSO-*d*_6_ (600 MHz) 104

Figure S114. ^13^C NMR spectrum of compound 16 in DMSO-*d*_6_ (150 MHz) 104

Figure S115. HRESIMS spectrum of compound 17 105

Figure S116. UV spectrum of compound 17 105

Figure S117. IR spectrum of compound 17 105

Figure S118. ^1^H NMR spectrum of compound 17 in CD_3_OD (600 MHz) 106

Figure S119. ^13^C NMR spectrum of compound 17 in CD_3_OD (150 MHz) 107

Figure S120. HSQC spectrum of compound 17 in CD_3_OD (600 MHz) 108

Figure S121. HMBC spectrum of compound 17 in CD_3_OD (600 MHz) 108

Figure S122. ^1^H-^1^H COSY spectrum of compound 17 in CD_3_OD (600 MHz) 109

Figure S123. NOESY spectrum of compound 17 in CD_3_OD (600 MHz) 109

Figure S124. Experimental and calculated ECD spectra of 17 in MeOH 110

References 111

**1 Supplementary Tables**

**Table S1.** Strains or plasmids used in this study

| Strains or plasmids | Description |
| --- | --- |
| *Aspergillus flavipes* | Wild-type strain |
| *Aspergillus nidulans* LO8030 | Host for heterologous expression |
| *Saccharomyces cerevisiae* BJ5464-NpgA | Host for heterologous recombination to construct the *A. nidulans* overexpression plasmids |
| *Escherichia coli* DH5α | Host for plasmids propagation |
| *Escherichia coli* BL21 | Host for protein expression |
| *Escherichia coli* BAP1 | Host for NRPS expression |
| pYTU | *E. coli*-*Saccharomyces*-*A. nidulans* shuttle vector for heterologous expression, ampicillin resistance, Ura3 and pyrG89 |
| pYTR | *E. coli*-*Saccharomyces*-*A. nidulans* shuttle vector for heterologous expression, ampicillin resistance, Ura3 and riboB2 |
| pYTP | *E. coli*-*Saccharomyces*-*A. nidulans* shuttle vector for heterologous expression, ampicillin resistance, Ura3 and pyroA4 |
| pET-28a | protein expression vector used in *E. coli* with T7 promoter, 6xHis tag, T7 terminator, kanamycin resistance |

**Table S2.** The primers used in this study

| Primer name | Primer sequence (5′→3′) |
| --- | --- |
| *glaA*-F | CCTGATCTTCCGAACTGGTC |
| *glaA*-R | TGCTGAGGTGTAATGATGCT |
| *gpdA*-F | ACTCCGGTGAATTGATTTGG |
| *gpdA*-R | TGTTTAGATGTGTCTATGTGGC |
| *amyB*-F | GATTAAAGGTGCCGAACGAG |
| *amyB*-R | AAATGCCTTCTGTGGGGTTT |
| pYTR-flzA-F | TAGACACATCTAAACATTAATTAAGGATCCATGACTGTATCTTCCCGTGC |
| pYTR-flzA-R | CTAAAGGGTATCATCGAAAGGGAGTCATCCAATTTAAATCAAAACTGACACAT |
| pYTU-flzB-F | TTACACCTCAGCATTAATTAAGGCGGCCGCATGACTCGCCCTCCAGTCAC |
| pYTU-flzB-R | CATACCCGTAATTTTCTGGGCATTTAAATAGTACAGGAGTCCTTGTTTGC |
| pYTP-flzE-F | CCCACAGAAGGCATTTTTAATTAAGGATCCATGACCATCGAAGGAAATAA |
| pYTP-flzE-R | CCAACAACCATGATACCAGGGGATTTAAATATGTTAATTCTGTGTTCATAG |
| pYTP-flzC-F | CCCACAGAAGGCATTTTTAATTAAGGATCCATGGCAGTGAGCTTCGAATC |
| pYTP-flzC-R | CTCCCGTCACCCAAATCAATTCACCGGAGTCTGTGTTTGTCCTGTTTGTA |
| pYTP-flzD-F | ATTACCCCGCCACATAGACACATCTAAACAATGGATATACTCGGAAAGAC |
| pYTP-flzD-R | CCAACAACCATGATACCAGGGGATTTAAATAAATTTGGCTTGTGAGATAT |

| pYTP-flzE-R-1 | CTCCCGTCACCCAAATCAATTCACCGGAGTATGTTAATTCTGTGTTCATAG |
| --- | --- |
| pYTP-flzF-F | ATTACCCCGCCACATAGACACATCTAAACAATGCCACCGGGTTTTCACTC |
| pYTP-flzF-R | CCAACAACCATGATACCAGGGGATTTAAATACAGGTGCTTTACAGACCTC |
| pYTU-flzB-R-1 | CCAAATCAATTCACCGGAGTAAATAGTACAGGAGTCCTTGTT |
| pYTU-flzA-F | CCATTACCCCGCCACATAGACACATCTAAACAATGACTGTATCTTCCCGT |
| pYTU-flzA-R | CACAGTGGAGGACATACCCGTAATTTTCTGGGCATTTCAAAACTGACACA |
| pYTR-flzC-F | TAGACACATCTAAACATTAATTAAGGATCCATGGCAGTGAGCTTCGAATC |
| pYTR-flzC-R | TCATTTATAGCTCGTTCGGCACCTTTAATCCTGTGTTTGTCCTGTTTGTA |
| pYTR-flzD-F | ACAATAAACCCCACAGAAGGCATTTATGGATATACTCGGAAAGAC |
| pYTR-flzD-R | CGAAAGGGAGTCATCCAATTTAAATTTGGCTTGTGAGATATCCGT |
| pET-28a-FlzA-F | AGCAAATGGGTCGCGGATCCATGACTGTATCTTCCCGTGC |
| pET-28a-FlzA-R | GTGGTGGTGGTGGTGCTCGATCAGATCAAGTCTACACTAGC |
| pET-28a-FlzE-F | GTGGACAGCAAATGGGTCGCATGACCATCGAAGGAAATAAAC |
| pET-28a-FlzE-R | CAGTGGTGGTGGTGGTGGTGTTCCTTTCTAAGCGAGGGAT |

**Table S3.** Bioinformatics analysis of the *flz* gene cluster in this study

| Gene name | Size (gene, bp/protein, aa) | BLASTP homologs^a^ | Identity/positives (%) | Putative function |
| --- | --- | --- | --- | --- |
| *flzA* | 3132/1043 | CpsA | 70/83 | NRPS-like protein (A-T-R) |
| *flzB* | 1185/334 | CpsB | 62/77 | short-chain dehydrogenase/reductase (SDR) |
| *flzC* | 1837/504 | CpsC | 28/46 | cytochrome P450 |
| *flzD* | 1578/525 | DrtD | 39/57 | cytochrome P450 |
| *flzE* | 1325/422 | OkaC | 40/59 | aromatic prenyltransferases |
| *flzF* | 1001/223 | CpsF | 46/62 | methyltransferase |

^a^If available, the closest characterized homolog is displayed; otherwise, the locus tag of the closest BLASTP homolog is shown.

**Table S4**. NMR data of compound **1** in CD_3_OD (^1^H NMR in 600 MHz, ^13^C NMR in 125 MHz).


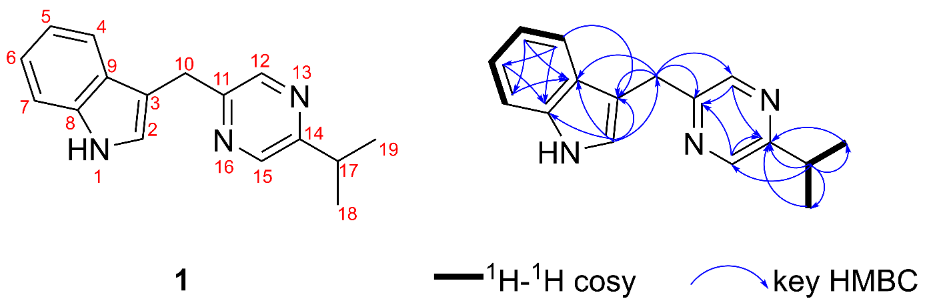


| **No.** | ***δ*_H_ (mult, *J* in Hz)** | ***δ*_C_, type** | **HMBC** | **COSY** |
| --- | --- | --- | --- | --- |
| 2 | 7.12 (s) | 124.3, CH | C3, C8, C9, C10 |  |
| 3 |  | 112.7, C |  |  |
| 4 | 7.42 (d, 8.0) | 119.3, CH_2_ | C3, C6, C8, C9 | H5 |
| 5 | 6.96 (t, 7.6) | 119.9, CH_2_ | C7, C9 | H4, H6 |
| 6 | 7.08 (t, 7.5) | 122.6, CH_2_ | C4, C8 | H5, H7 |
| 7 | 7.34 (d, 8.2) | 112.4, CH_2_ | C5, C9 | H6 |
| 8 |  | 138.3, C |  |  |
| 9 |  | 128.3, C |  |  |
| 10 | 4.27 (s) | 32.2, CH_2_ | C2, C3, C9, C11, C12 |  |
| 11 |  | 156.0, C |  |  |
| 12 | 8.39 (d, 1.4) | 144.7, CH | C11, C14 |  |
| 14 |  | 160.8, C |  |  |
| 15 | 8.42 (d, 1.5) | 142.6, CH | C11, C14 |  |
| 17 | 3.08 (m) | 34.6, CH_2_ | C14, C15, C18, C19 | H18, H19 |
| 18 | 1.29 (d, 7.0) | 22.5, CH_3_ | C14, C17 | H17 |
| 19 | 1.28 (d, 7.0) | 22.5, CH_3_ | C14, C17 | H17 |

**Table S5**. NMR data of compound **2** in CD_3_OD (^1^H NMR in 600 MHz, ^13^C NMR in 125 MHz).


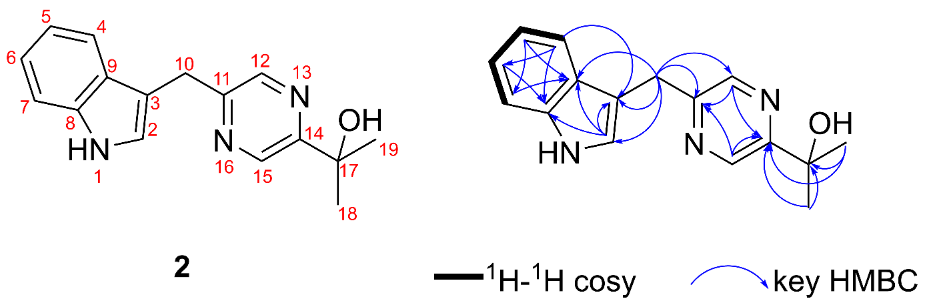


| **No.** | ***δ*_H_ (mult, *J* in Hz)** | ***δ*_C_, type** | **HMBC** | **COSY** |
| --- | --- | --- | --- | --- |
| 2 | 7.12 (s) | 124.3, CH | C3, C8, C9 |  |
| 3 |  | 112.7, C |  |  |
| 4 | 7.42 (d, 8.0) | 119.3, CH_2_ | C3, C6, C8 | H5 |
| 5 | 6.96 (t, 7.6) | 119.9, CH_2_ | C7, C9 | H4, H6 |
| 6 | 7.08 (t, 7.5) | 122.6, CH_2_ | C4, C8 | H5, H7 |
| 7 | 7.34 (d, 8.2) | 112.4, CH_2_ | C5, C9 | H6 |
| 8 |  | 138.3, C |  |  |
| 9 |  | 128.3, C |  |  |
| 10 | 4.29 (s) | 32.2, CH_2_ | C2, C3, C9, C11, C12 |  |
| 11 |  | 156.1, C |  |  |
| 12 | 8.42 (d, 1.5) | 144.1, CH | C11, C14 |  |
| 14 |  | 161.4, C |  |  |
| 15 | 8.77 (d, 1.5) | 140.6, CH | C11, C14 |  |
| 17 |  | 73.4, C |  |  |
| 18 | 1.53 (s) | 30.4, CH_3_ | C14, C17 |  |
| 19 | 1.53 (s) | 30.4, CH_3_ | C14, C17 |  |

**Table S6.** NMR data of compound **3** in DMSO-*d*_6_ (^1^H NMR in 600 MHz, ^13^C NMR in 125 MHz).

**
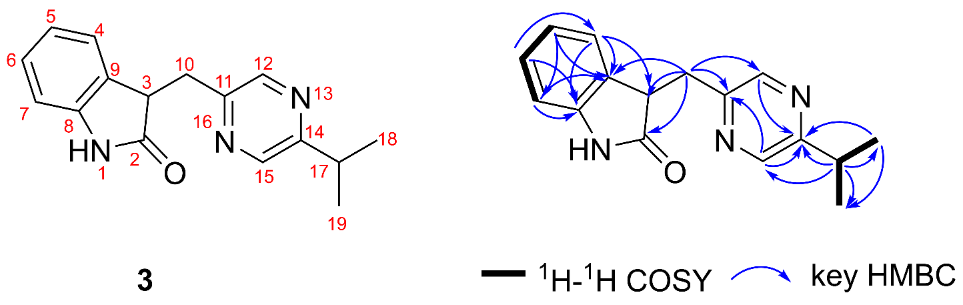
**

| **No.** | ***δ*_H_ (mult, *J* in Hz)** | ***δ*_C_, type** | **HMBC** | **COSY** |
| --- | --- | --- | --- | --- |
| 2 |  | 181.4, C |  |  |
| 3 | 3.94 (m) | 46.6, CH |  |  |
| 4 | 6.87 (d, 6.8) | 125.3, CH | C3, C8, C9 |  |
| 5 | 6.89 (t, 7.6) | 123.1, CH | C7. C9 | H6 |
| 6 | 7.14 (t, 7,4) | 129.2, CH | C4, C8 | H5, H7 |
| 7 | 6.83 (d, 7.7) | 110.8, CH | C5, C9 | H6 |
| 8 |  | 143.6, C |  |  |
| 9 |  | 130.2, C |  |  |
| 10 | 3.27 (d, 14.6)  3.47 (d, 14.6) | 35.9, CH_2_ | C2, C3, C9, C11, C12 |  |
| 11 |  | 152.6, C |  |  |
| 12 | 8.33 (d, 1.6) | 144.9, CH | C11, C14 |  |
| 14 |  | 161.4, C |  |  |
| 15 | 8.41 (d, 1.6) | 143.3, CH | C11, C14 |  |
| 17 | 3.08 (m) | 34.7, CH | C14, C15, C18, C19 | H18, H19 |
| 18 | 1.29 (d, 6.9) | 22.5, CH_3_ | C14, C17, C19 | H17 |
| 19 | 1.29 (d, 6.9) | 22.5, CH_3_ | C14, C17, C18 | H17 |

**Table S7**. NMR data of compound **4** in CD_3_OD (^1^H NMR in 600 MHz, ^13^C NMR in 125 MHz).


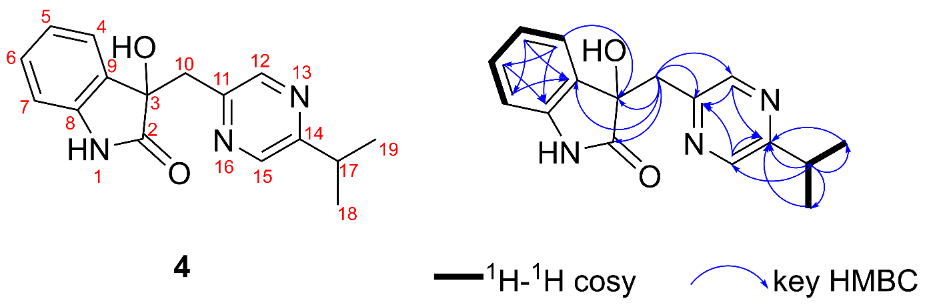


| **No.** | ***δ*_H_ (mult, *J* in Hz)** | ***δ*_C_, type** | **HMBC** | **COSY** |
| --- | --- | --- | --- | --- |
| 2 |  | 181.1, C |  |  |
| 3 |  | 77.5, C |  |  |
| 4 | 7.09 (d, 6.7) | 125.7, CH_2_ | C3, C6, C8 | H5 |
| 5 | 6.96 (t, 7.6) | 123.4, CH_2_ | C7, C9 | H4, H6 |
| 6 | 7.17 (t, 7.7) | 130.7, CH_2_ | C4, C8 | H5, H7 |
| 7 | 6.73 (d, 7.8) | 111.1, CH_2_ | C5, C9 | H6 |
| 8 |  | 142.5, C |  |  |
| 9 |  | 131.6, C |  |  |
| 10 | 3.39 (s) | 43.7, CH_2_ | C2, C3, C9, C11, C12 |  |
|  | 3.35 (s) |  |  |  |
| 11 |  | 150.3, C |  |  |
| 12 | 8.28 (d, 1.5) | 145.8, CH | C11, C14 |  |
| 14 |  | 161.4, C |  |  |
| 15 | 8.28 (d, 1.5) | 142.6, CH | C11, C14 |  |
| 17 | 3.02 (m) | 34.6, CH | C14, C15, C18, C19 | H18, H19 |
| 18 | 1.25 (d, 7.0) | 22.4, CH_3_ | C14, C17 | H17 |
| 19 | 1.25 (d, 7.0) | 22.4, CH_3_ | C14, C17 | H17 |

**Table S8**. NMR data of compound **5** in CD_3_OD (^1^H NMR in 600 MHz, ^13^C NMR in 125 MHz).


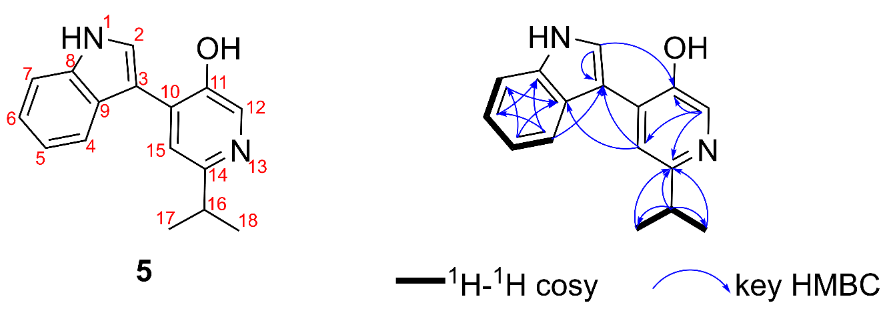


| **No.** | ***δ*_H_ (mult, *J* in Hz)** | ***δ*_C_, type** | **HMBC** | **COSY** |
| --- | --- | --- | --- | --- |
| 2 | 7.75 (s) | 120.8, CH | C3, C11 |  |
| 3 |  | 110.5, C |  |  |
| 4 | 7.91 (d, 7.8) | 120.6, CH | C3, C6, C8 | H5 |
| 5 | 7.18 (t, 7.4) | 121.6, CH | C7, C9 | H4, H6 |
| 6 | 7.22 (t, 7.4) | 123.2, CH | C4, C8 | H5, H7 |
| 7 | 7.48 (d, 7.9) | 113.1, CH | C5, C9 | H6 |
| 8 |  | 138.2, C |  |  |
| 9 |  | 127.0, C |  |  |
| 10 |  | 136.6, C |  |  |
| 11 |  | 151.0, C |  |  |
| 12 | 8.02 (s) | 133.9, CH | C11, C14 |  |
| 14 |  | 157.3, C |  |  |
| 15 | 8.09 (s) | 130.2, CH | C3, C9, C12 |  |
| 16 | 3.09 (m) | 35.6, CH | C14, C17, C18 |  |
| 17 | 1.37 (d, 6.8) | 23.0, CH_3_ | C14, C16 | H16 |
| 18 | 1.38 (d, 6.8) | 23.0, CH_3_ | C14, C16 | H16 |

**Table S9**. NMR data of compound **6** in DMSO-*d*_6_ (^1^H NMR in 600 MHz, ^13^C NMR in 125 MHz).


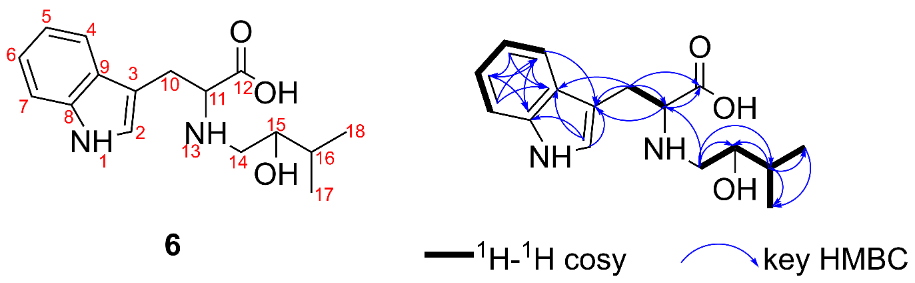


| **No.** | ***δ*_H_ (mult, *J* in Hz)** | ***δ*_C_, type** | **HMBC** | **COSY** |
| --- | --- | --- | --- | --- |
| 2 | 7.23 (d, 2.3) | 123.9, CH | C3, C8, C9 |  |
| 3 |  | 109.6, C |  |  |
| 4 | 7.58 (d, 7.8) | 118.5, CH | C3, C6, C8, C9 | H5 |
| 5 | 6.98 (t, 6.9) | 118.4, CH | C6, C9 | H4, H6 |
| 6 | 7.06 (t, 6.9) | 121.0, CH | C4, C8 | H5, H7 |
| 7 | 7.34 (d, 8.1) | 111.3, CH | C4, C9 | H6 |
| 8 |  | 136.2, C |  |  |
| 9 |  | 127.1, C |  |  |
| 10 | 3.28 (dd, 15.1, 5.1)  3.09 (dd, 15.1, 7.8) | 26.4, CH_2_ | C3, C9, C11, C12 |  |
| 11 | 3.54 (dd, 7.8, 5.1) | 61.9, CH | C3, C10, C12, C14 | H10 |
| 12 |  | 170.5, C |  |  |
| 14 | 2.74 (dd,12.2, 2.0)  2.50 (overlapped) | 50.0, CH_2_ | C11, C15 | H15 |
| 15 | 3.31 (m) | 71.2, CH |  | H14, H16 |
| 16 | 1.48 (m) | 31.5, CH | C14, C15 | H15, H17, H18 |
| 17 | 0.74 (d, 6.7) | 18.3, CH_3_ | C15, C16, C18 | H16 |
| 18 | 0.68 (d, 6.8) | 17.6, CH_3_ | C15, C16, C17 | H16 |

**Table S10**. NMR data of compound **7** in CD_3_OD (^1^H NMR in 600 MHz, ^13^C NMR in 125 MHz).


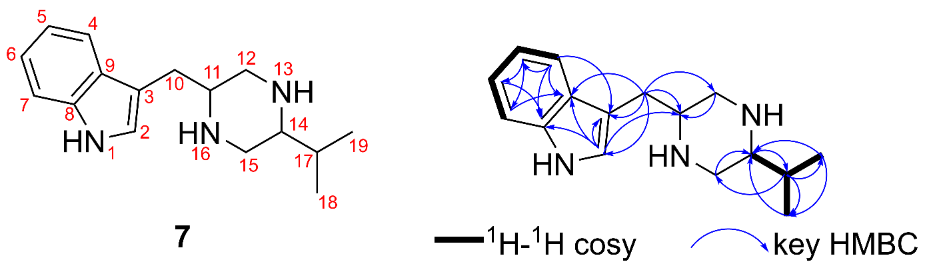


| **No.** | ***δ*_H_ (mult, *J* in Hz)** | ***δ*_C_, type** | **HMBC** | **COSY** |
| --- | --- | --- | --- | --- |
| 2 | 7.17 (s) | 124.9, CH | C3, C8, C9, C10, C11 |  |
| 3 |  | 109.3, C |  |  |
| 4 | 7.58 (d, 7.9) | 119.1, CH | C3, C6, C7, C8, C9 | H5 |
| 5 | 7.05 (t, 7.4) | 120.1, CH | C4, C6, C7, C8, C9 | H4, H6 |
| 6 | 7.13 (t, 7.5) | 122.8, CH | C4, C7, C8, C9 | H7 |
| 7 | 7.37 (d, 8.1) | 112.6, CH | C5, C9 |  |
| 8 |  | 138.3, C |  |  |
| 9 |  | 128.4, C |  |  |
| 10 | 3.03 (dd, 14.9, 6.7)  2.99 (dd, 14.8, 7.2) | 28.9, CH_2_ | C2, C3, C9, C11, C12 |  |
| 11 | 3.33 (overlapped) | 55.8, CH |  |  |
| 12 | 2.81 (m) | 49.8, CH_2_ | C11 |  |
| 14 | 2.78 (m) | 60.7, CH | C15, C17, C18, C19 |  |
| 15 | 2.72 (m) | 47.6, CH_2_ | C14 |  |
| 17 | 1.75 (m) | 31.3, CH | C14, C15, C18, C19 | H14, H18, H19 |
| 18 | 1.01 (d, 6.8) | 18.9, CH_3_ | C14, C17, C19 | H17 |
| 19 | 0.98 (d, 6.8) | 18.6, CH_3_ | C14, C17, C18 | H17 |

**Table S11**. NMR data of compound **10** in CD_3_OD (^1^H NMR in 600 MHz, ^13^C NMR in 125 MHz).


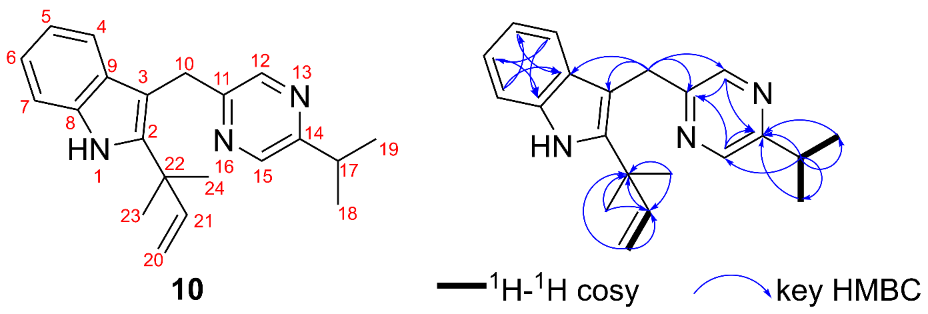


| **No.** | ***δ*_H_ (mult, *J* in Hz)** | ***δ*_C_, type** | **HMBC** | **COSY** |
| --- | --- | --- | --- | --- |
| 2 |  | 143.3, C |  |  |
| 3 |  | 106.5, C |  |  |
| 4 | 7.16 (d, 7.9) | 118.4, CH | C6, C8 |  |
| 5 | 7.05 (t, 7.1) | 122.1, CH | C9 |  |
| 6 | 6.92 (t, 7.0) | 120.0, CH | C4, C8 |  |
| 7 | 7.38 (d, 8.1) | 112.0, CH | C5, C9 |  |
| 8 |  | 136.5, C |  |  |
| 9 |  | 130.5, C |  |  |
| 10 | 4.39 (s) | 31.8, CH_2_ | C3, C9, C11, C12 |  |
| 11 |  | 156.3, C |  |  |
| 12 | 8.01 (d,1.4) | 144.2, CH | C11, C14 |  |
| 14 |  | 160.5, C |  |  |
| 15 | 8.43 (d,1.5) | 142.5, CH | C11, C14 |  |
| 17 | 3.08 (m) | 34.5, CH | C14, C15, C18, C19 | H18, H19 |
| 18 | 1.28 (d, 6.9) | 22.5, CH_3_ | C14, C17 | H17 |
| 19 | 1.28 (d, 6.9) | 22.5, CH_3_ | C14, C17 | H17 |
| 20 | 4.99 (overlapped) | 111.9, CH_2_ | C21, C22 | H21 |
| 21 | 6.12 (dd, 17.4, 10.5) | 147.3, CH | C22 | H20 |
| 22 |  | 40.0, C |  |  |
| 23 | 1.50 (s) | 28.3, CH_3_ | C21, C22 |  |
| 24 | 1.50 (s) | 28.3, CH_3_ | C21, C22 |  |

**Table S12**. NMR data of compound **11** in CD_3_OD (^1^H NMR in 600 MHz, ^13^C NMR in 125 MHz).


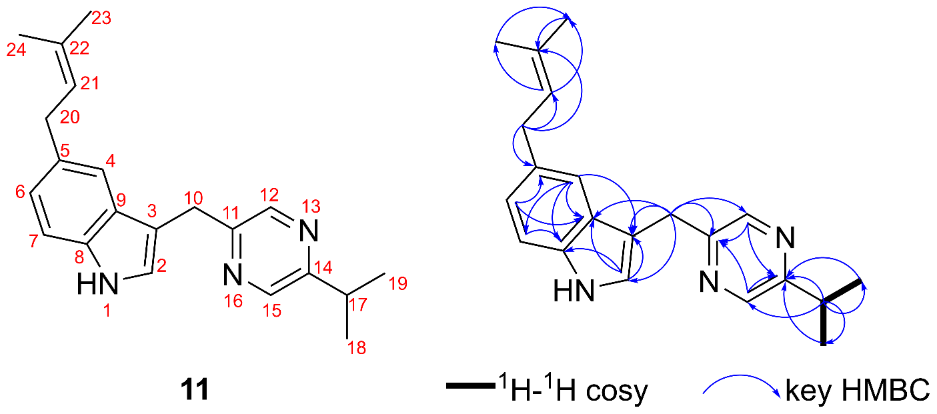


| **No.** | ***δ*_H_ (mult, *J* in Hz)** | ***δ*_C_, type** | **HMBC** | **COSY** |
| --- | --- | --- | --- | --- |
| 2 | 7.09 (s) | 124.4, CH | C3, C8, C9 |  |
| 3 |  | 112.2, C |  |  |
| 4 | 7.16 (d, 1.7) | 118.3, CH | C3, C7, C8, C9 |  |
| 5 |  | 133.3, C |  |  |
| 6 | 7.24 (d, 8.3) | 112.4, CH | C5, C9 |  |
| 7 | 6.92 (dd, 8.3, 1.8) | 123.6, CH | C4, C8 |  |
| 8 |  | 136.8, C |  |  |
| 9 |  | 128.5, C |  |  |
| 10 | 4.25 (s) | 32.3, CH_2_ | C2, C3, C9, C11, C12 |  |
| 11 |  | 156.01, C |  |  |
| 12 | 8.38 (s) | 144.8, CH | C11, C14 |  |
| 14 |  | 166.7, C |  |  |
| 15 | 8.42 (s) | 142.6, CH | C11, C14 |  |
| 17 | 3.09 (m) | 34.8, CH_2_ | C14, C15, C18, C19 | H18, H19 |
| 18 | 1.30 (d, 6.9) | 22.5, CH_3_ | C14, C17, C19 | H17 |
| 19 | 1.30 (d, 6.9) | 22.5, CH_3_ | C14, C17, C18 | H17 |
| 20 | 3.34 (d, 6.7) | 35.5, CH_2_ | C5, C21, C22 |  |
| 21 | 5.30 (t, 7.4) | 125.8, CH | C23, C24 |  |
| 22 |  | 132.2, C |  |  |
| 23 | 1.71 (s) | 17.8, CH_3_ | C21, C22, C24 |  |
| 24 | 1.71 (s) | 25.8, CH_3_ | C23 |  |

**Table S13**. NMR data of compound **12** in CD_3_OD (^1^H NMR in 600 MHz, ^13^C NMR in 125 MHz).


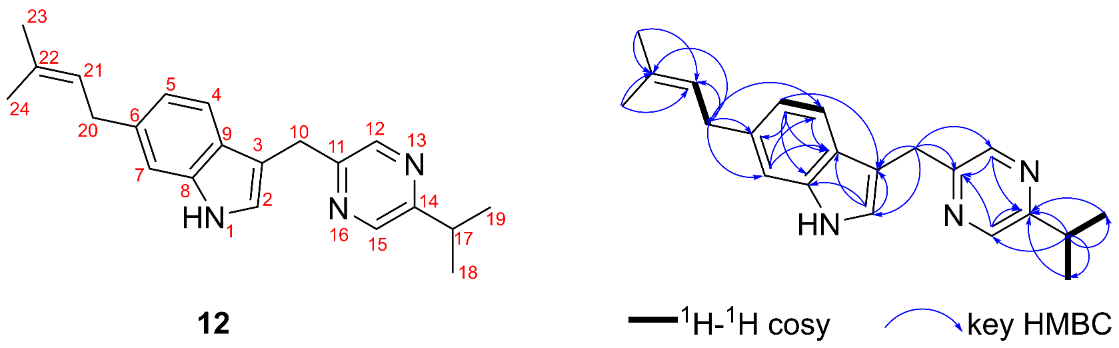


| **No.** | ***δ*_H_ (mult, *J* in Hz)** | ***δ*_C_, type** | **HMBC** | **COSY** |
| --- | --- | --- | --- | --- |
| 2 | 7.05 (s) | 123.8, CH | C3, C8, C9, C10 |  |
| 3 |  | 112.5, C |  |  |
| 4 | 6.80 (d, 8.2) | 121.0, CH | C7, C9, C20 | H5 |
| 5 | 7.30 (d, 8.1) | 119.1, CH | C3, C6, C8, C9 | H4 |
| 6 |  | 136.6, C |  |  |
| 7 | 7.13 (s) | 115.6, CH | C4, C9, C20 |  |
| 8 |  | 138.7, C |  |  |
| 9 |  | 126.5, C |  |  |
| 10 | 4.24 (s) | 32.3, CH_2_ | C3, C9, C11, C12 |  |
| 11 |  | 156.0, C |  |  |
| 12 | 8.38 (s) | 144.7, CH | C11, C14 |  |
| 14 |  | 160.7, C |  |  |
| 15 | 8.41 (s) | 142.6, CH | C11, C14 |  |
| 17 | 3.08 (m) | 34.5, CH_2_ | C14, C15, C18, C19 | H18, H19 |
| 18 | 1.29 (d, 6.9) | 22.5, CH_3_ | C14, C17 | H17 |
| 19 | 1.29 (d, 6.9) | 22.5, CH_3_ | C14, C17 | H17 |
| 20 | 3.39 (d, 7.4) | 35.5, CH_2_ | C6, C7, C21, C22 | H21 |
| 21 | 5.34 (m) | 125.5, CH |  | H20 |
| 22 |  | 132.5, C |  |  |
| 23 | 1.73 (s) | 17.5, CH_3_ | C21, C22 |  |
| 24 | 1.74 (s) | 25.9, CH_3_ | C21, C22 |  |

**Table S14**. NMR data of compound **13** in CD_3_OD (^1^H NMR in 600 MHz, ^13^C NMR in 125 MHz).


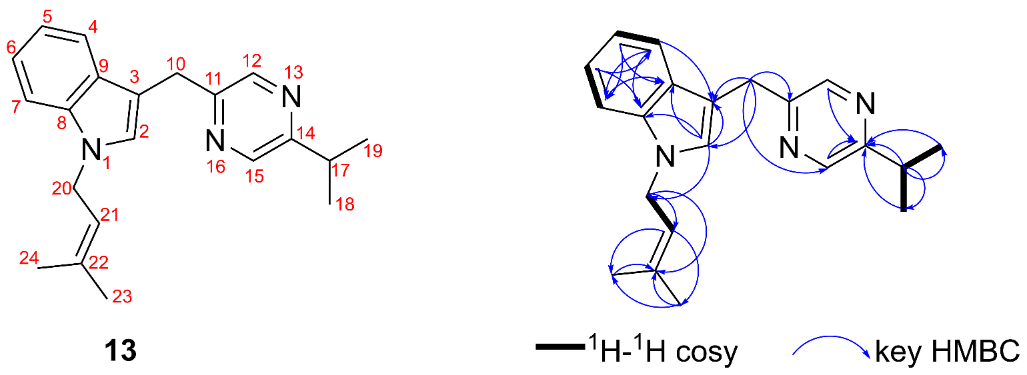


| **No.** | **δ_H_ (mult, *J* in Hz)** | **δ_C_, type** | **HMBC** | **COSY** |
| --- | --- | --- | --- | --- |
| 2 | 7.08 (s) | 127.3, CH | C3, C8, C9 |  |
| 3 |  | 112.3, C |  |  |
| 4 | 7.44 (d, 7.9) | 119.7, CH | C3, C6, C8 | H5 |
| 5 | 6.99 (t, 7.5) | 120.0, CH | C7, C9 | H4 |
| 6 | 7.12 (t, 7.6) | 122.6, CH | C4, C8 | H7 |
| 7 | 7.31(d, 8.2) | 110.7, CH | C5, C9 | H6 |
| 8 |  | 138.0, C |  |  |
| 9 |  | 129.1, C |  |  |
| 10 | 4.27(s) | 32.1, CH_2_ | C2, C3, C11, C15 |  |
| 11 |  | 155.9, C |  |  |
| 12 | 8.40 (s) | 144.8, CH | C14 |  |
| 14 |  | 160.4, C |  |  |
| 15 | 8.42 (s) | 142.7, CH | C14 |  |
| 17 | 3.08 (m) | 34.6, CH | C14, C18, C19 | H18, H19 |
| 18 | 1.29 (d, 6.9） | 22.5, CH_3_ | C14, C17 | H17 |
| 19 | 1.29 (d, 6.9) | 22.5, CH_3_ | C14, C17 | H17 |
| 20 | 4.71 (d, 6.8) | 44.8, CH_2_ | C2, C21, C22 | H21 |
| 21 | 5.35 (t, 6.8) | 121.5, CH | C23, C24 | H20 |
| 22 |  | 137.0, C |  |  |
| 23 | 1.84 (s) | 18.0, CH_3_ | C21, C22, C24 |  |
| 24 | 1.75 (s) | 25.8, CH_3_ | C21, C22, C23 |  |

**Table S15.** NMR data of compound **14** in DMSO-*d*_6_ (^1^H NMR in 600 MHz, ^13^C NMR in 125 MHz).

**
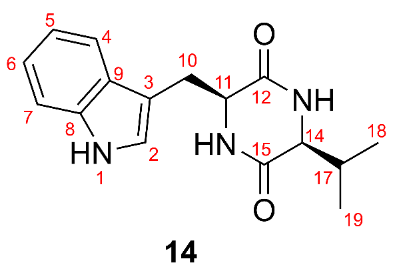
**

|  | **this work (in DMSO-*d*_6_)** | **reported (in DMSO-*d*_6_)**^[1]^ |
| --- | --- | --- |
| **No** | ***δ*_H_ (mult., *J* in Hz)** | ***δ*_H_ (mult., *J* in Hz)** |
| 1 | 10.83 (s, 1H) | 10.87 (s, 1H) |
| 2 | 7.07 (d, 2.1, 1H) | 7.07 (d, 2.3, 1H) |
| 3 |  |  |
| 4 | 7.59 (d, 7.9, 1H) | 7.59 (d, 7.9, 1H) |
| 5 | 6.93 (t, 7.3, 1H) | 6.93 (t, 7.4, 1H) |
| 6 | 7.01 (t, 7.3, 1H) | 7.01 (t, 7.5, 1H) |
| 7 | 7.28 (d, 8.1, 1H) | 7.28 (d, 8.0, 1H) |
| 8 |  |  |
| 9 |  |  |
| 10 | 3.06 (dd, 14.4, 5.0, 1H)  3.20 (dd, 14.4, 4.5, 1H) | 3.07 (dd, 14.5, 4.6, 1H)  3.21 (dd, 14.4, 5.0, 1H) |
| 11 | 4.13 (t, 4.4, 1H) | 4.16-4.11 (m, 1H) |
| 12 |  |  |
| 13 | 7.97 (s, 1H) | 8.00 (s, 1H) |
| 14 | 3.48 (m, 1H) | 3.48 (dt, 3.9, 1.8,1H) |
| 15 |  |  |
| 16 | 7.86 (s, 1H) | 7.88 (d, 2.2, 1H) |
| 17 | 1.63 (m, 1H) | 1.64 (dtd, 14.0, 7.1, 3.8,1H) |
| 18 | 0.60 (d, 7.1, 3H) | 0.59 (d, 7.1, 3H) |
| 19 | 0.16 (d, 6.8, 3H) | 0.16 (d, 6.8, 3H) |

**Table S16.** NMR data of compound **15** in DMSO-*d*_6_ (^1^H NMR in 600 MHz, ^13^C NMR in 125 MHz).

**
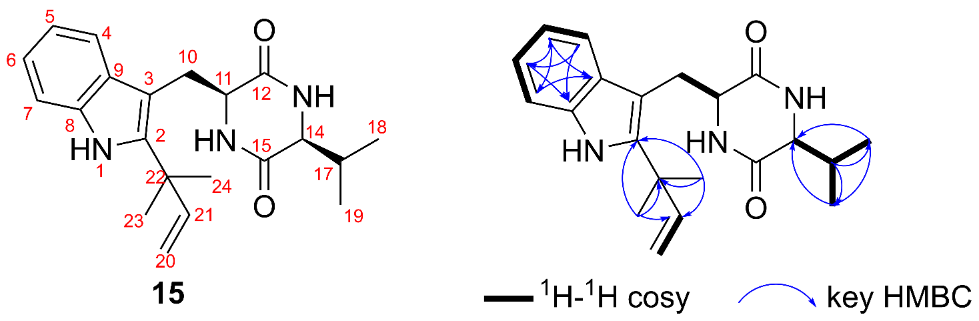
**

| **No.** | ***δ*_H_ (mult, *J* in Hz)** | ***δ*_C_, type** | **HMBC** | **COSY** |
| --- | --- | --- | --- | --- |
| 2 |  | 141.6, C |  |  |
| 3 |  | 105.1, C |  |  |
| 4 | 7.43 (d, 8.1) | 117.6, CH | C6, C8 | H5 |
| 5 | 6.92 (m) | 118.6, CH | C7. C9 | H4, H6 |
| 6 | 7.01 (m) | 120.7, CH | C5, C8 | H5, H7 |
| 7 | 7.32 (d, 8.0) | 111.2, CH | C5, C9 | H6 |
| 8 |  | 135.1, C |  |  |
| 9 |  | 129.2, C |  |  |
| 10 | 3.41 (dd, 14.5, 4.3)  3.01 (dd, 14.5, 9.2) | 31.6, CH_2_ |  | H11 |
| 11 | 4.01 (m) | 55.9, CH |  | H10 |
| 12 |  | 166.7, C |  |  |
| 14 | 3.49 (overlapped) | 60.2, CH |  |  |
| 15 |  | 166.9, C |  |  |
| 17 | 1.98 (m) | 32.6, CH | C18, C19 | H18, H19 |
| 18 | 0.97 (d, 7.0) | 19.2, CH_3_ | C14, C17, C19 | H17 |
| 19 | 0.90 (d, 7.0) | 13.7, CH_3_ | C14, C17, C18 | H17 |
| 20 | 5.01 (m) | 110.0, CH_2_ |  | H21 |
| 21 | 6.15 (m) | 146.7, CH | C23, C24 | H20 |
| 22 |  | 38.5, C |  |  |
| 23 | 1.48 (s) | 23.2, CH_3_ | C2, C21, C22 |  |
| 24 | 1.48 (s) | 28.2, CH_3_ | C2, C21, C22 |  |

**Table S17.** NMR data of compound **16** in DMSO-*d*_6_ (^1^H NMR in 600 MHz, ^13^C NMR in 125 MHz).

**
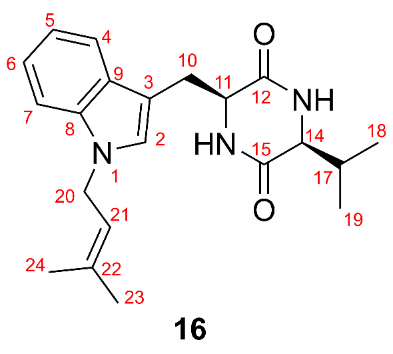
**

|  | **this work (in DMSO-*d*_6_)** | | **reported (in CDCl_3_)**^[2]^ | |
| --- | --- | --- | --- | --- |
| **No** | ***δ*_H_ (mult., *J* in Hz)** | ***δ*_C_, type** | ***δ*_H_ (mult., *J* in Hz)** | ***δ*_C_, type** |
| 2 | 7.03 (s) | 127.5, CH | 7.01 (s) | 126.8, CH |
| 3 |  | 108.4, C |  | 108.1, C |
| 4 | 7.60 (d, 7.9) | 118.5, CH | 7.61 (d, 7.8) | 118.9, CH |
| 5 | 6.95 (t, 7.0) | 120.6, CH | 7.14 (dd, 7.8, 6.9) | 119.8, CH |
| 6 | 7.09 (t, 6.8) | 120.9, CH | 7.24 (dd, 8.0, 6.9) | 122.3, CH |
| 7 | 7.29 (d, 8.2) | 109.5, CH | 7.34 (d, 8.0) | 110.1, CH |
| 8 |  | 135.2, C |  | 136.7, C |
| 9 |  | 128.7, C |  | 127.6, C |
| 10 | 3.20 (dd, 14.5, 10.4)  3.02 (dd, 14.7, 4.7) | 31.3, CH_2_ | 3.00 (dd, 14.5, 10.5)  3.70 (dd, 14.5, 3.2) | 31.1, CH_2_ |
| 11 | 4.13 (m) | 55.3, CH | 4.30 (m) | 55.3, CH |
| 12 |  | 166.4, C |  | 166.5, C |
| 13 | 8.04 (s) |  | 5.87 (s) |  |
| 14 | 3.45 (overlapped) | 59.4, CH | 3.90 (m) | 60.5, CH |
| 15 |  | 166.4, C |  | 167.8, C |
| 16 | 7.86 (s) |  | 5.83 (s) |  |
| 17 | 2.38 (m) | 32.8, CH | 2.35 (m) | 31.5, CH |
| 18 | 0.92 (d, 7.3) | 13.6, CH_3_ | 0.90 (d, 6.6) | 16.4, CH_3_ |
| 19 | 1.30 (d, 7.3) | 13.6, CH_3_ | 1.05 (d, 7.1) | 19.1, CH_3_ |
| 20 | 4.65 (d, 6.9) | 43.4, CH_2_ | 4.67 (d, 6.8) | 44.4, CH_2_ |
| 21 | 5.26 (m) | 119.4, CH | 5.38 (t, 6.8) | 119.8, CH |
| 22 |  | 135.7, C |  | 136.7, C |
| 23 | 1.79 (s) | 19.3, CH_3_ | 1.85 (s) | 18.4, CH_3_ |
| 24 | 1.69 (s) | 23.2, CH_3_ | 1.80 (s) | 26.0, CH_3_ |

**Table S18.** NMR data of compound **17** in CD_3_OD (^1^H NMR in 600 MHz, ^13^C NMR in 125 MHz).

**
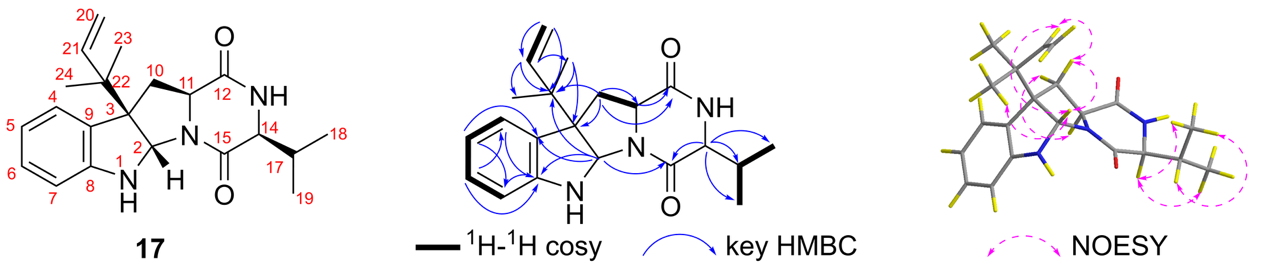
**

| **No.** | ***δ*_H_ (mult, *J* in Hz)** | ***δ*_C_, type** | **COSY** | **HMBC** |
| --- | --- | --- | --- | --- |
| 2 | 5.59 (s) | 78.4, CH |  | C8, C9, C15, C20 |
| 3 |  | 62.9, C |  |  |
| 4 | 7.19 (d, 7.5) | 126.1, CH | H5 | C6, C8 |
| 5 | 6.71 (t, 6.9) | 119.5, CH | H4, H6 | C7. C9 |
| 6 | 7.05 (t, 7.1) | 130.0, CH | H5, H7 | C4, C8 |
| 7 | 6.58 (d, 7.8) | 110.8, CH | H6 | C5, C9 |
| 8 |  | 152.4, C |  |  |
| 9 |  | 130.3, C |  |  |
| 10 | 2.47 (dd,12.2, 5.0)  2.32 (t, 12.2) | 38.9, CH_2_ | H11 | C3, C9, C11, C12, C20 |
| 11 | 3.91 (m) | 59.3, CH | H10 | C10, C12 |
| 12 |  | 171.4, C |  |  |
| 14 | 3.97 (m) | 61.6, CH |  | C15, C17, C18 |
| 15 |  | 166.9, C |  |  |
| 17 | 2.45 (m) | 31.1, CH | H18, H19 | C14, C18, C19 |
| 18 | 0.91 (d, 6.8) | 16.6, CH_3_ | H17 | C14, C17, C19 |
| 19 | 1.29 (d, 6.9) | 18.7, CH_3_ | H17 | C14, C17, C18 |
| 20 | 5.12 (d, 10.8)  5.09 (d, 17.4) | 114.8, CH_2_ | H21 | C21, C22 |
| 21 | 6.02 (dd, 17.3, 10.9) | 145.1, CH | H20 | C22, C23, C24 |
| 22 |  | 42.2, C |  |  |
| 23 | 0.98 (s) | 23.3, CH_3_ |  | C3, C21, C22, C24 |
| 24 | 1.13 (s) | 22.9, CH_3_ |  | C3, C21, C22, C23 |

**2 Supplementary Figures**


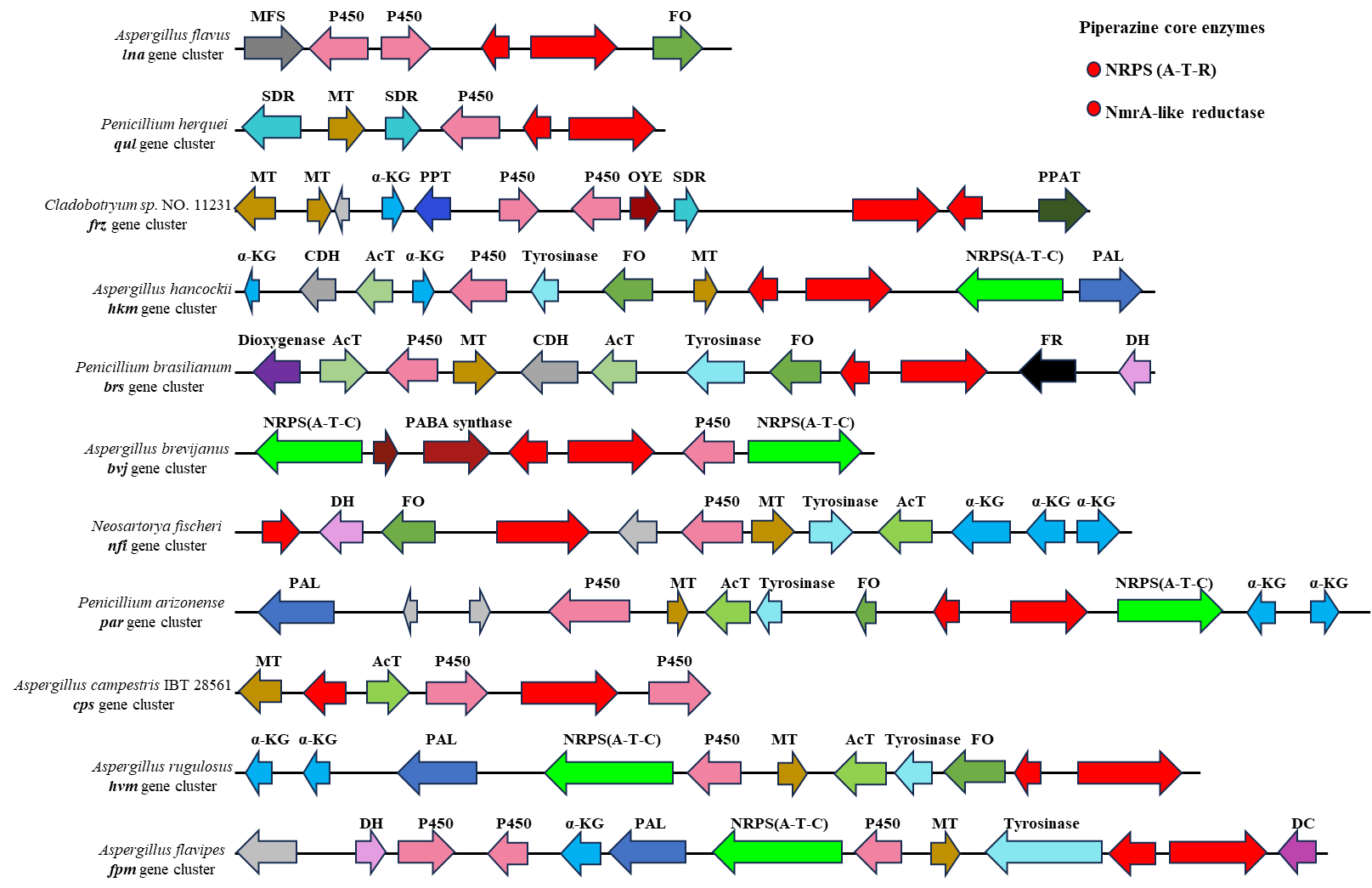


**Figure S1.** All the reported gene clusters for the biosynthesis of piperazine in fungi.^[3]^

**
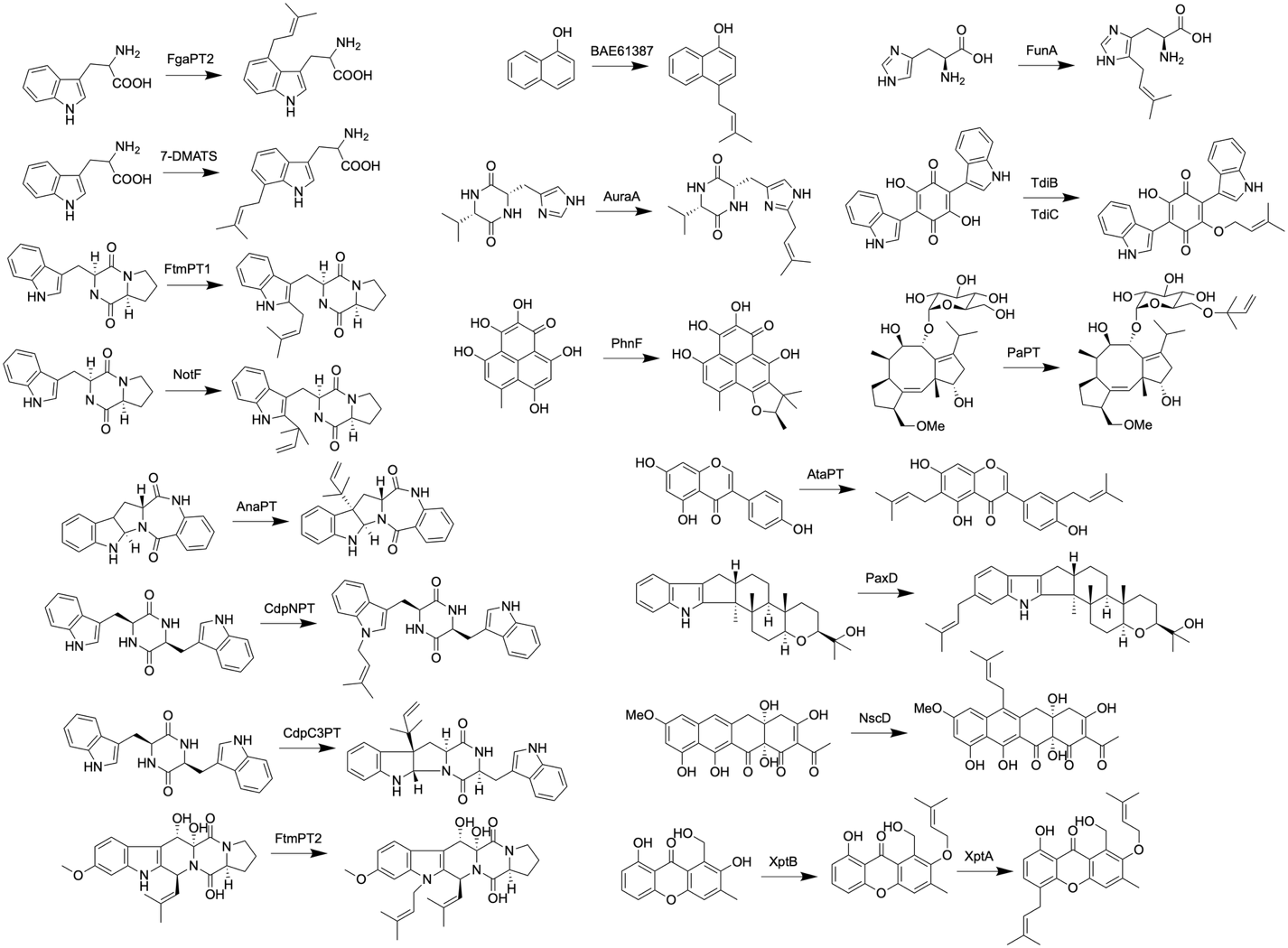
**

**Figure S2.** The reactions catalyzed by DMATSs in the phylogenetic tree (Figure 4a).

**
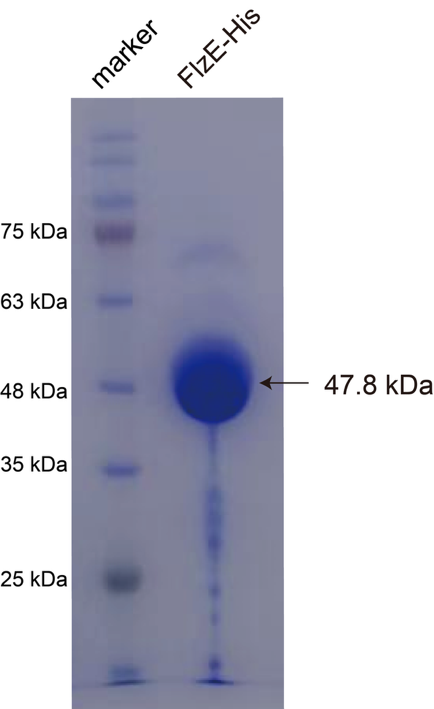
**

**Figure S3.** SDS-PAGE analysis of the purified FlzE.

**
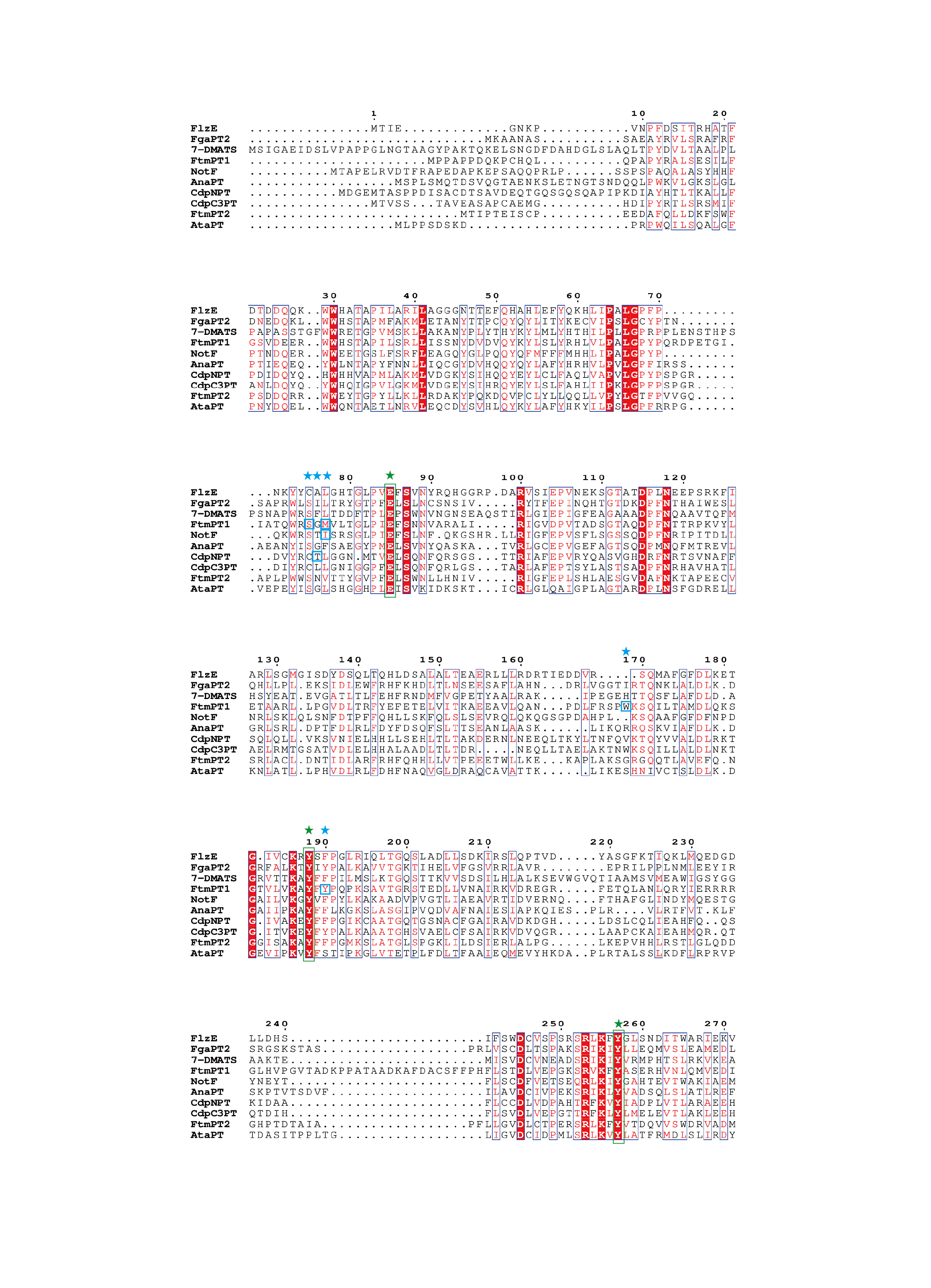
**

**
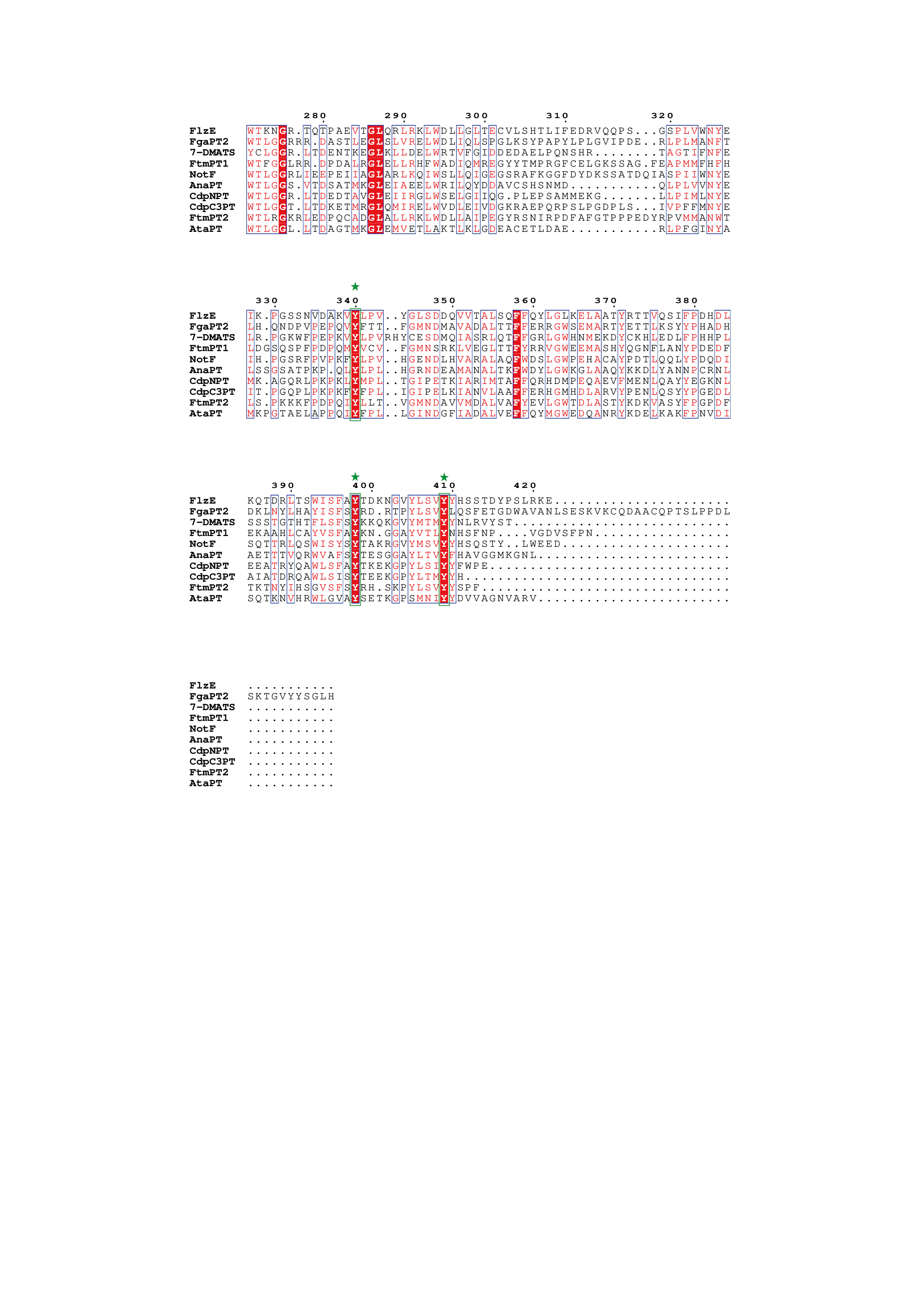
**

**Figure S4.** Sequence alignment of reported DMATS that catalyze tryptophan or indole-containing compounds and FlzE. Conserved tyrosine residues and 81E in FlzE were marked in green box and star. Non-conserved residues were marked in blue box and star.


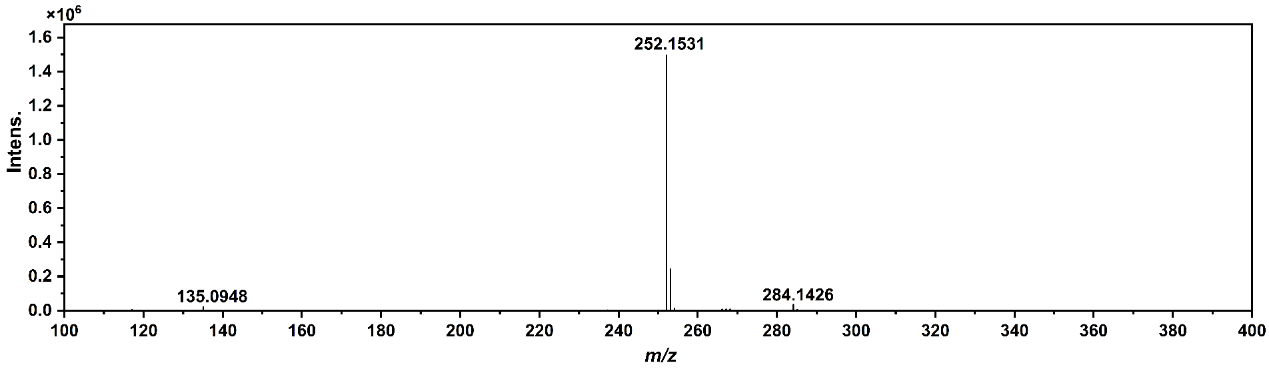
 **Figure S5**. HRESIMS spectrum of compound **1**


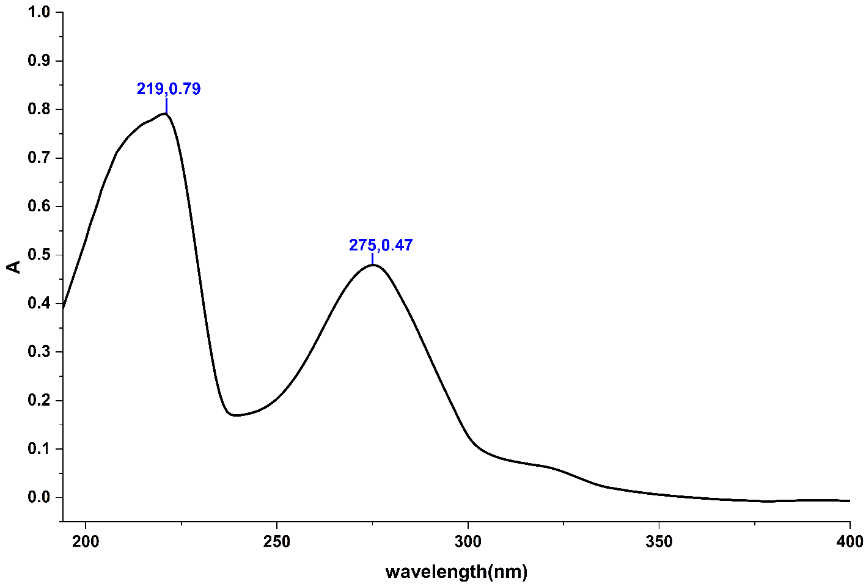


**Figure S6**. UV spectrum of compound **1**

**
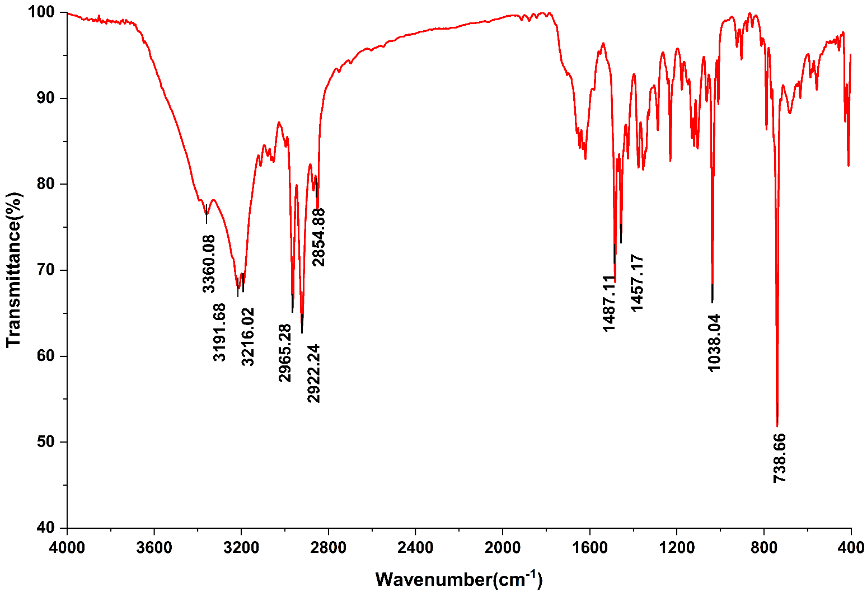
**

**Figure S7**. IR spectrum of compound **1**


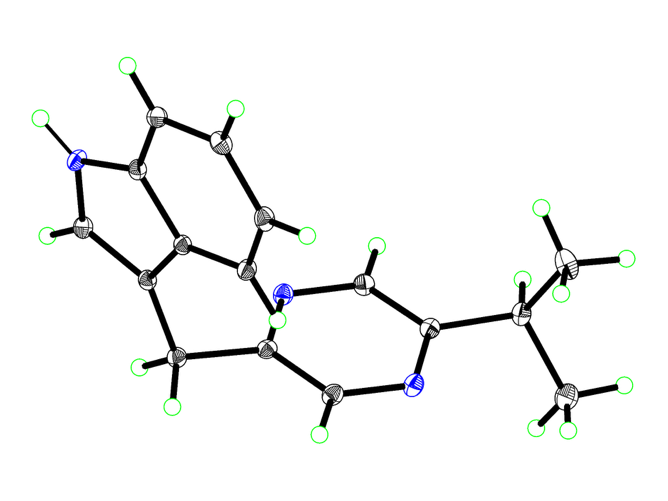


**Figure S8**. Crystal structure of compound **1**

**Figure S9.** ^1^H NMR spectrum of compound **1** in CD_3_OD (600 MHz)

**Figure S10.** ^13^C NMR spectrum of compound **1** in CD_3_OD (150 MHz)

**Figure S11.** HSQC spectrum of compound **1** in CD_3_OD (600 MHz)

**Figure S12.** HMBC spectrum of compound **1** in CD_3_OD (600 MHz)

**Figure S13**. ^1^H-^1^H COSY spectrum of compound **1** in CD_3_OD (600 MHz)


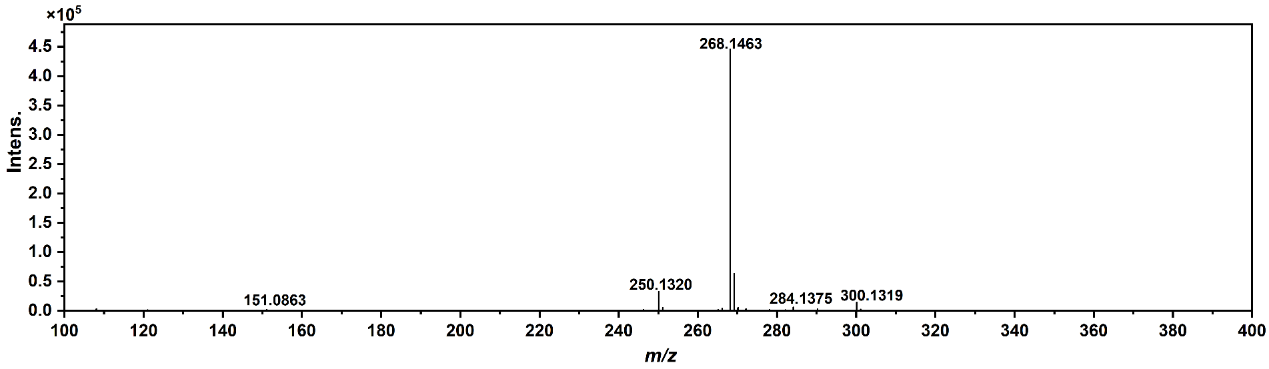


**Figure S14**. HRESIMS spectrum of compound **2**


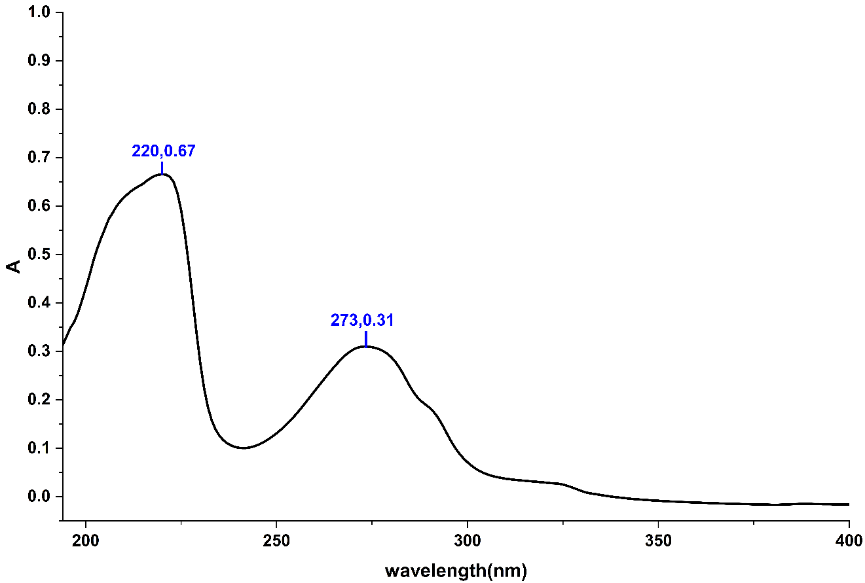


**Figure S15**. UV spectrum of compound **2**

**
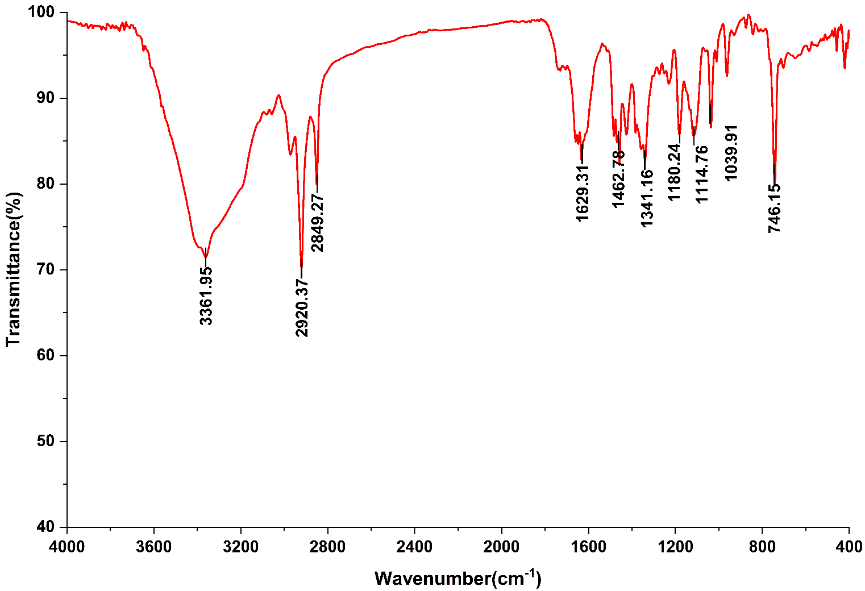
**

**Figure S16**. IR spectrum of compound **2**

**Figure S17.** ^1^H NMR spectrum of compound **2** in CD_3_OD (600 MHz)

**Figure S18.** ^13^C NMR spectrum of compound **2** in CD_3_OD (150 MHz)

**Figure S19.** HSQC spectrum of compound **2** in CD_3_OD (600 MHz)

**Figure S20.** HMBC spectrum of compound **2** in CD_3_OD (600 MHz)

**Figure S21**. ^1^H-^1^H COSY spectrum of compound **2** in CD_3_OD (600 MHz)


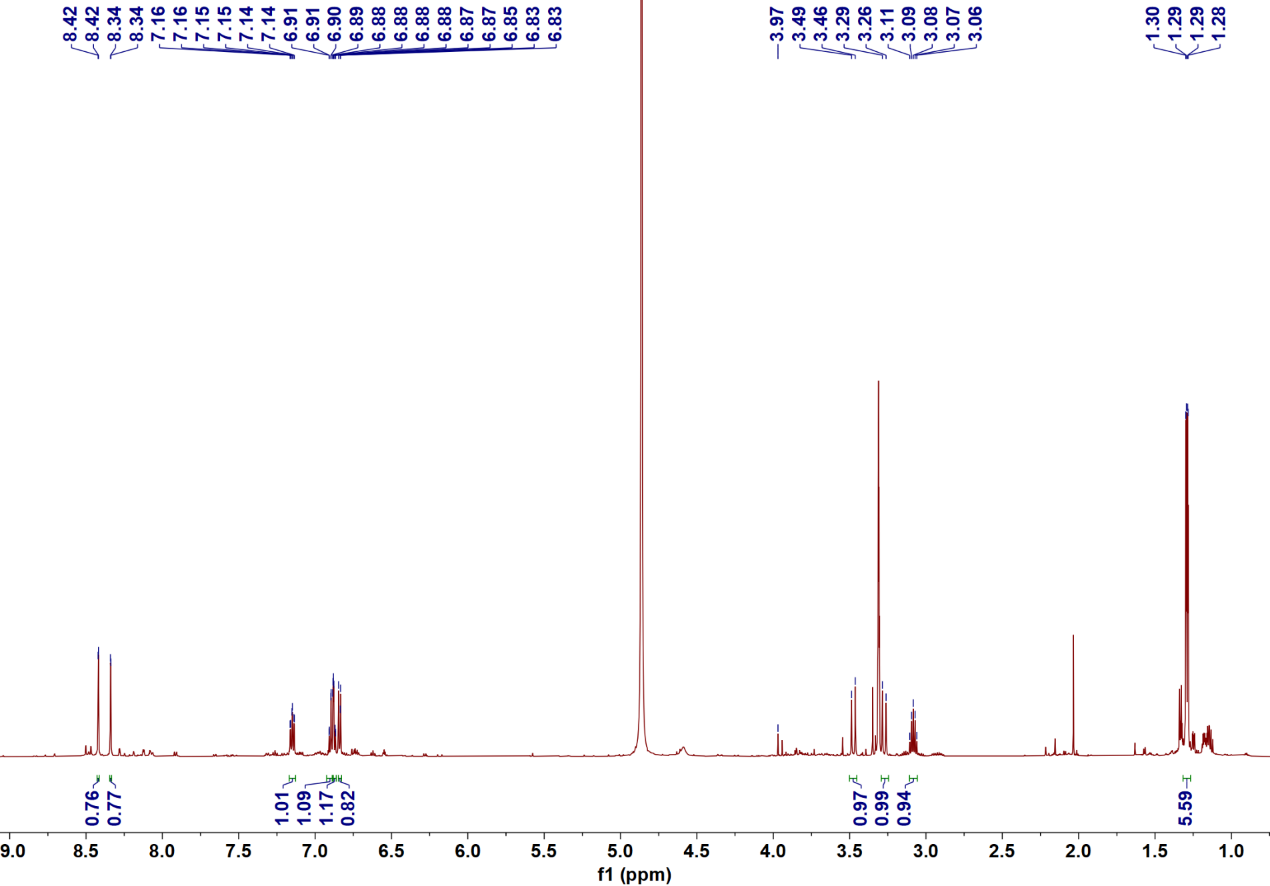


**Figure S22.** ^1^H NMR spectrum of compound **3** in DMSO-*d*_6_ (600 MHz)


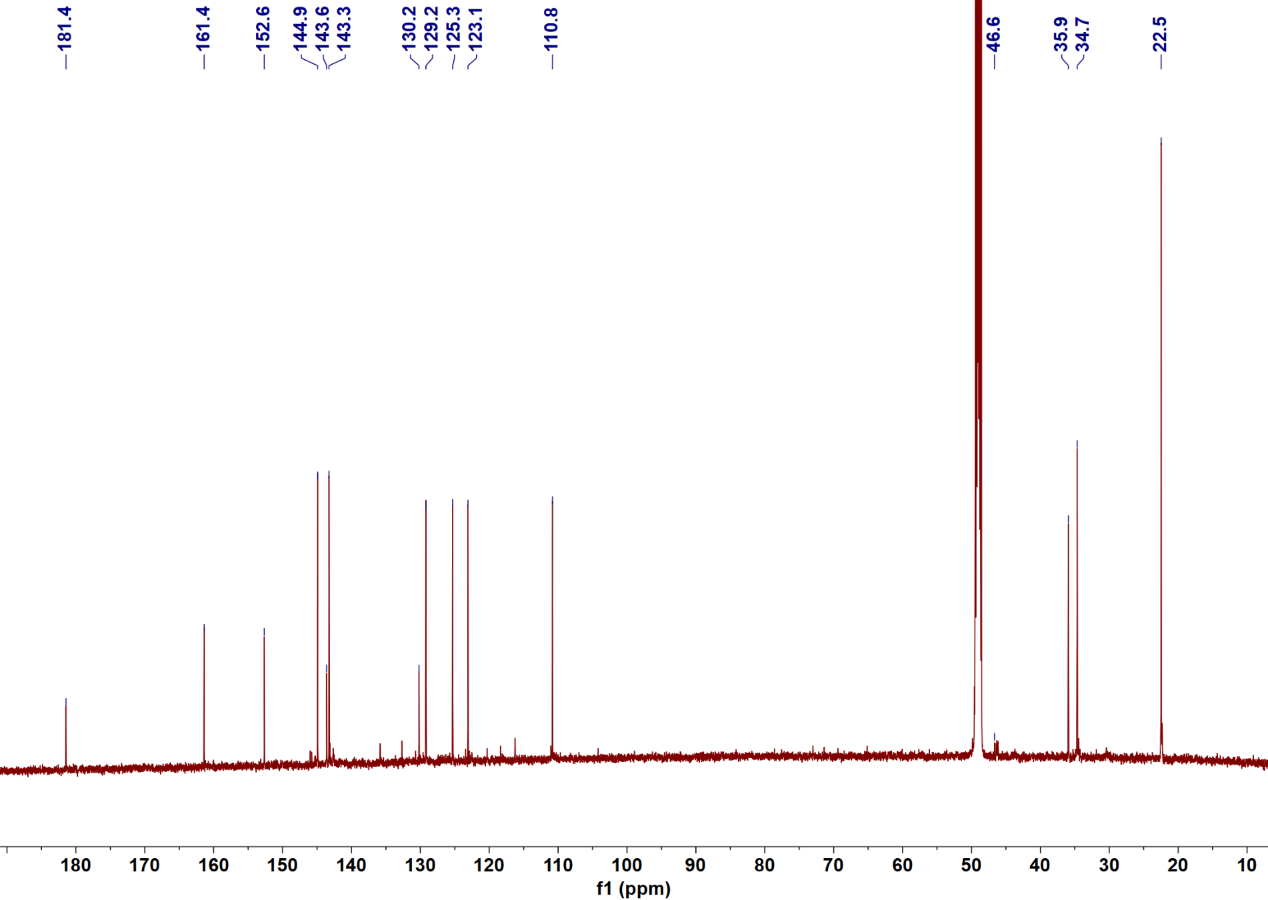


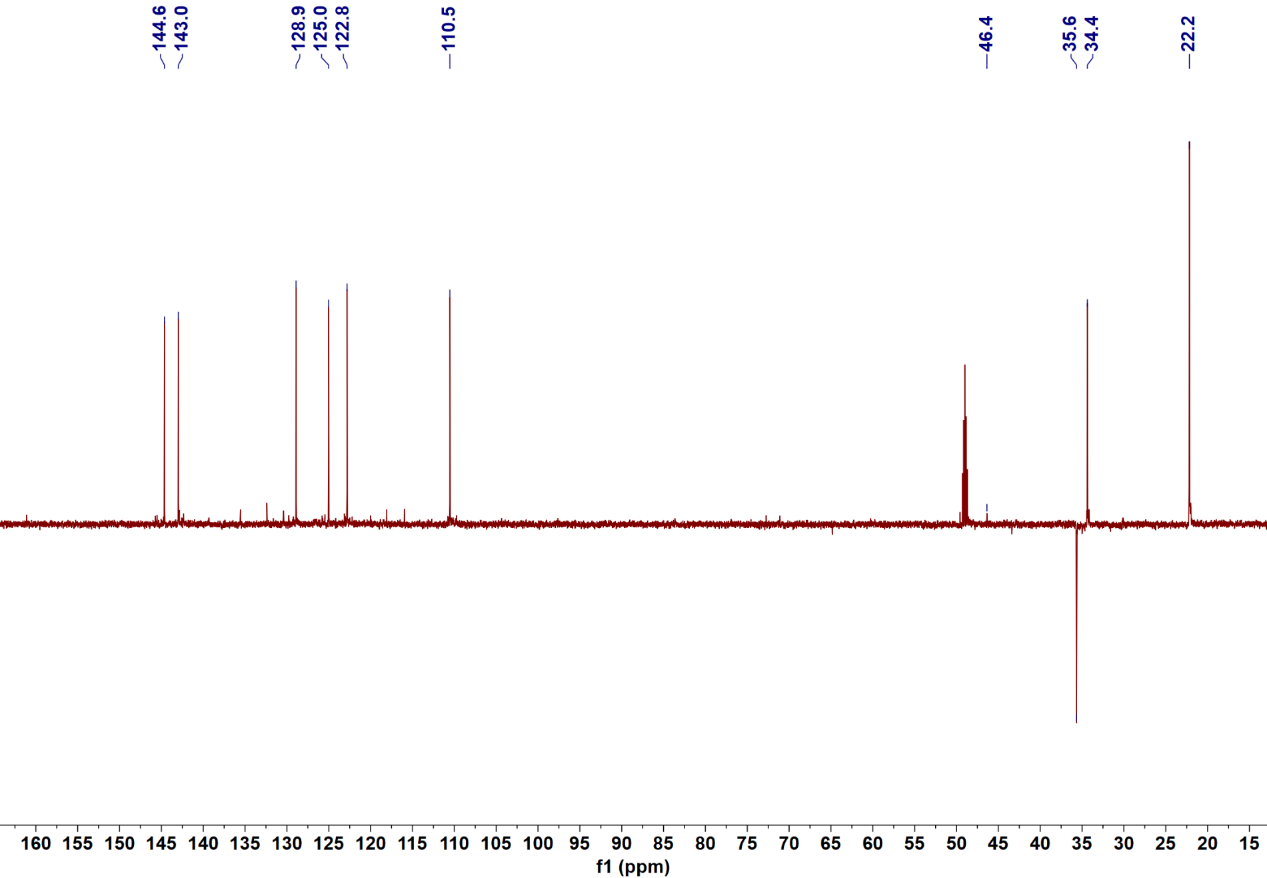


**Figure S23.** ^13^C NMR spectrum of compound **3** in DMSO-*d*_6_ (150 MHz)


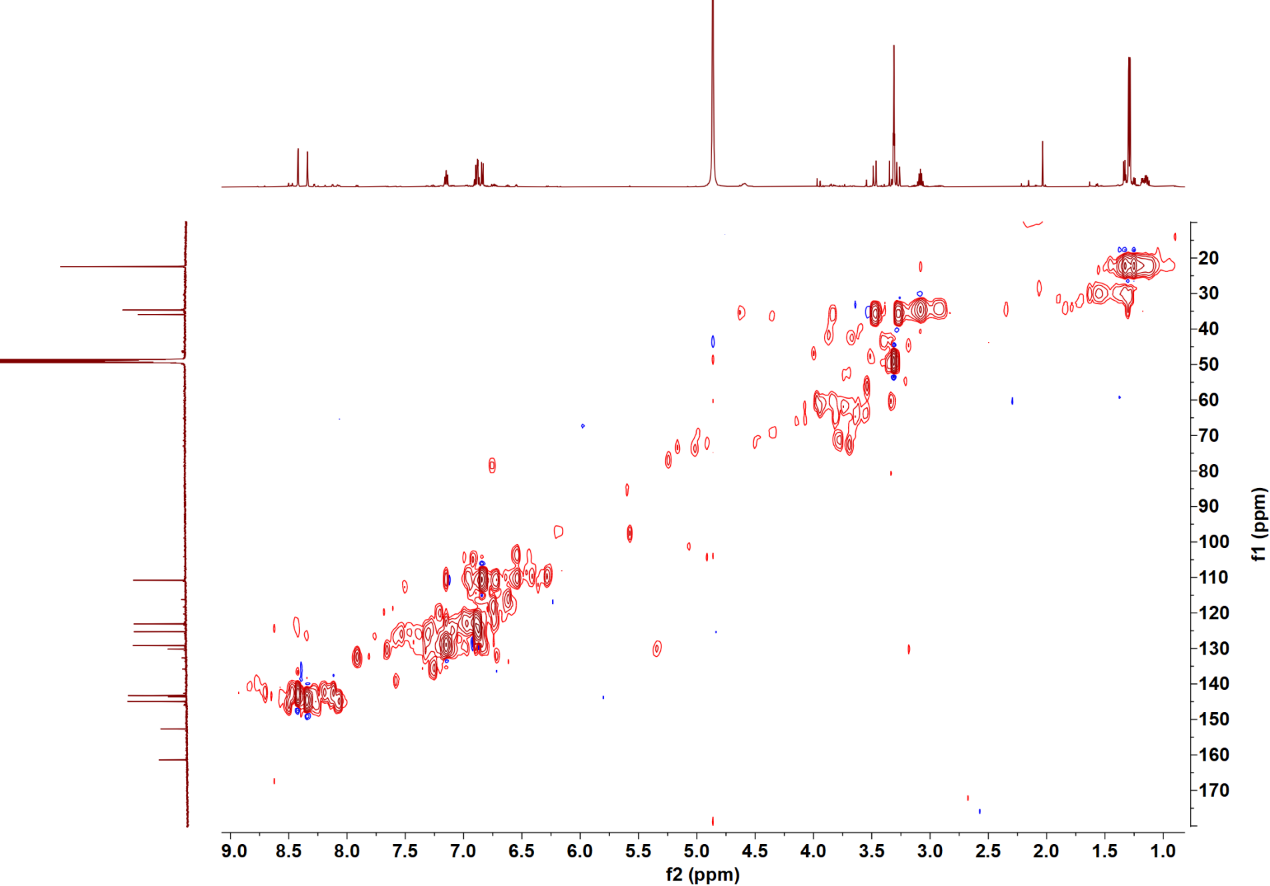


**Figure S24.** HSQC spectrum of compound **3** in DMSO-*d*_6_ (600 MHz)


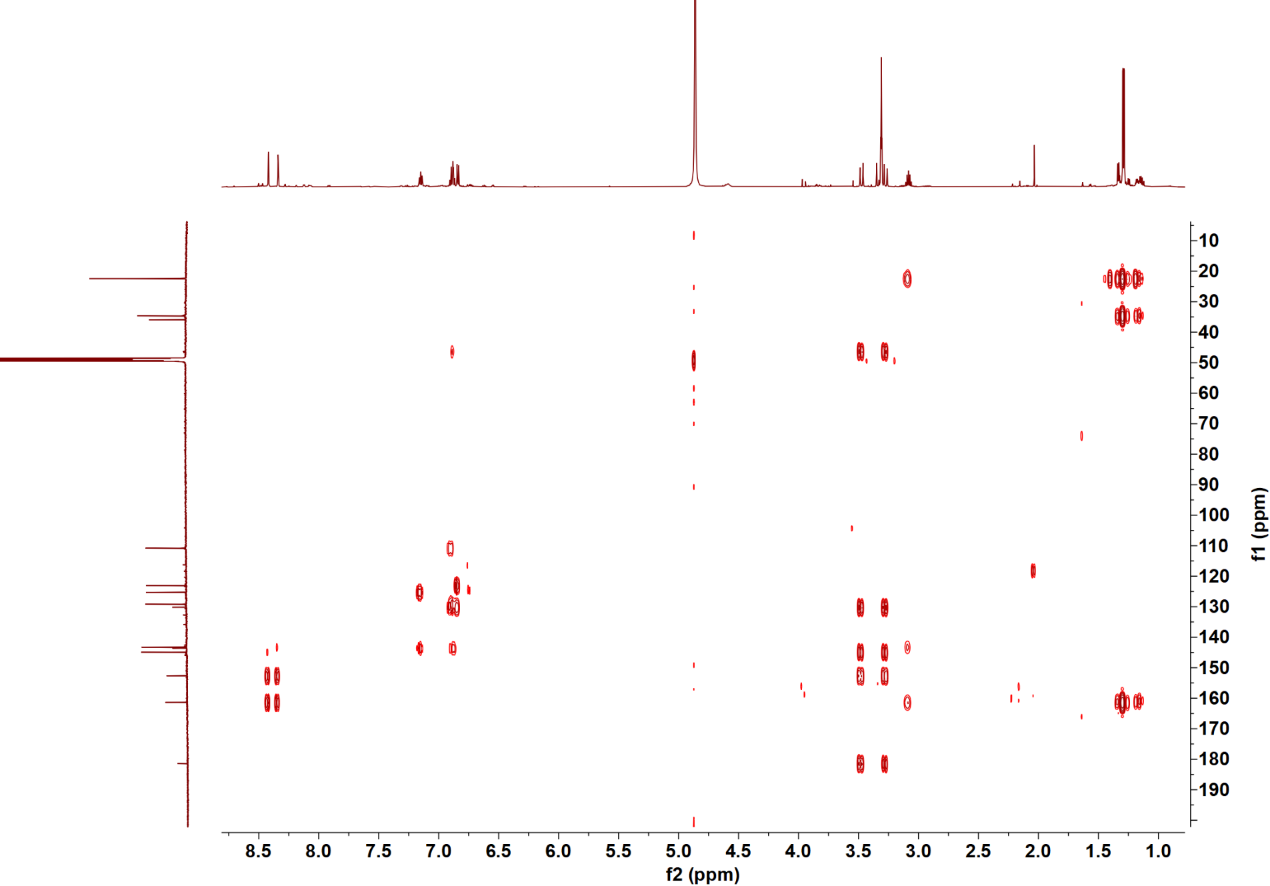


**Figure S25.** HMBC spectrum of compound **3** in DMSO-*d*_6_ (600 MHz)


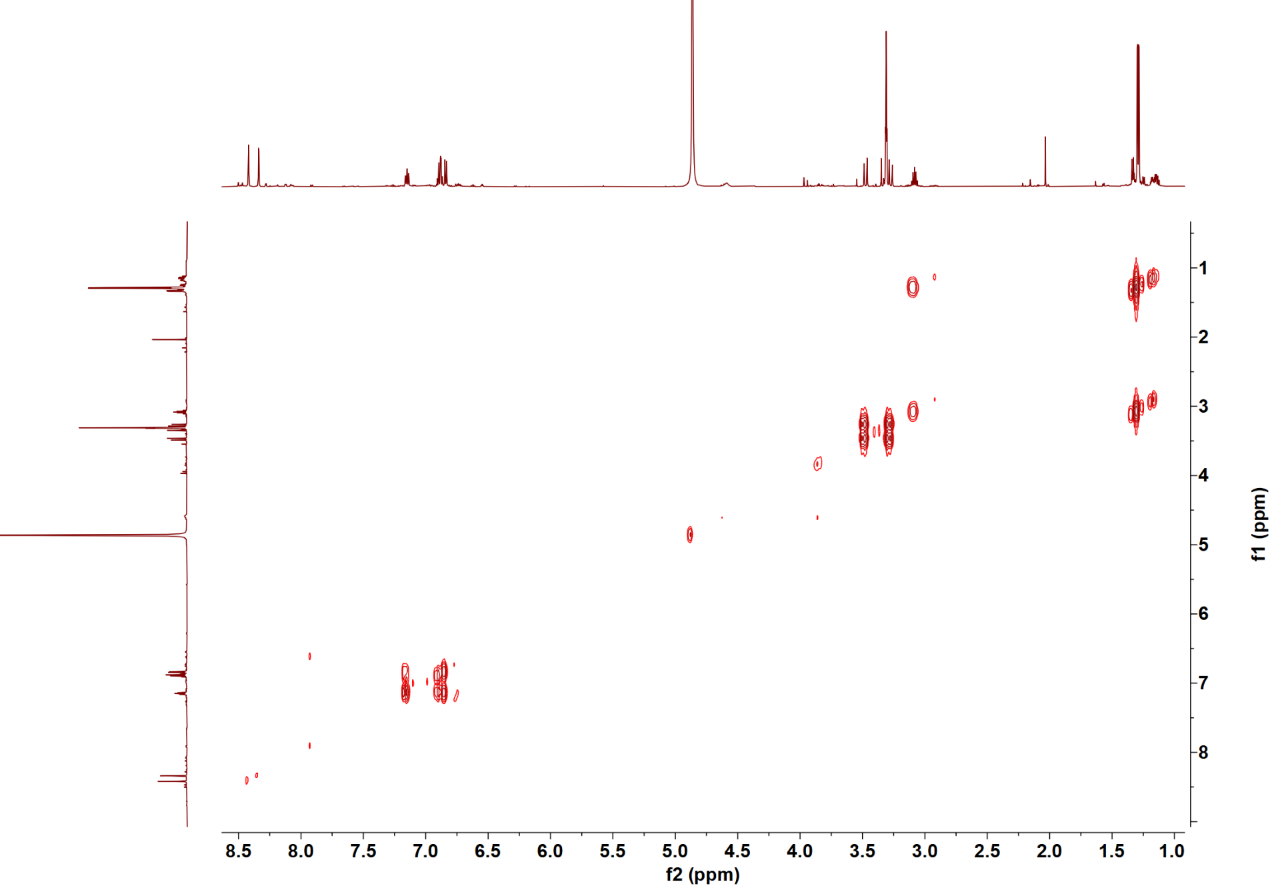


**Figure S26**. ^1^H-^1^H COSY spectrum of compound **3** in DMSO-*d*_6_ (600 MHz)


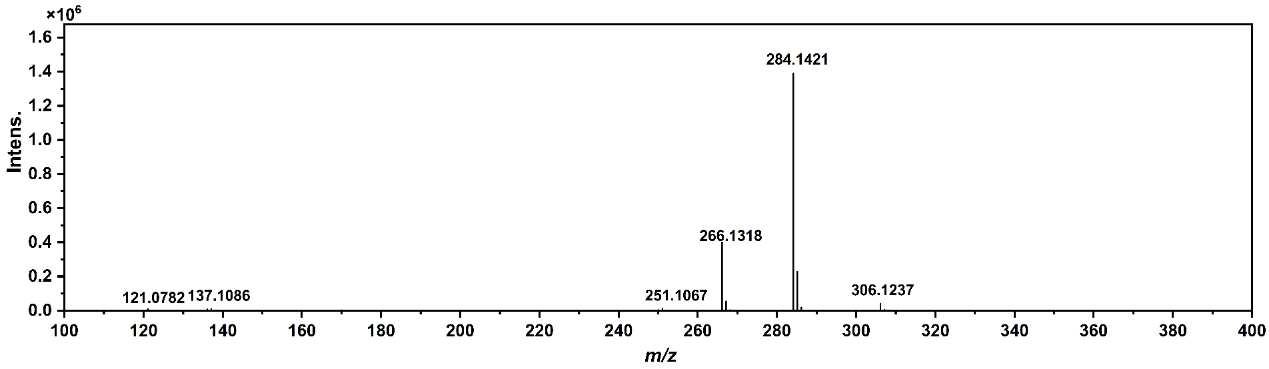


**Figure S27**. HRESIMS spectrum of compound **4**


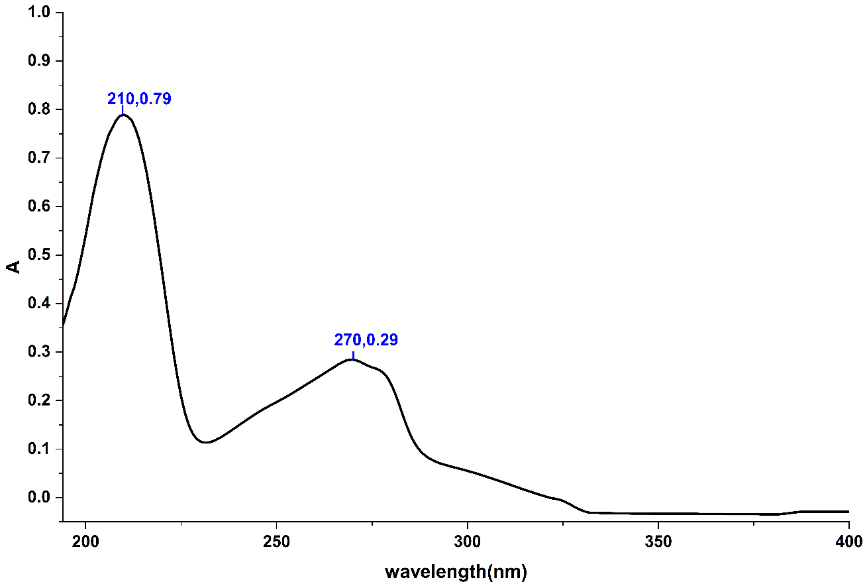


**Figure S28**. UV spectrum of compound **4**


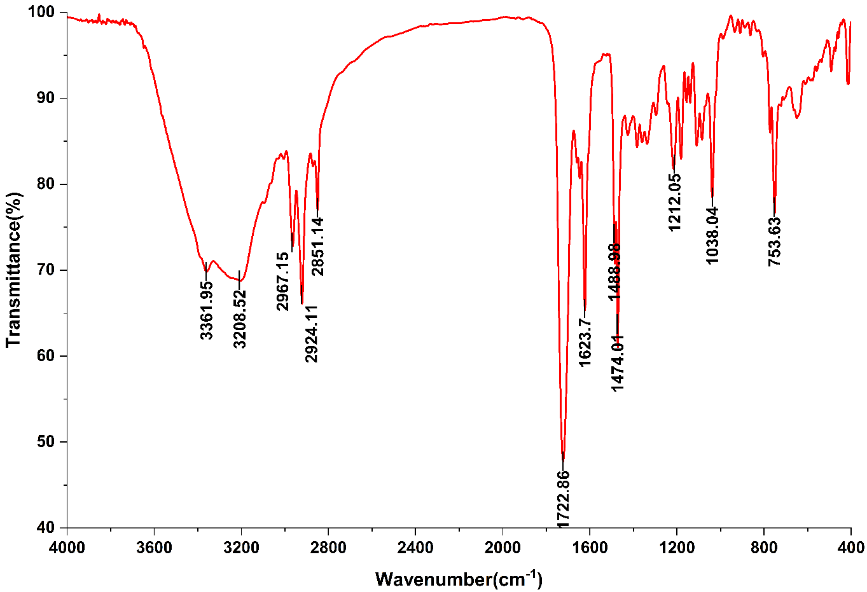


**Figure S29**. IR spectrum of compound **4**

**Figure S30.** ^1^H NMR spectrum of compound **4** in CD_3_OD (600 MHz)

**Figure S31.** ^13^C NMR spectrum of compound **4** in CD_3_OD (150 MHz)

**Figure S32.** HSQC spectrum of compound **4** in CD_3_OD (600 MHz)

**Figure S33.** HMBC spectrum of compound **4** in CD_3_OD (600 MHz)

**Figure S34**. ^1^H-^1^H COSY spectrum of compound **4** in CD_3_OD (600 MHz)


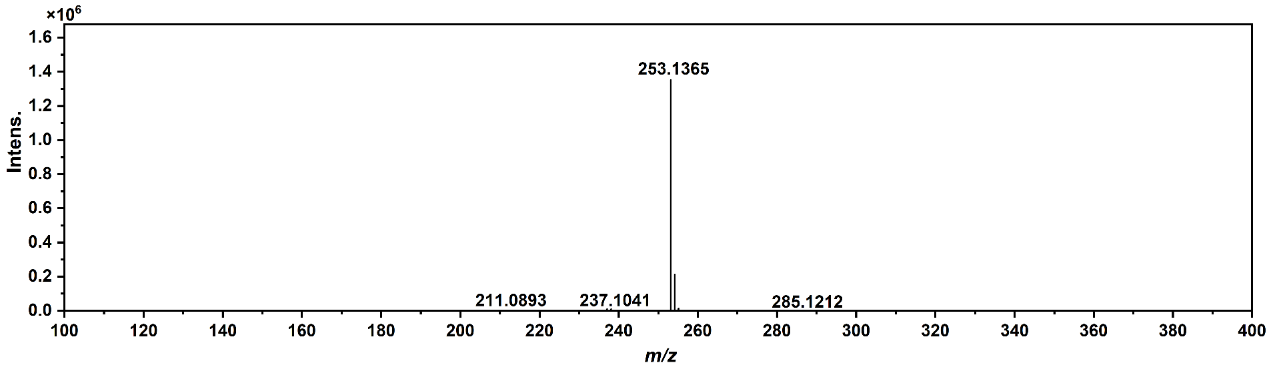


**Figure S35**. HRESIMS spectrum of compound **5**


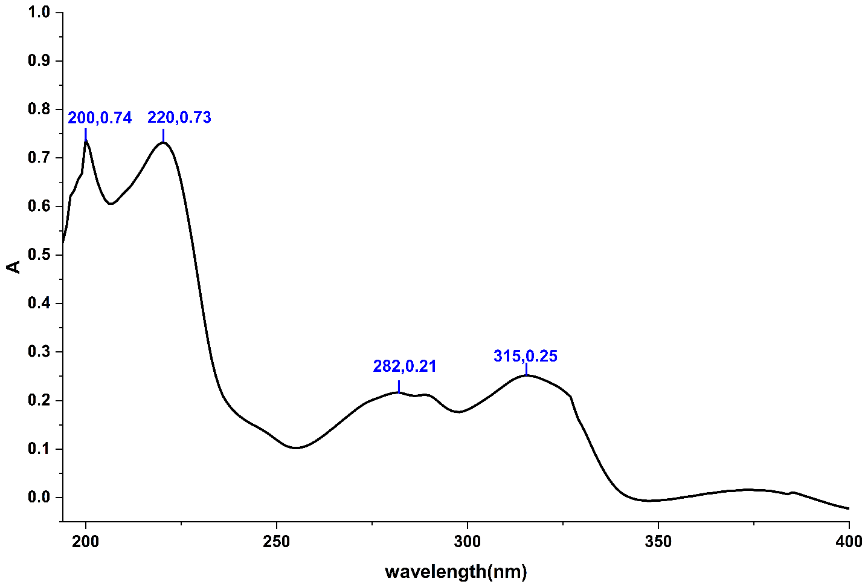


**Figure S36**. UV spectrum of compound **5**


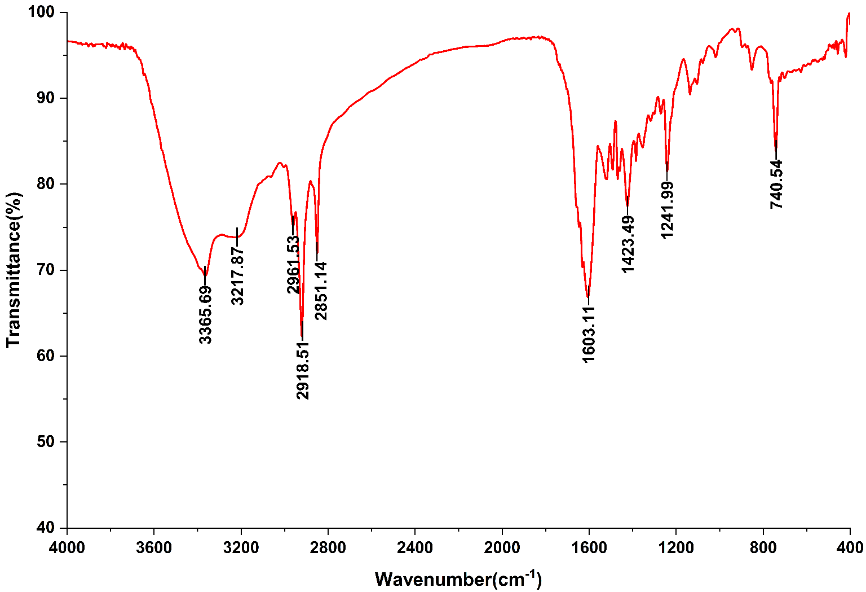


**Figure S37**. IR spectrum of compound **5**


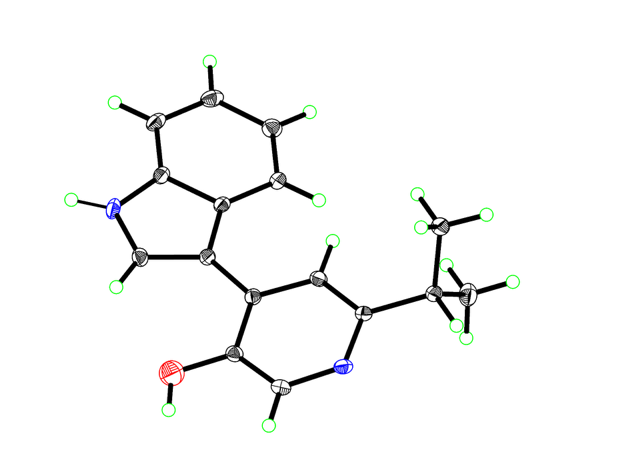


**Figure S38**. Crystal structure of compound **5**

**Figure S39.** ^1^H NMR spectrum of compound **5** in CD_3_OD (600 MHz)

**Figure S40.** ^13^C NMR spectrum of compound **5** in CD_3_OD (150 MHz)

**Figure S41.** HSQC spectrum of compound **5** in CD_3_OD (600 MHz)

**Figure S42.** HMBC spectrum of compound **5** in CD_3_OD (600 MHz)

**Figure S43**. ^1^H-^1^H COSY spectrum of compound **5** in CD_3_OD (600 MHz)


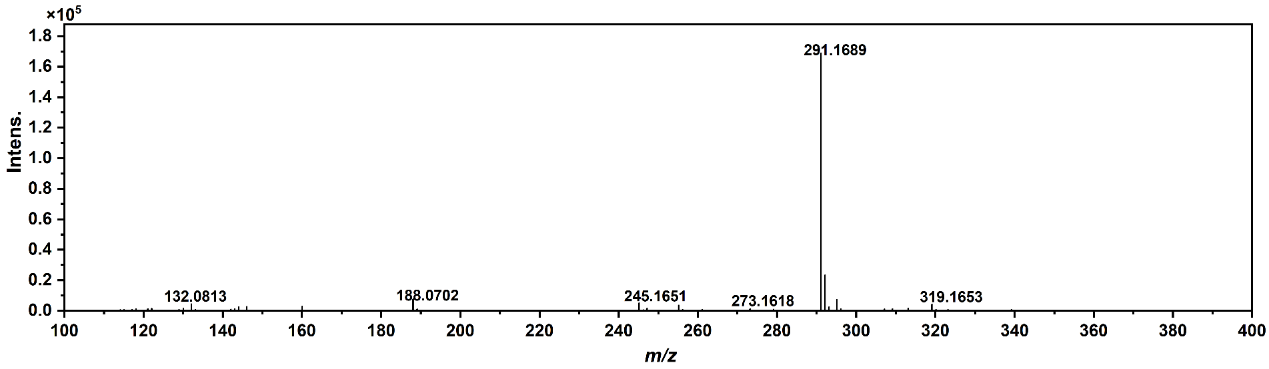


**Figure S44**. HRESIMS spectrum of compound **6**


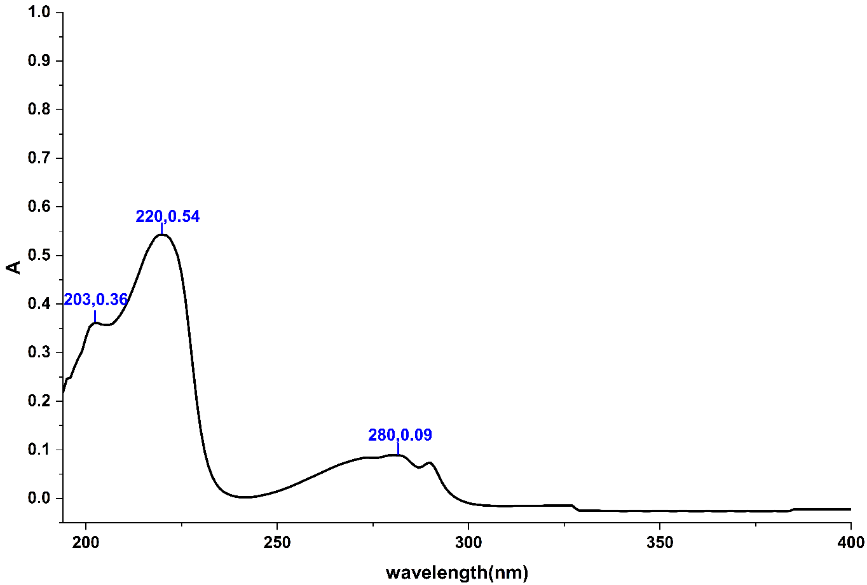


**Figure S45**. UV spectrum of compound **6**

**
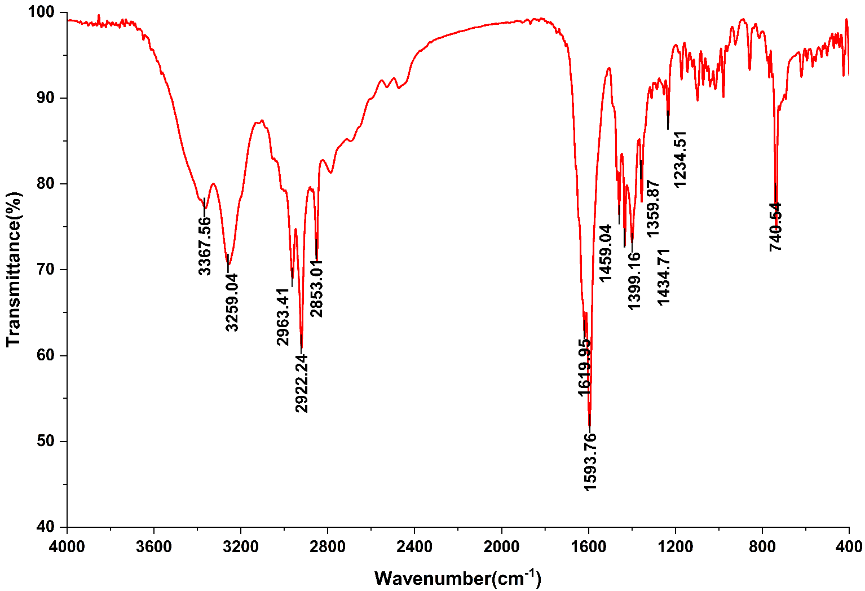
**

**Figure S46**. IR spectrum of compound **6**

**Figure S47.** ^1^H NMR spectrum of compound **6** in DMSO-*d*_6_(600 MHz)

**Figure S48.** ^13^C NMR spectrum of compound **6** in DMSO-*d*_6_ (150 MHz)

**Figure S49.** HSQC spectrum of compound **6** in DMSO-*d*_6_ (600 MHz)

**Figure S50.** HMBC spectrum of compound **6** in DMSO-*d*_6_ (600 MHz)

**Figure S51**. ^1^H-^1^H COSY spectrum of compound **6** in DMSO-*d*_6_ (600 MHz)


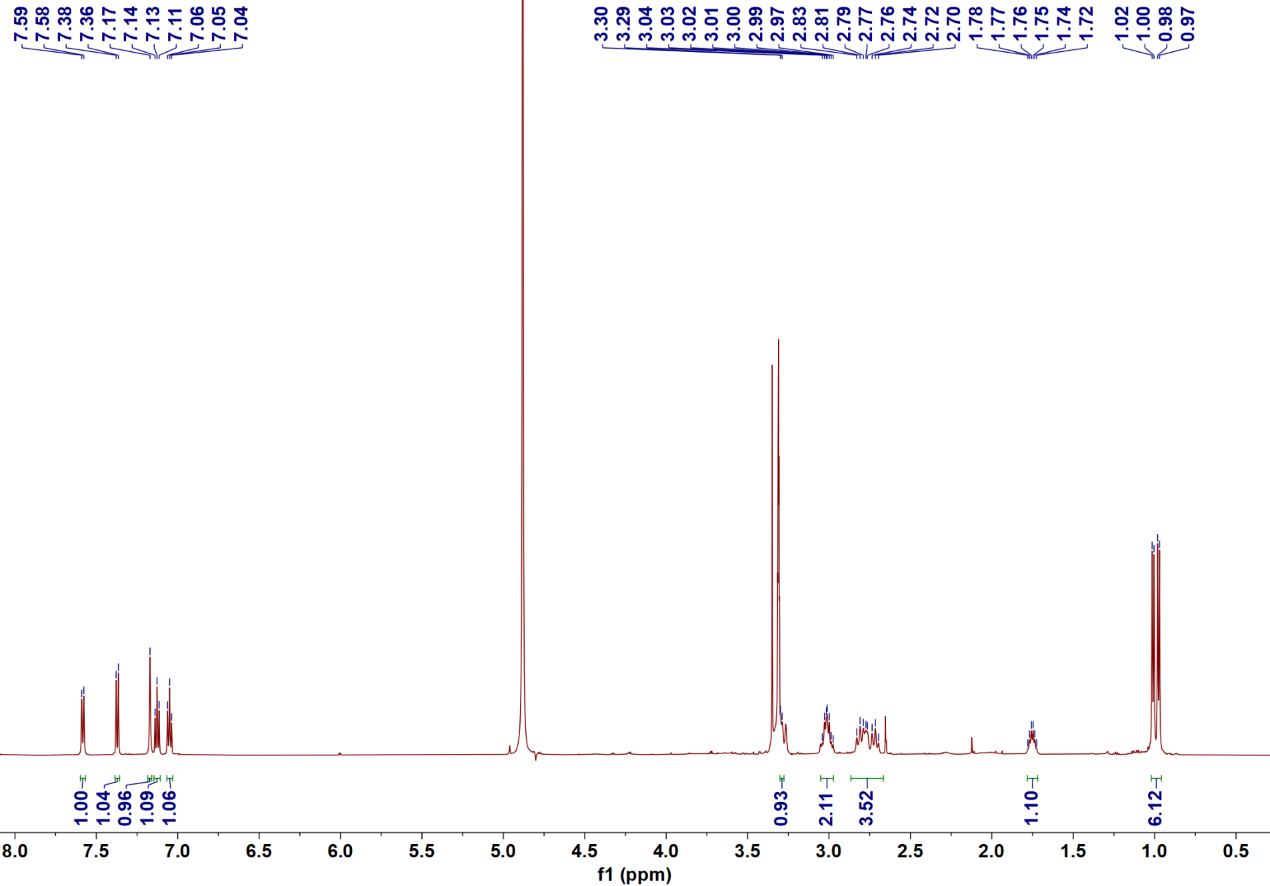


**Figure S52.** ^1^H NMR spectrum of compound **7** in CD_3_OD (600 MHz)


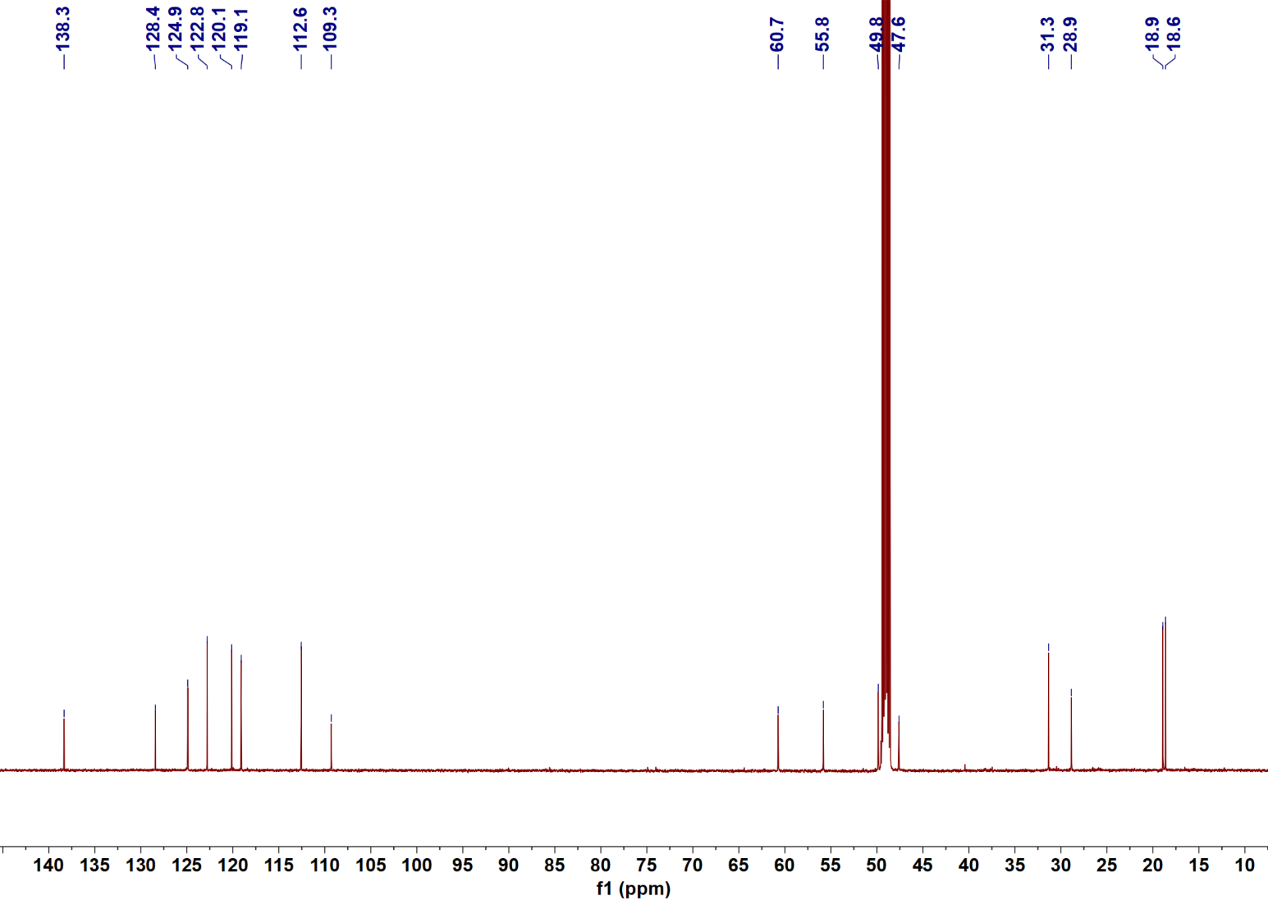


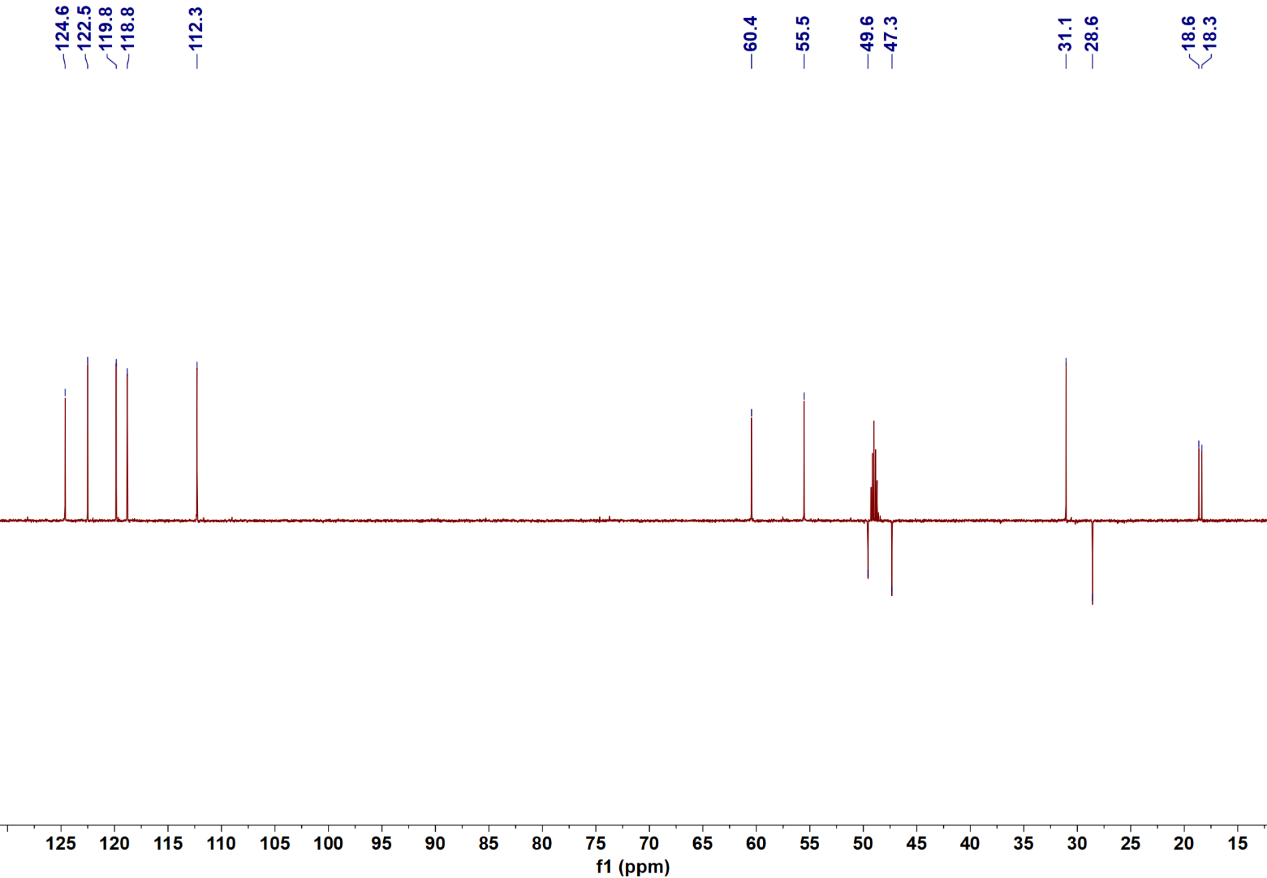


**Figure S53.** ^13^C NMR spectrum of compound **7** in CD_3_OD (150 MHz)


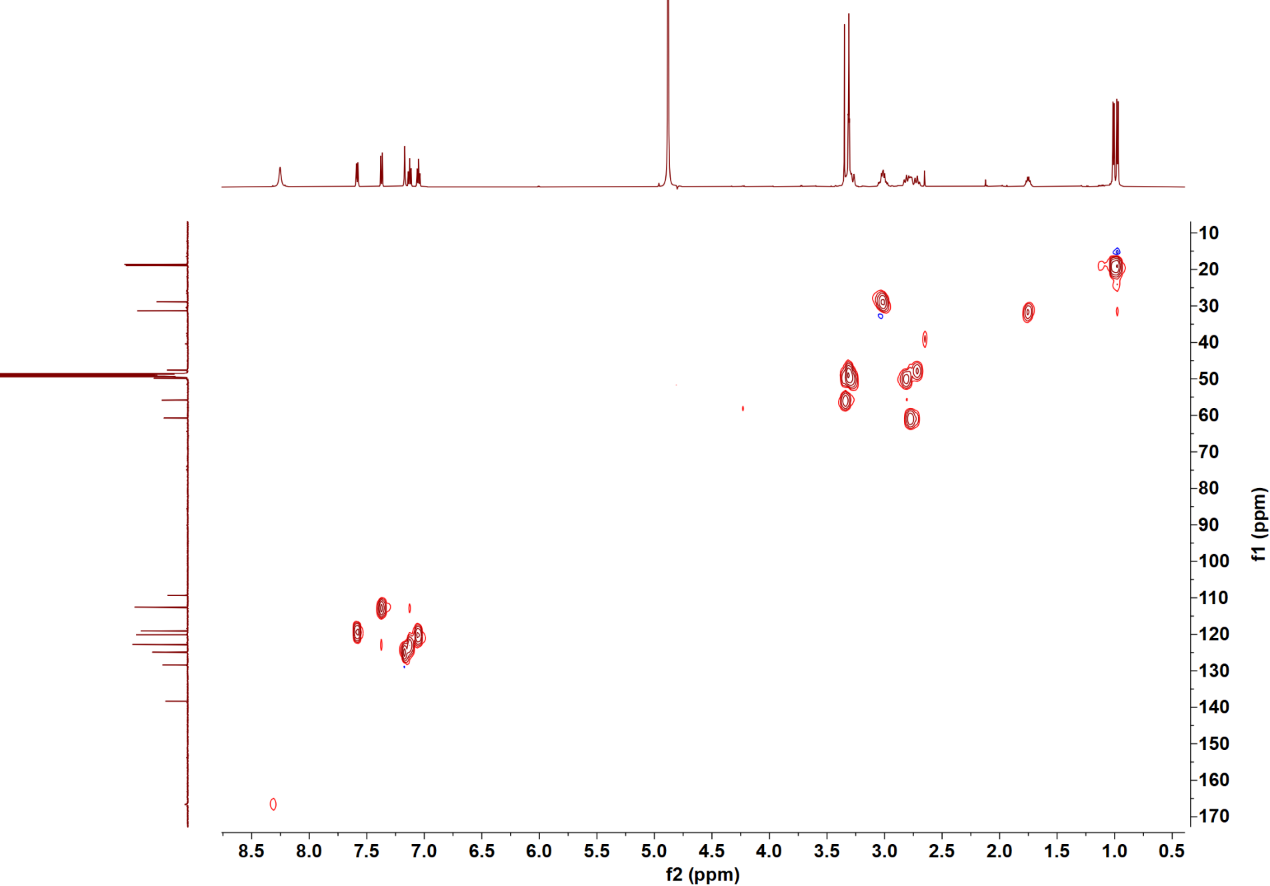


**Figure S54.** HSQC spectrum of compound **7** in CD_3_OD (600 MHz)


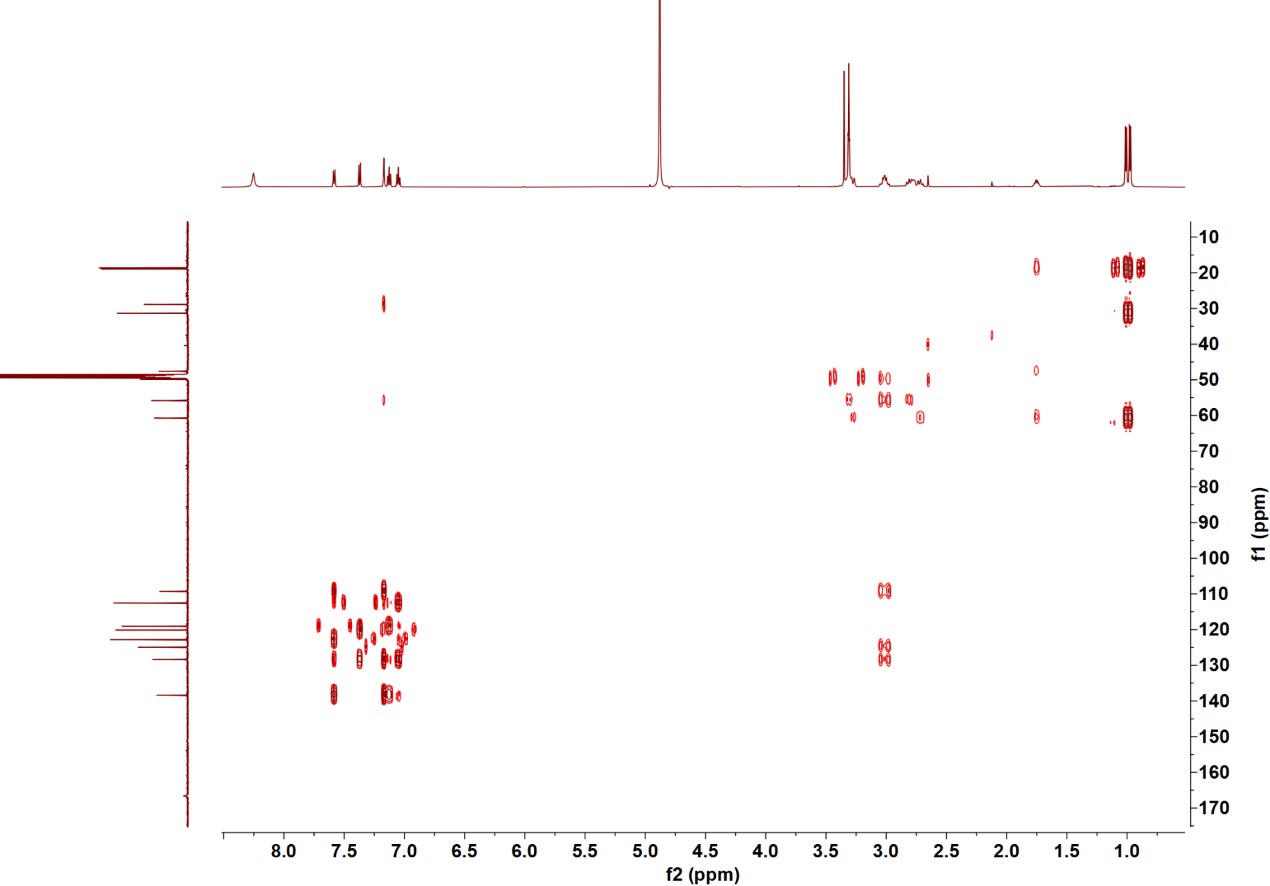


**Figure S55.** HMBC spectrum of compound **7** in CD_3_OD (600 MHz)


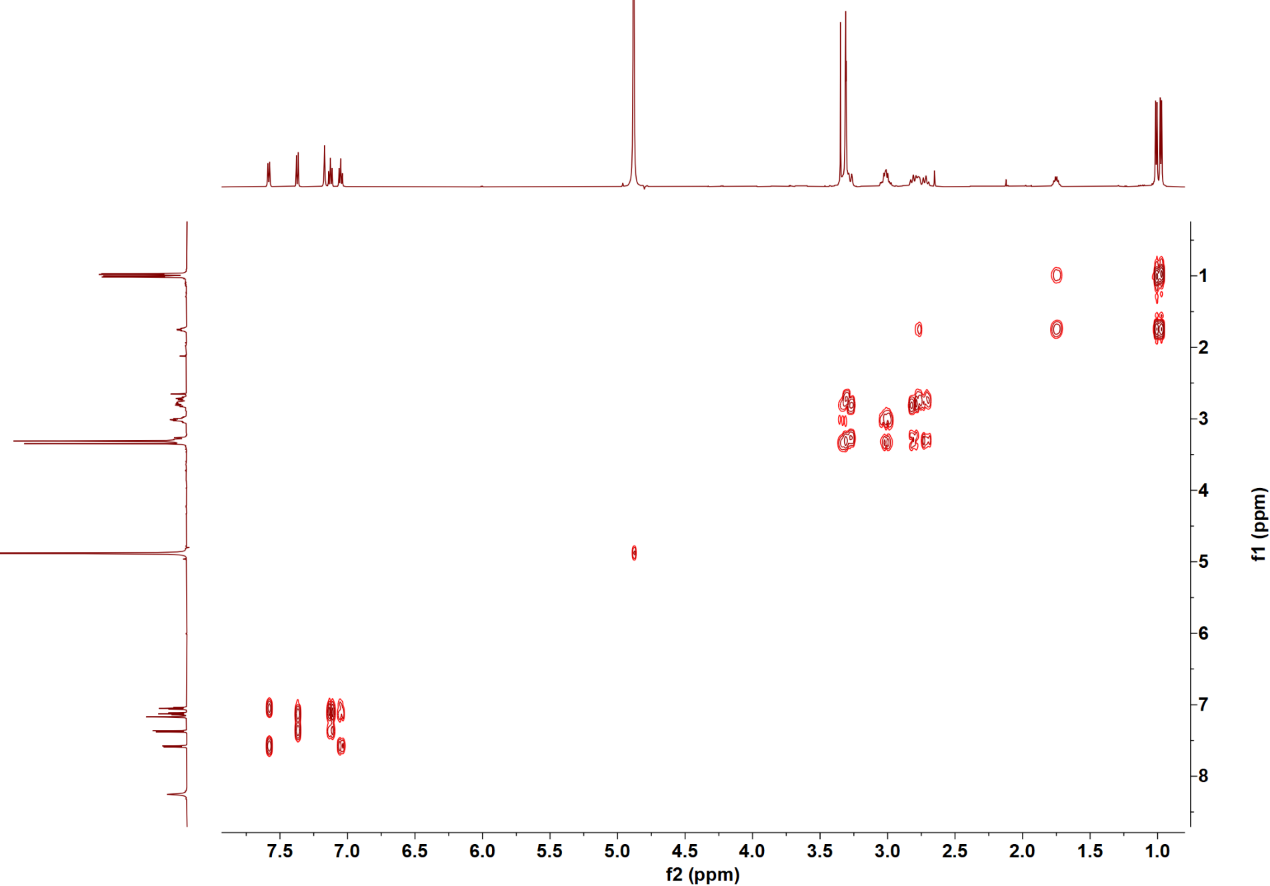


**Figure S56**. ^1^H-^1^H COSY spectrum of compound **7** in CD_3_OD (600 MHz)


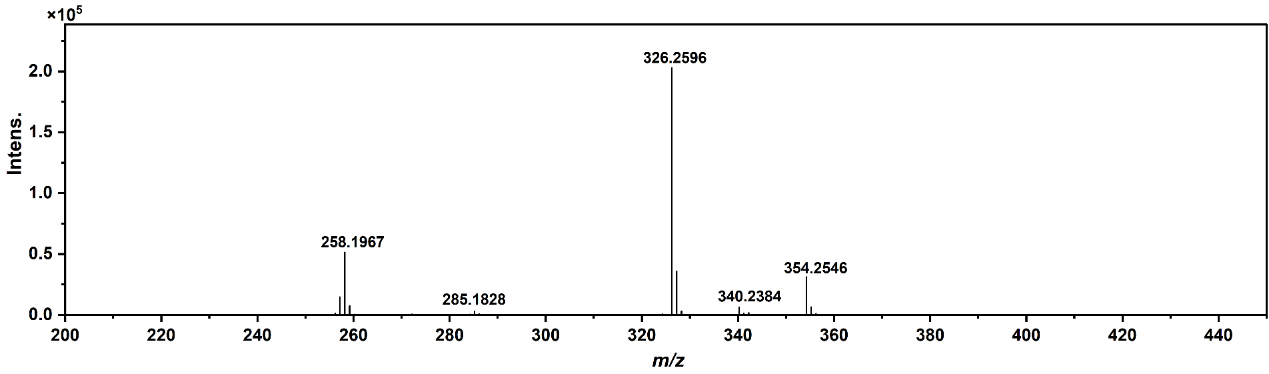


**Figure S57**. HRESIMS spectrum of compound **8**


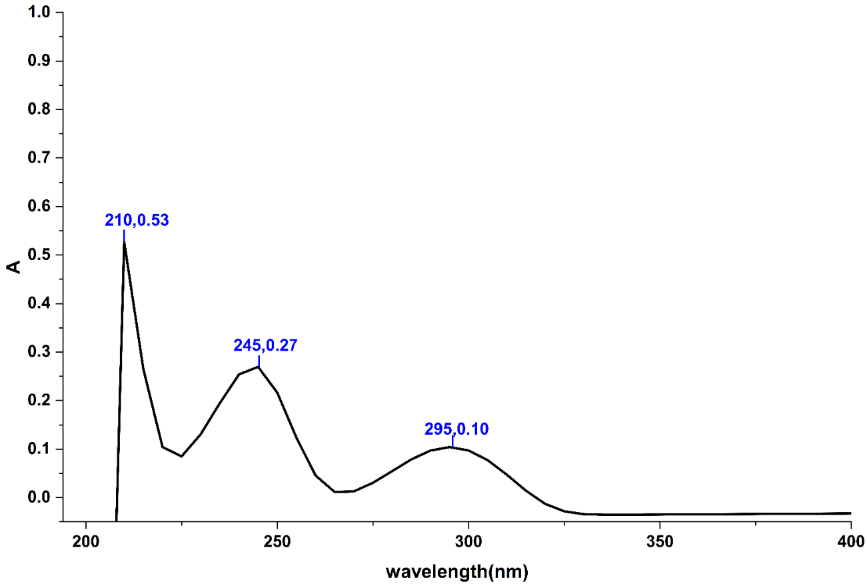


**Figure S58**. UV spectrum of compound **8**


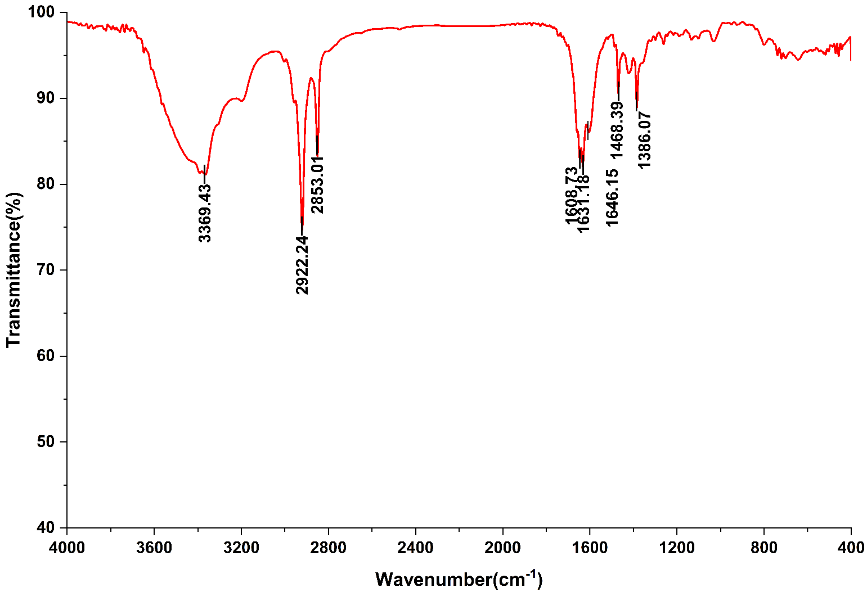


**Figure S59**. IR spectrum of compound **8**


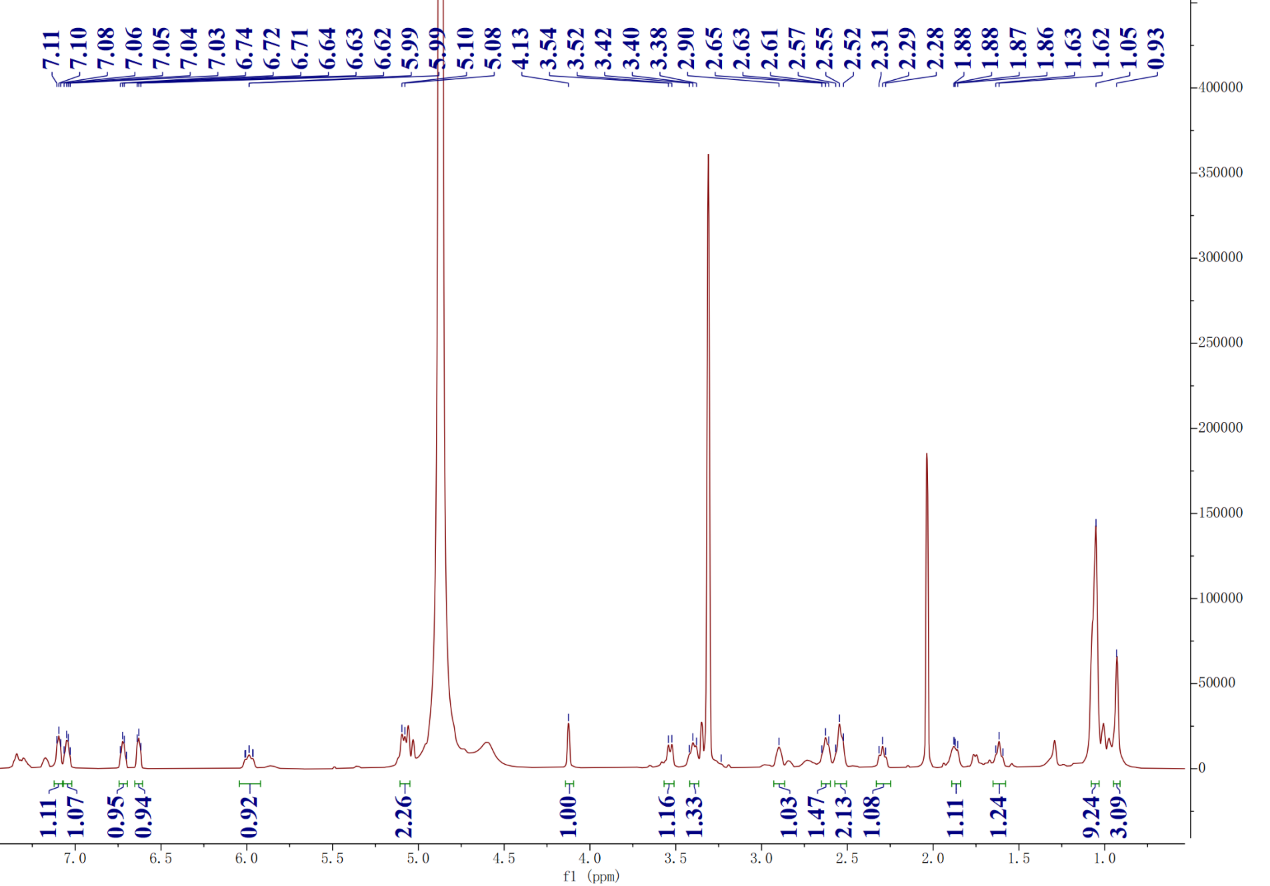


**Figure S60.** ^1^H NMR spectrum of compound **8** in CD_3_OD (600 MHz)


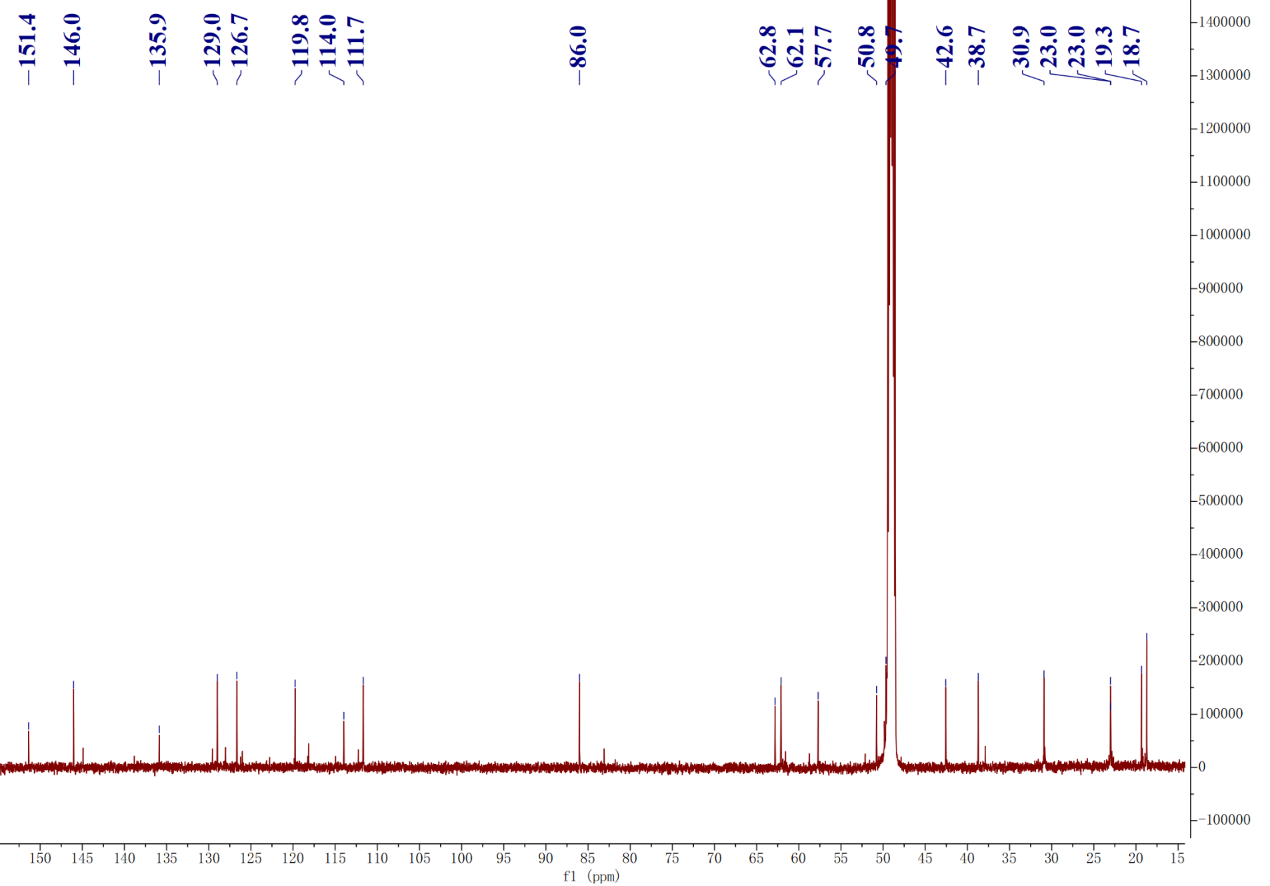


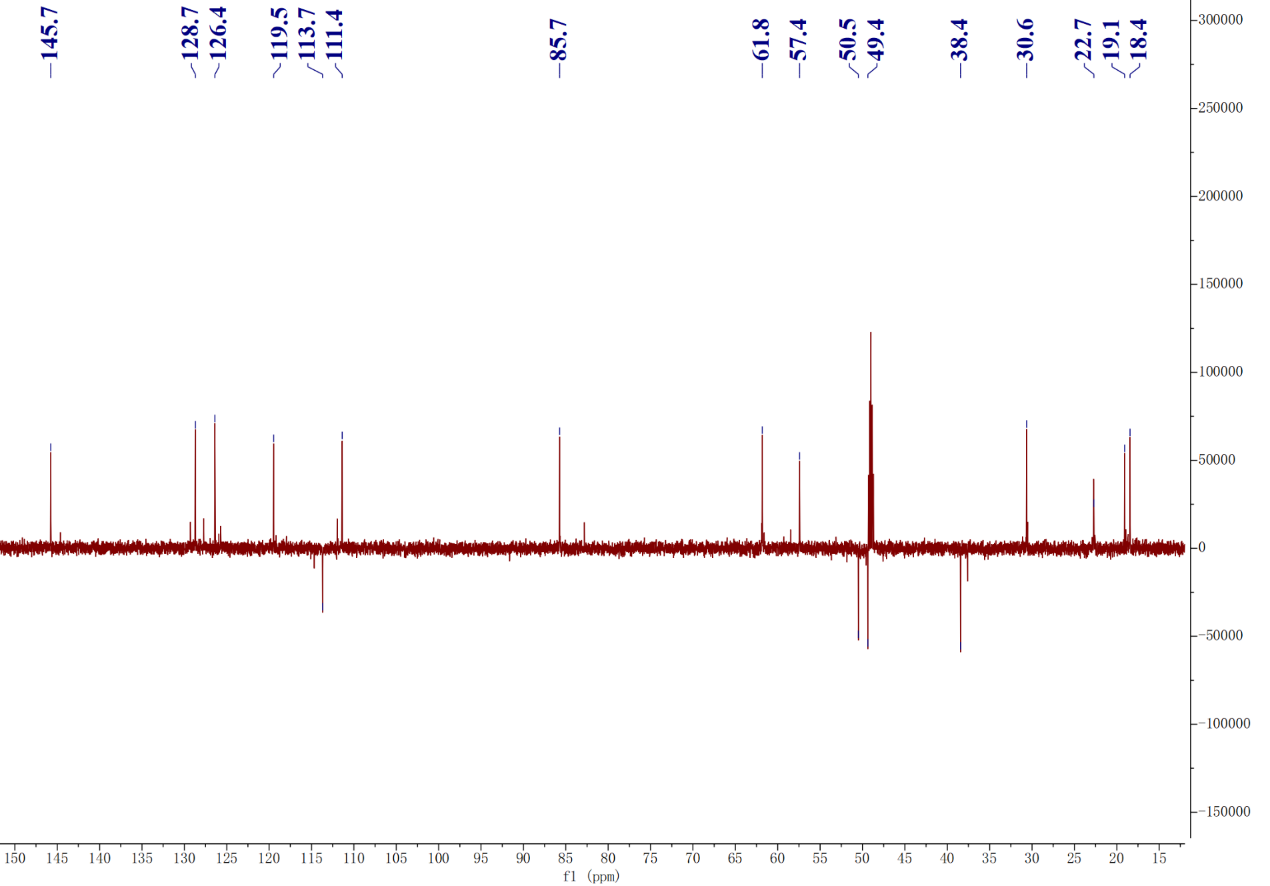


**Figure S61.** ^13^C NMR spectrum of compound **8** in CD_3_OD (150 MHz)


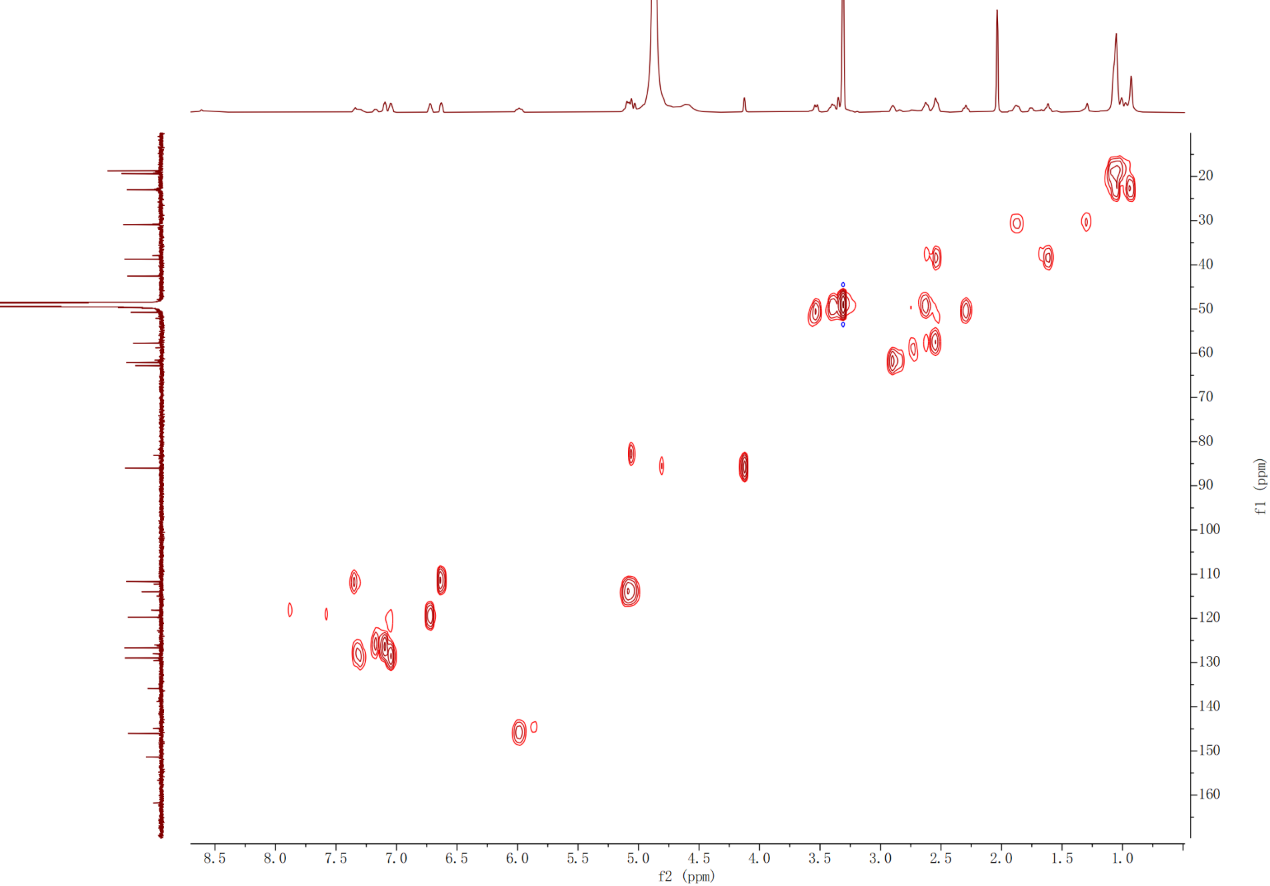


**Figure S62.** HSQC spectrum of compound **8** in CD_3_OD (600 MHz)


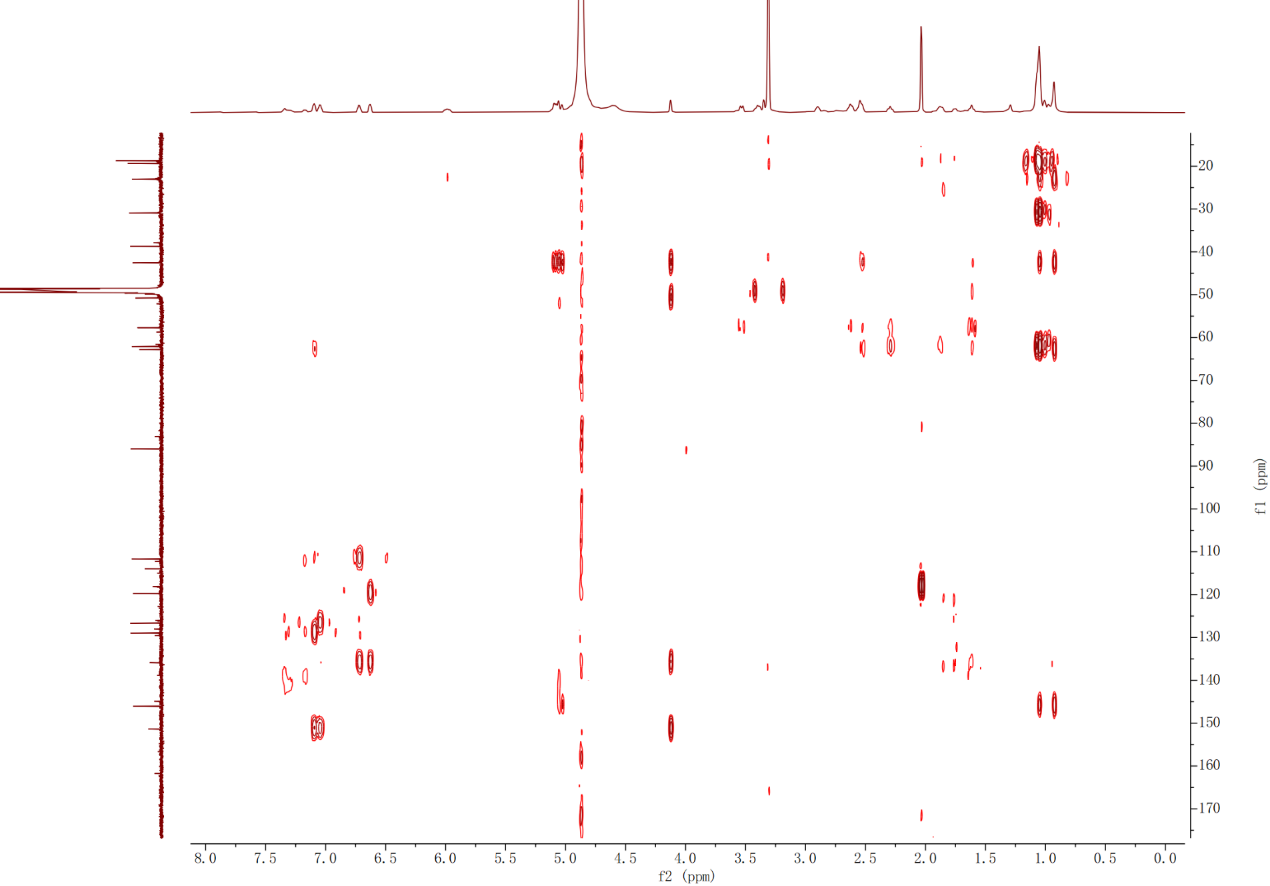


**Figure S63.** HMBC spectrum of compound **8** in CD_3_OD (600 MHz)


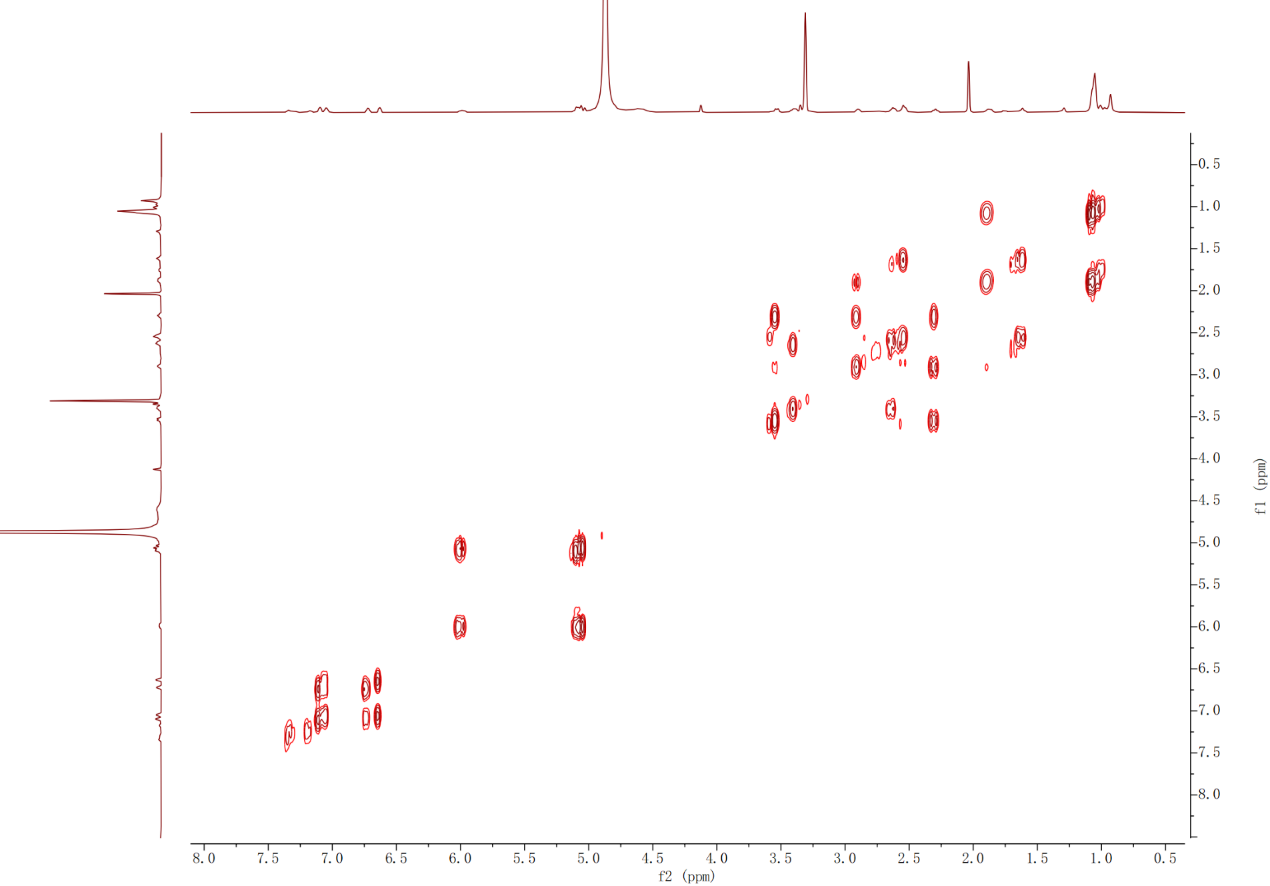


**Figure S64**. ^1^H-^1^H COSY spectrum of compound **8** in CD_3_OD (600 MHz)


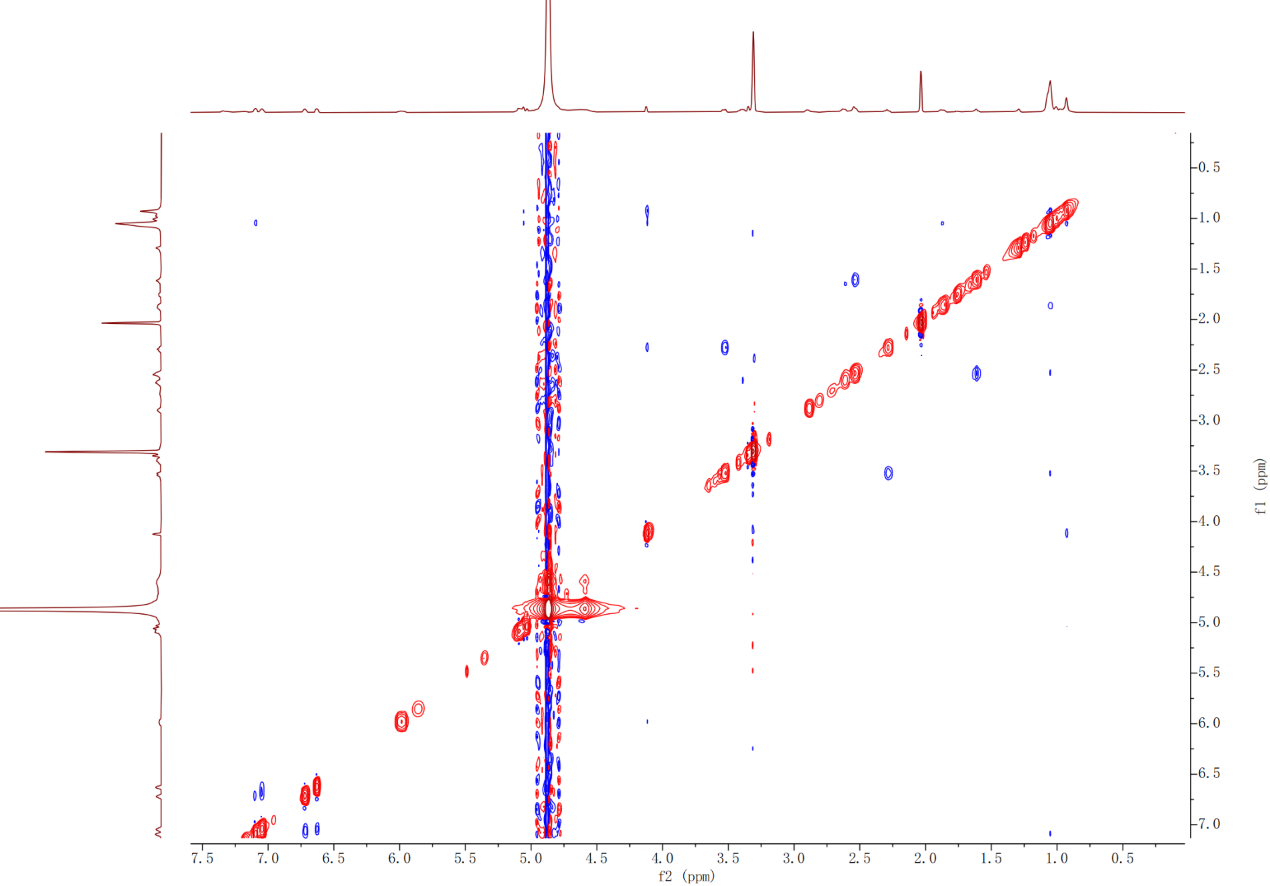


**Figure S65**. NOESY spectrum of compound **8** in CD_3_OD (600 MHz)


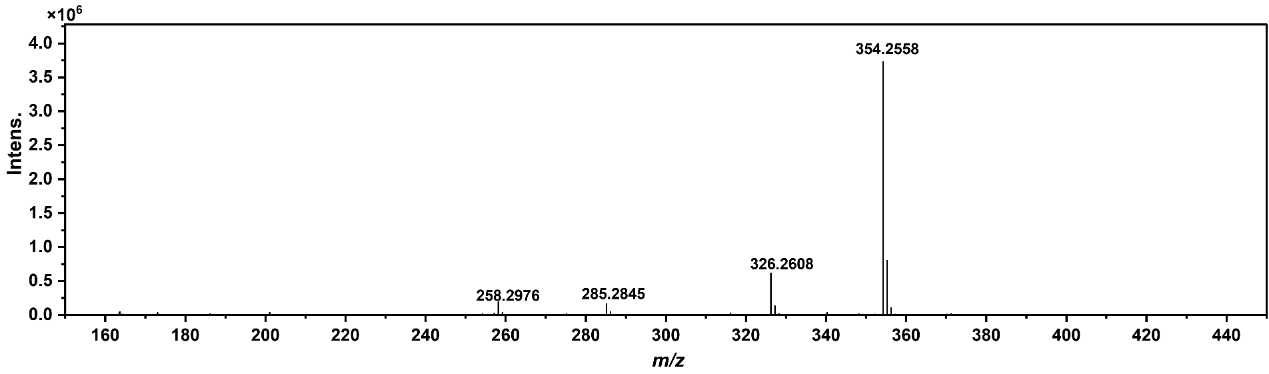


**Figure S66**. HRESIMS spectrum of compound **9**


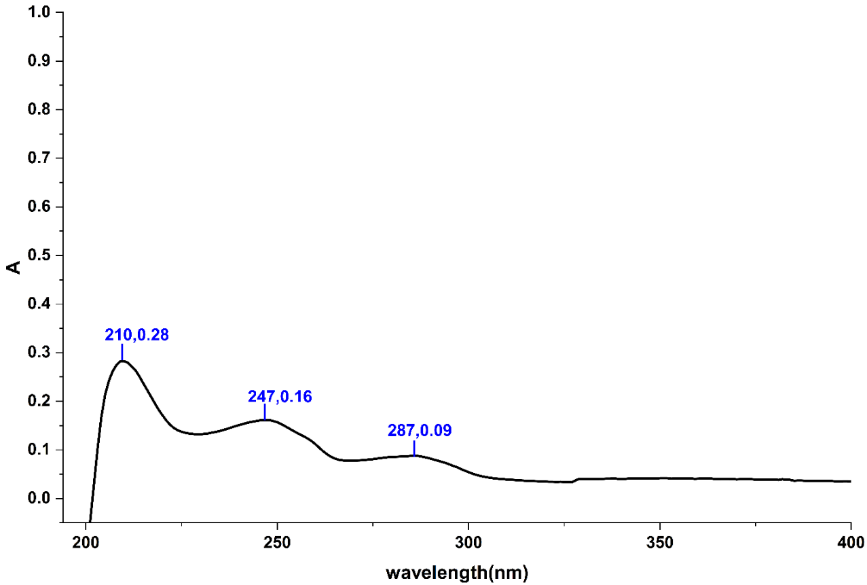


**Figure S67**. UV spectrum of compound **9**


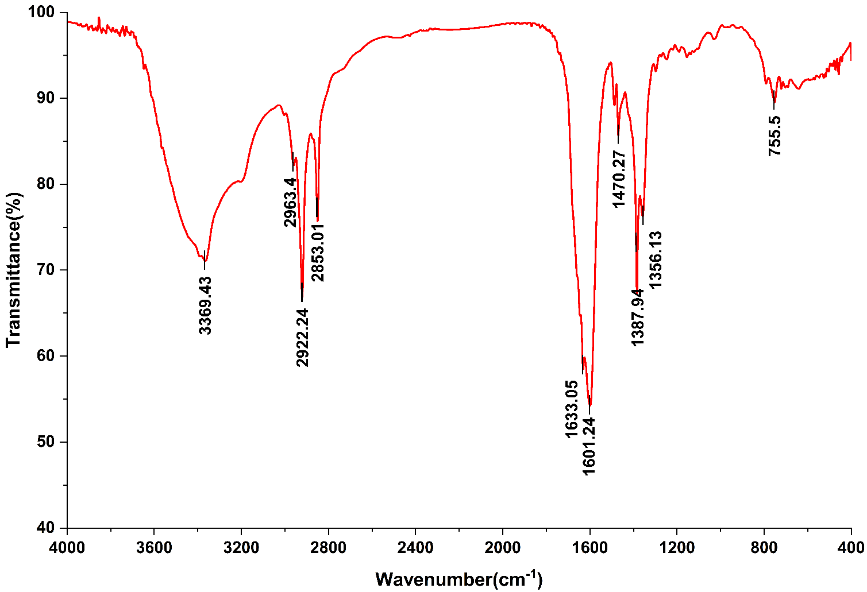


**Figure S68**. IR spectrum of compound **9**


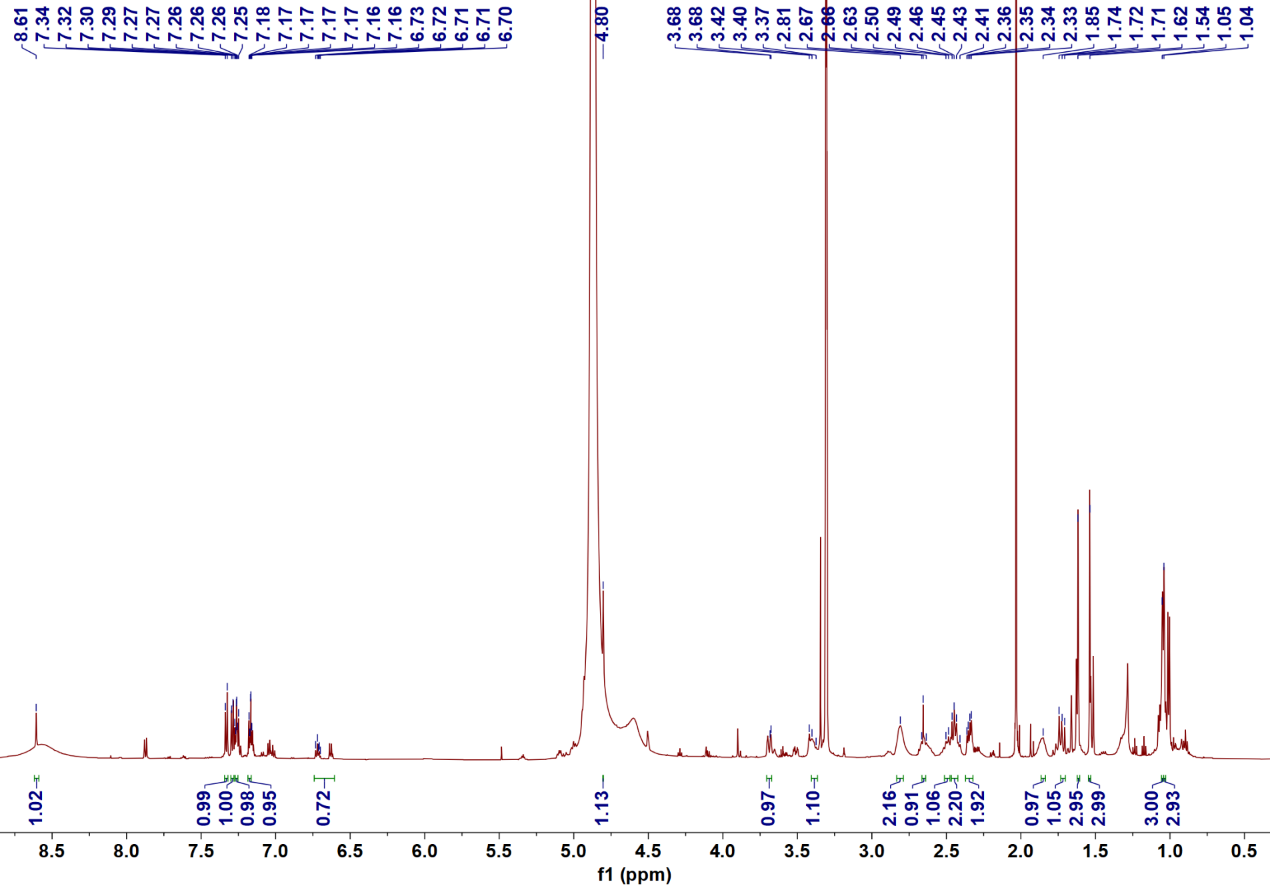


**Figure S69.** ^1^H NMR spectrum of compound **9** in CD_3_OD (600 MHz)


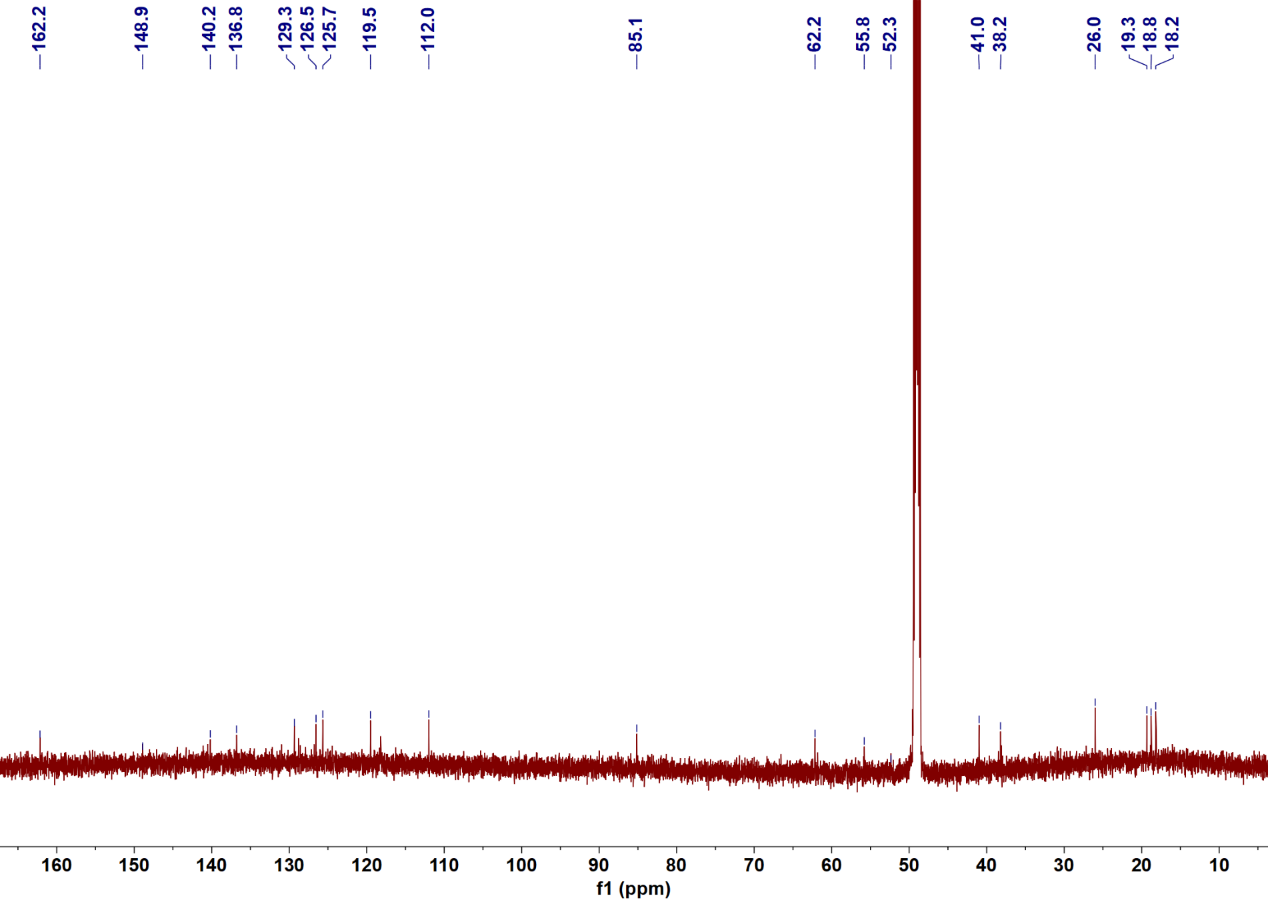


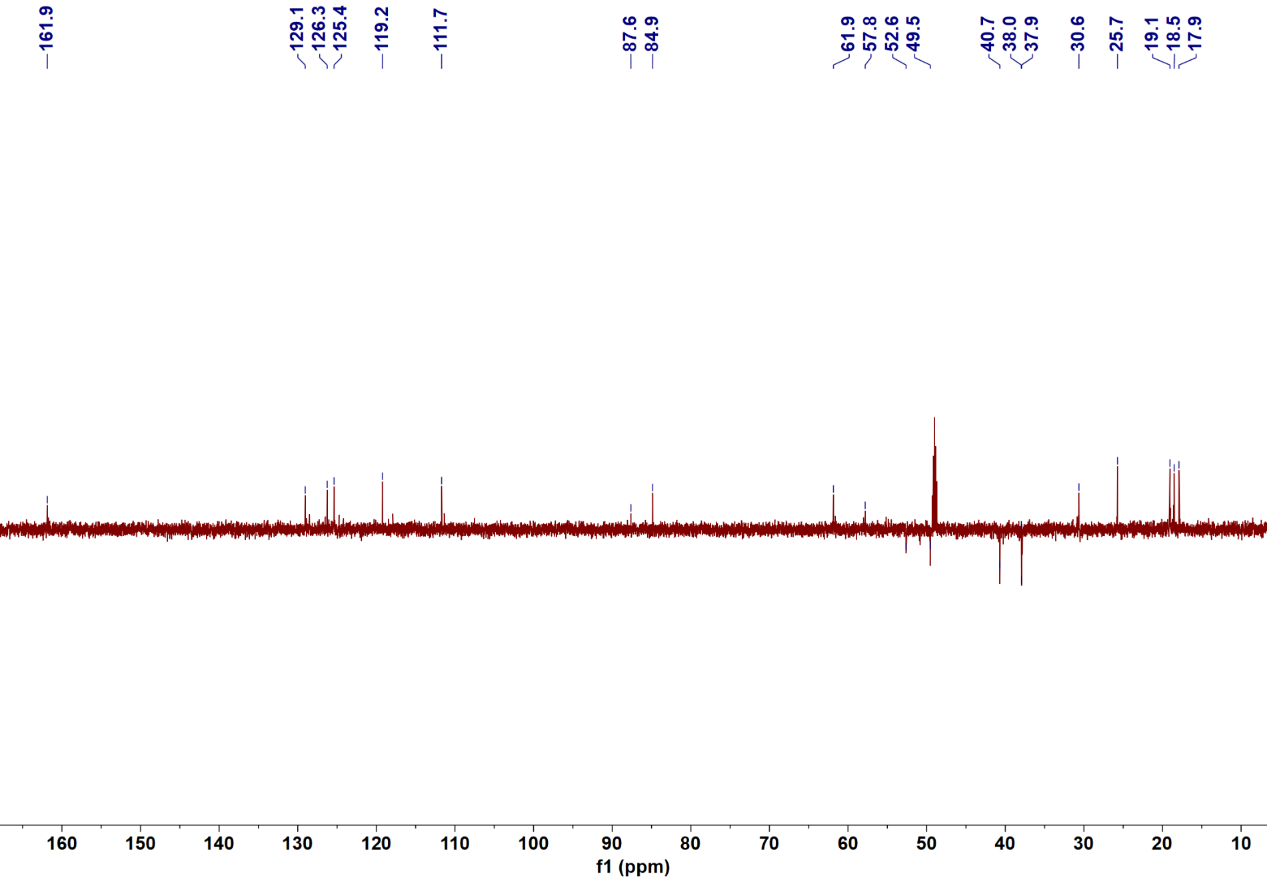


**Figure S70.** ^13^C NMR spectrum of compound **9** in CD_3_OD (150 MHz)


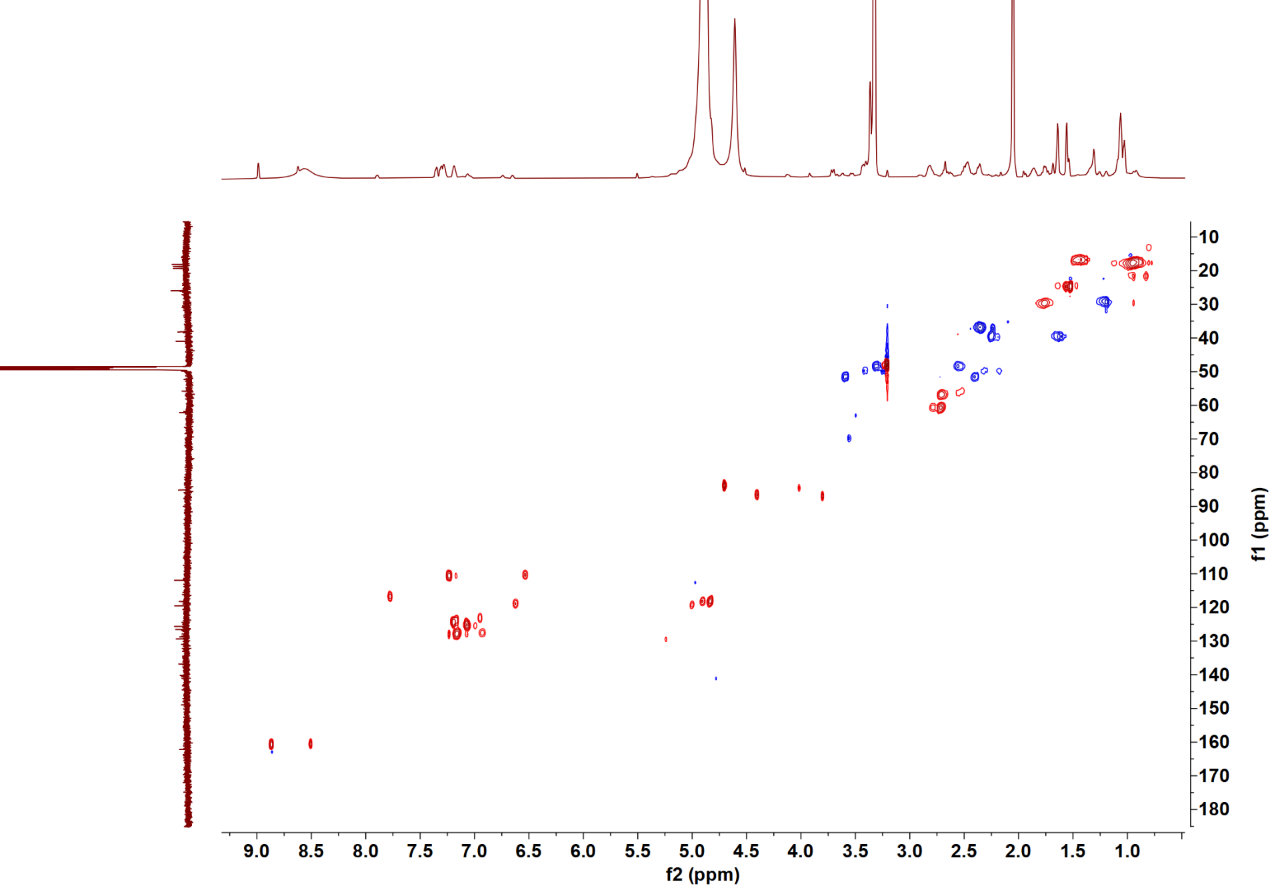


**Figure S71.** HSQC spectrum of compound **9** in CD_3_OD (600 MHz)


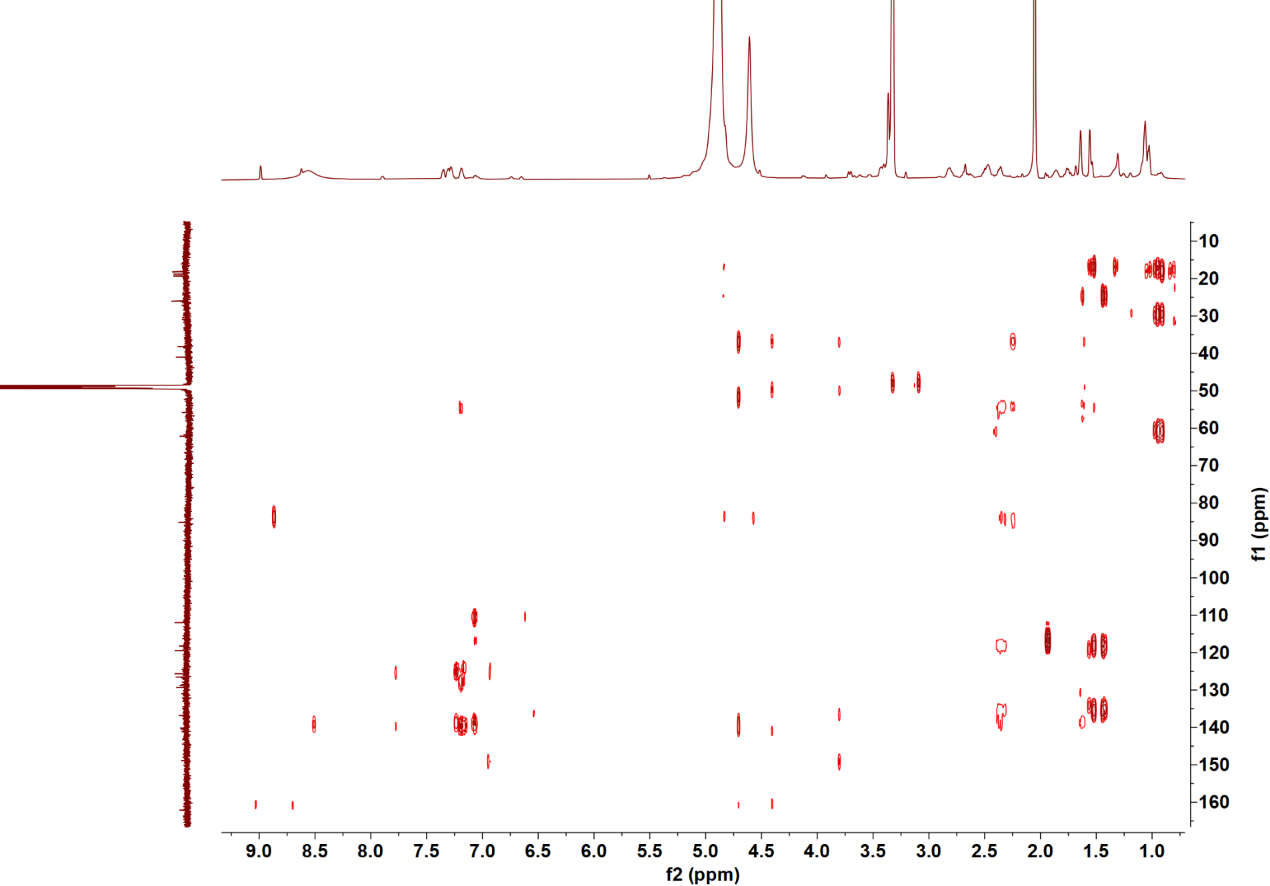


**Figure S72.** HMBC spectrum of compound **9** in CD_3_OD (600 MHz)


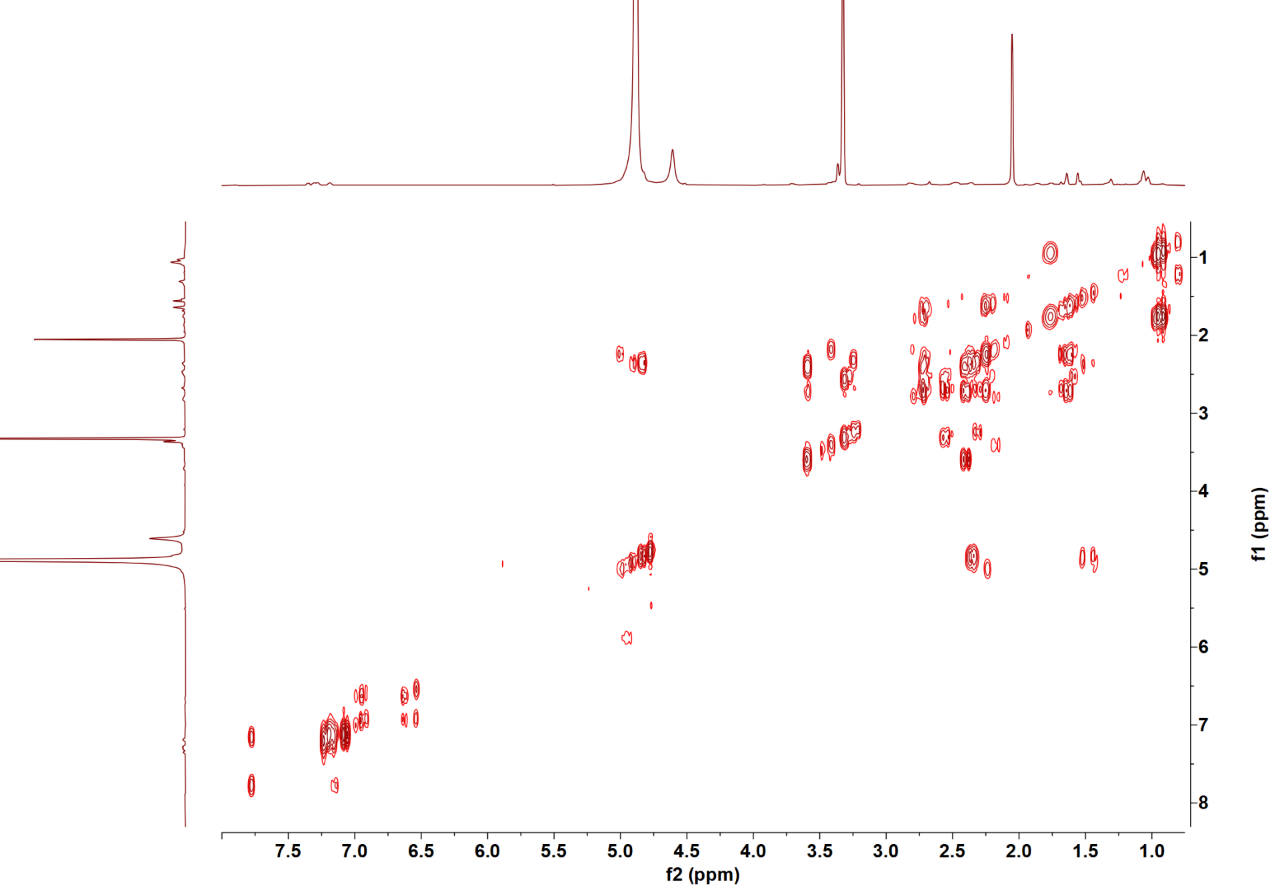


**Figure S73**. ^1^H-^1^H COSY spectrum of compound **9** in CD_3_OD (600 MHz)


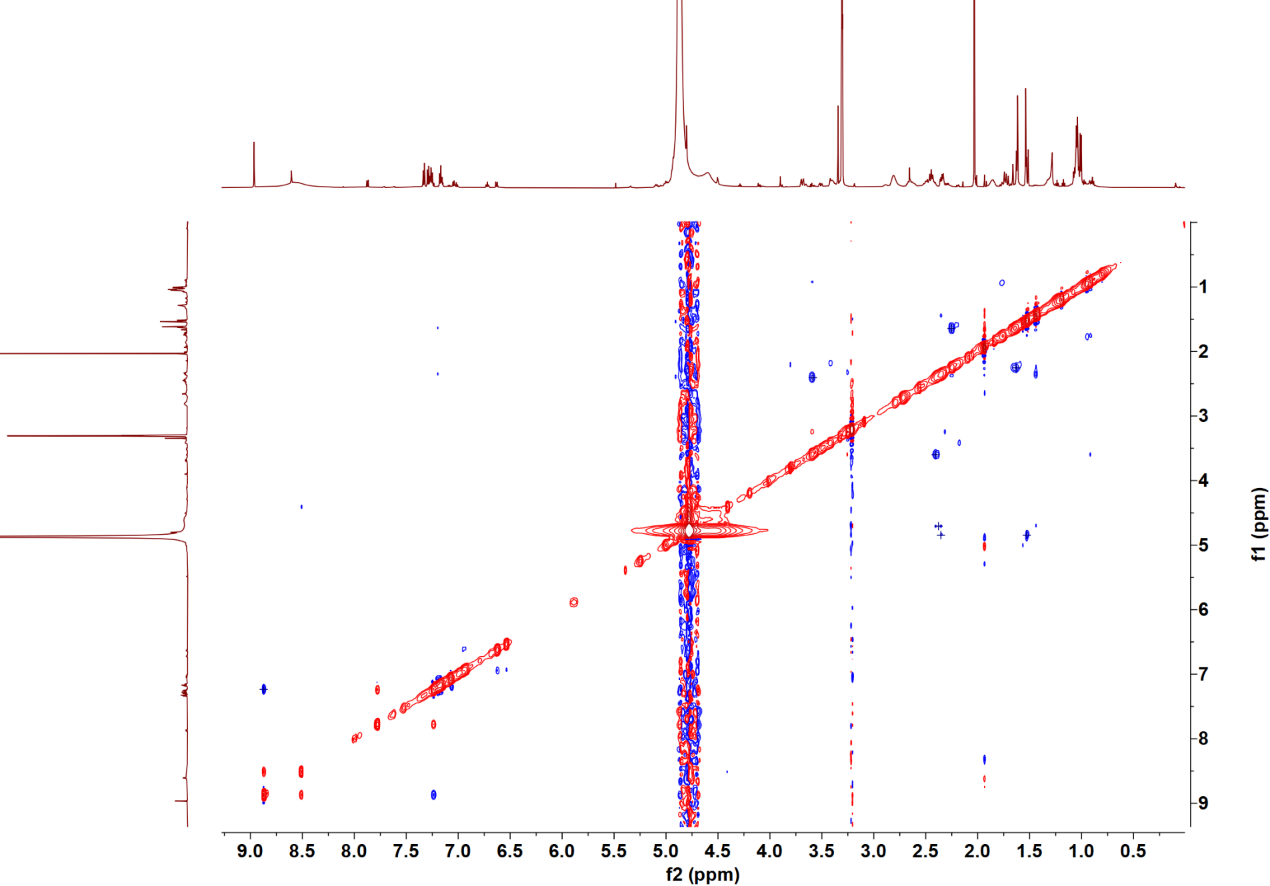


**Figure S74**. NOESY spectrum of compound **9** in CD_3_OD (600 MHz)


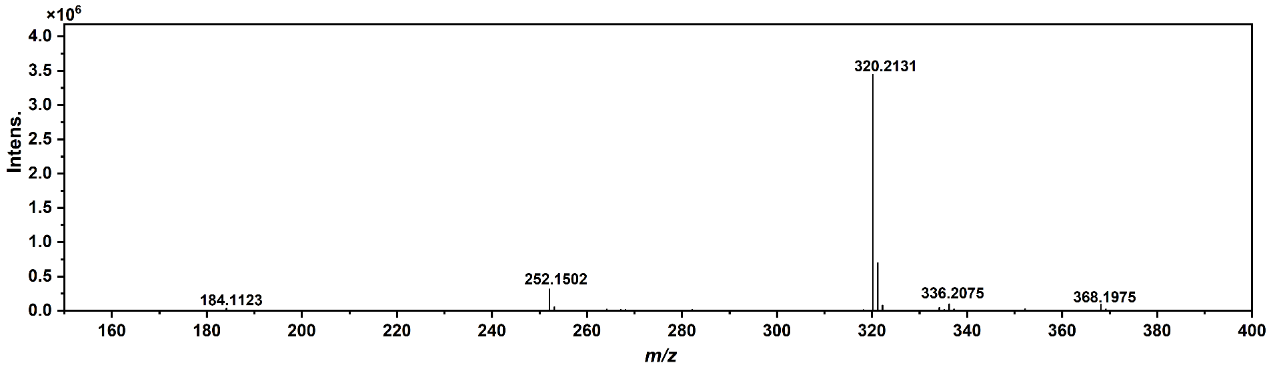


**Figure S75**. HRESIMS spectrum of compound **10**


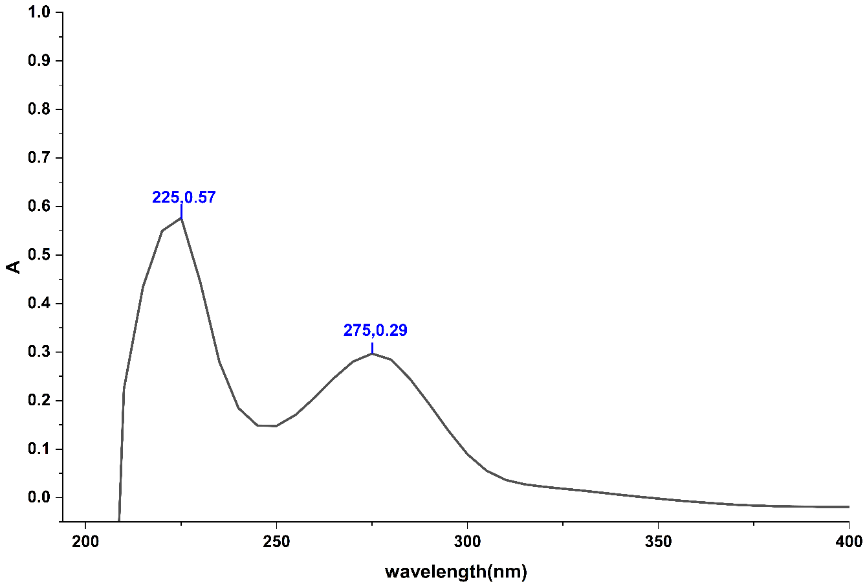


**Figure S76**. UV spectrum of compound **10**


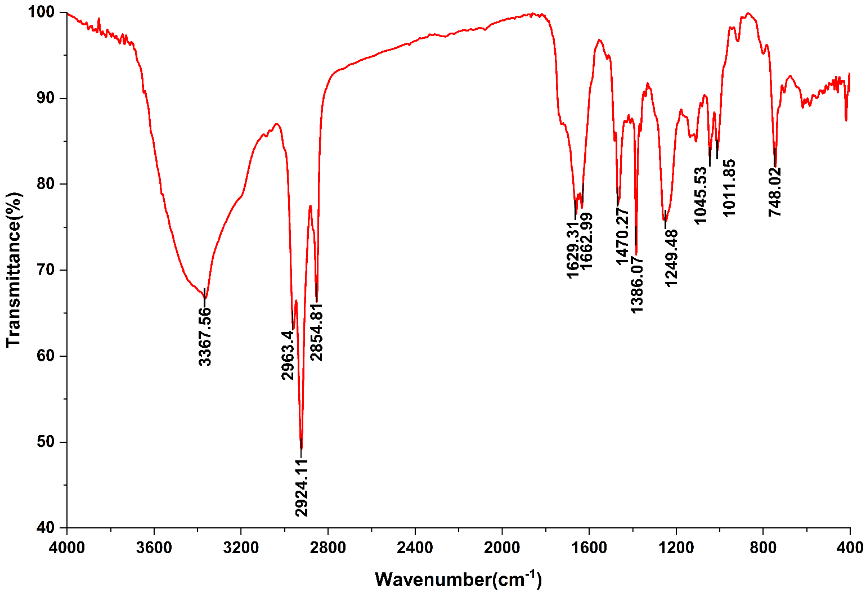


**Figure S77**. IR spectrum of compound **10**


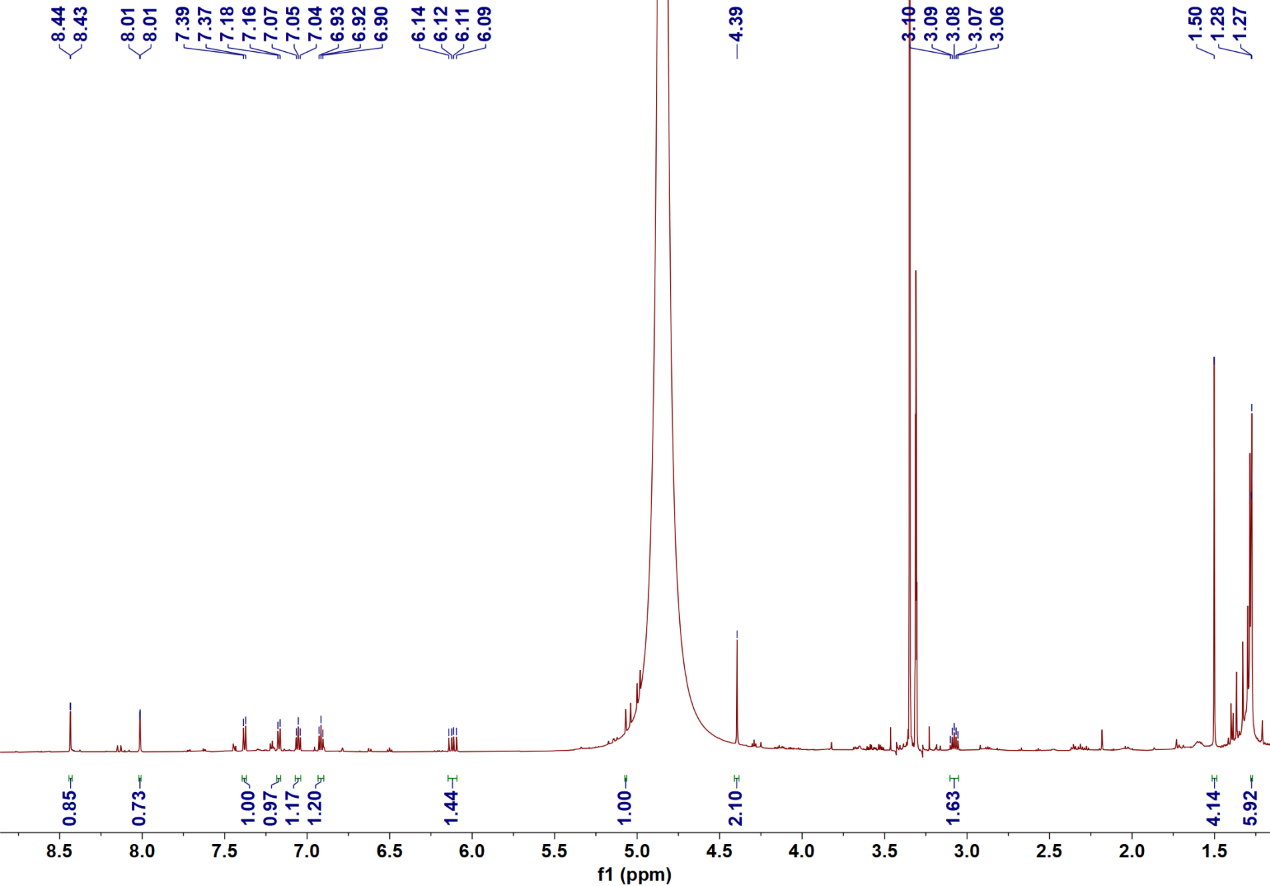


**Figure S78.** ^1^H NMR spectrum of compound **10** in CD_3_OD (600 MHz)


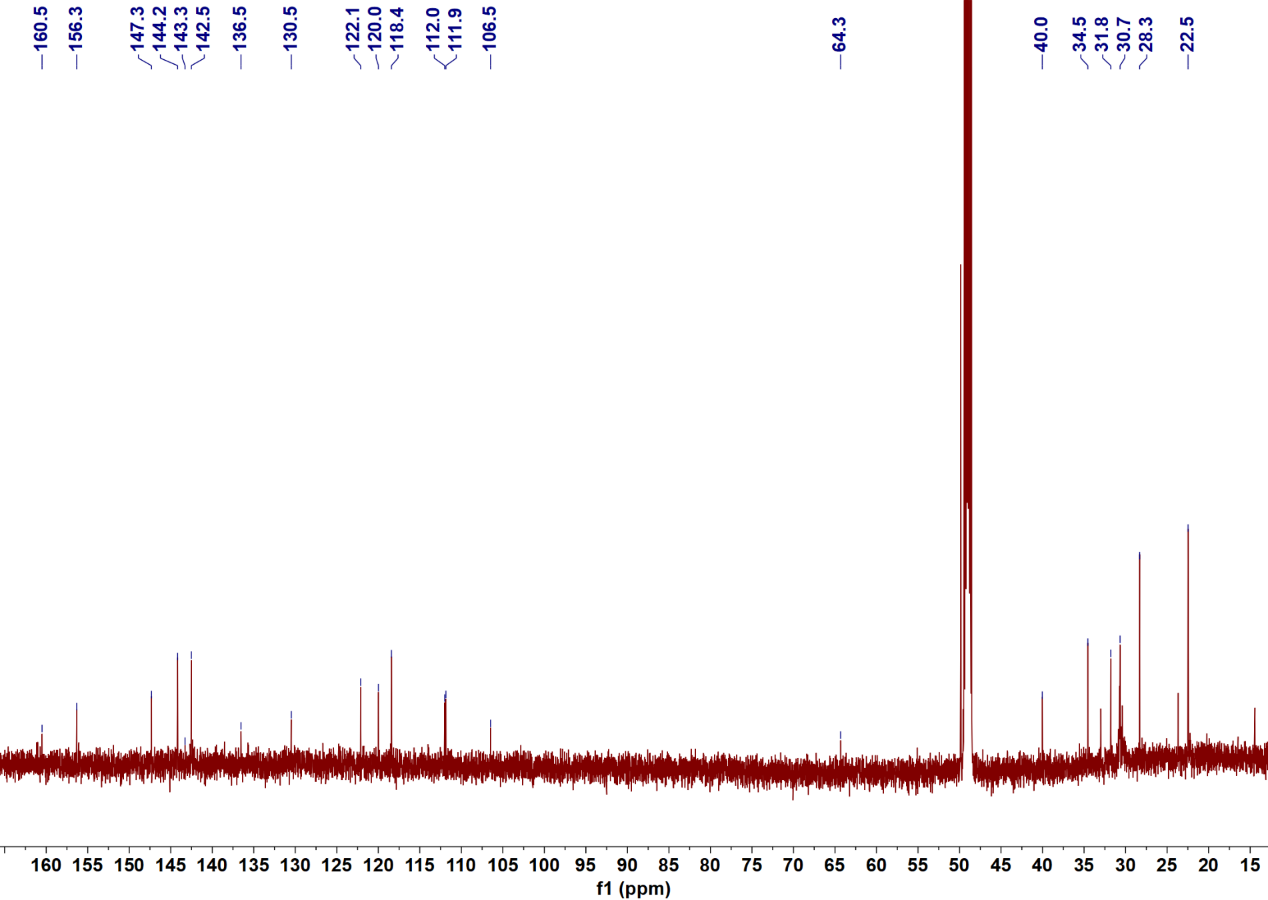


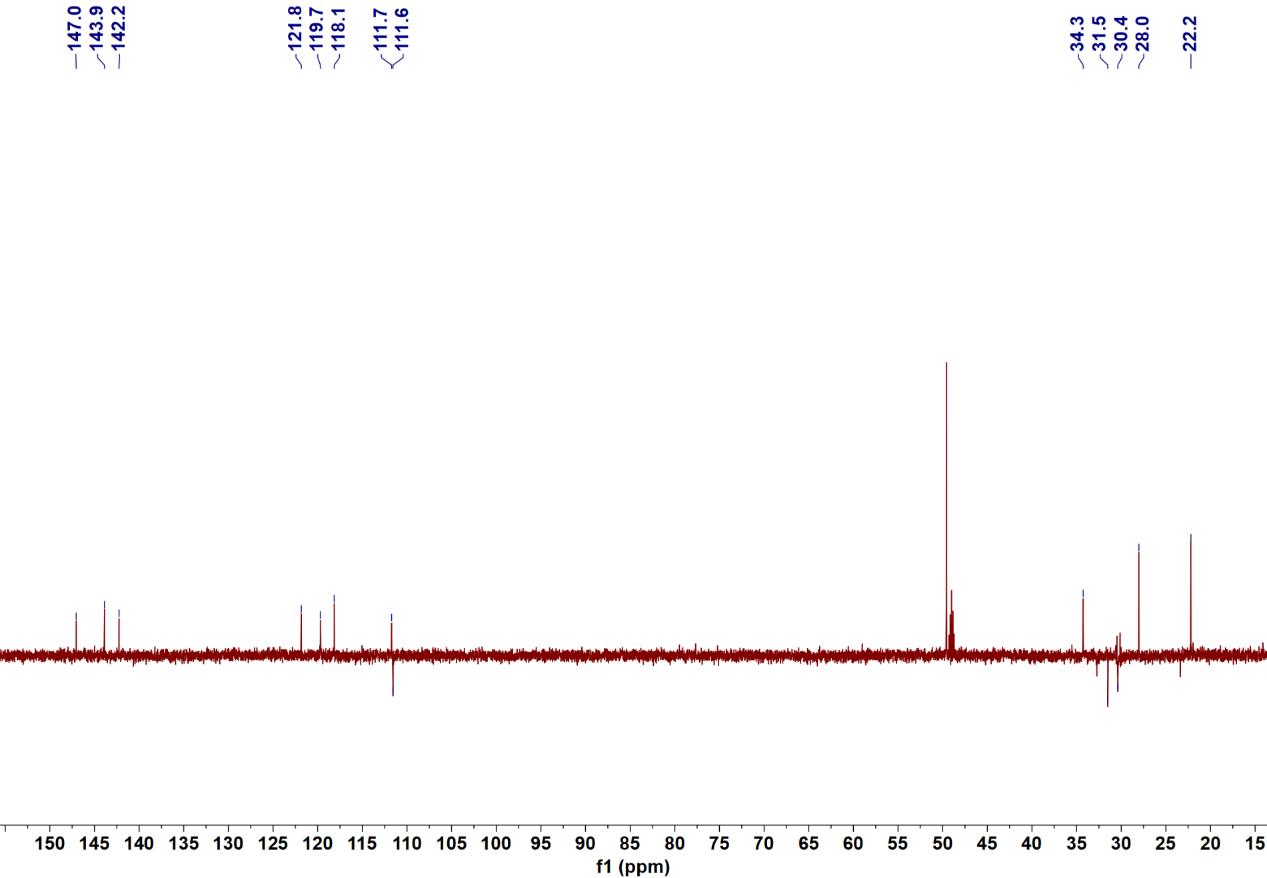


**Figure S79.** ^13^C NMR spectrum of compound **10** in CD_3_OD (150 MHz)


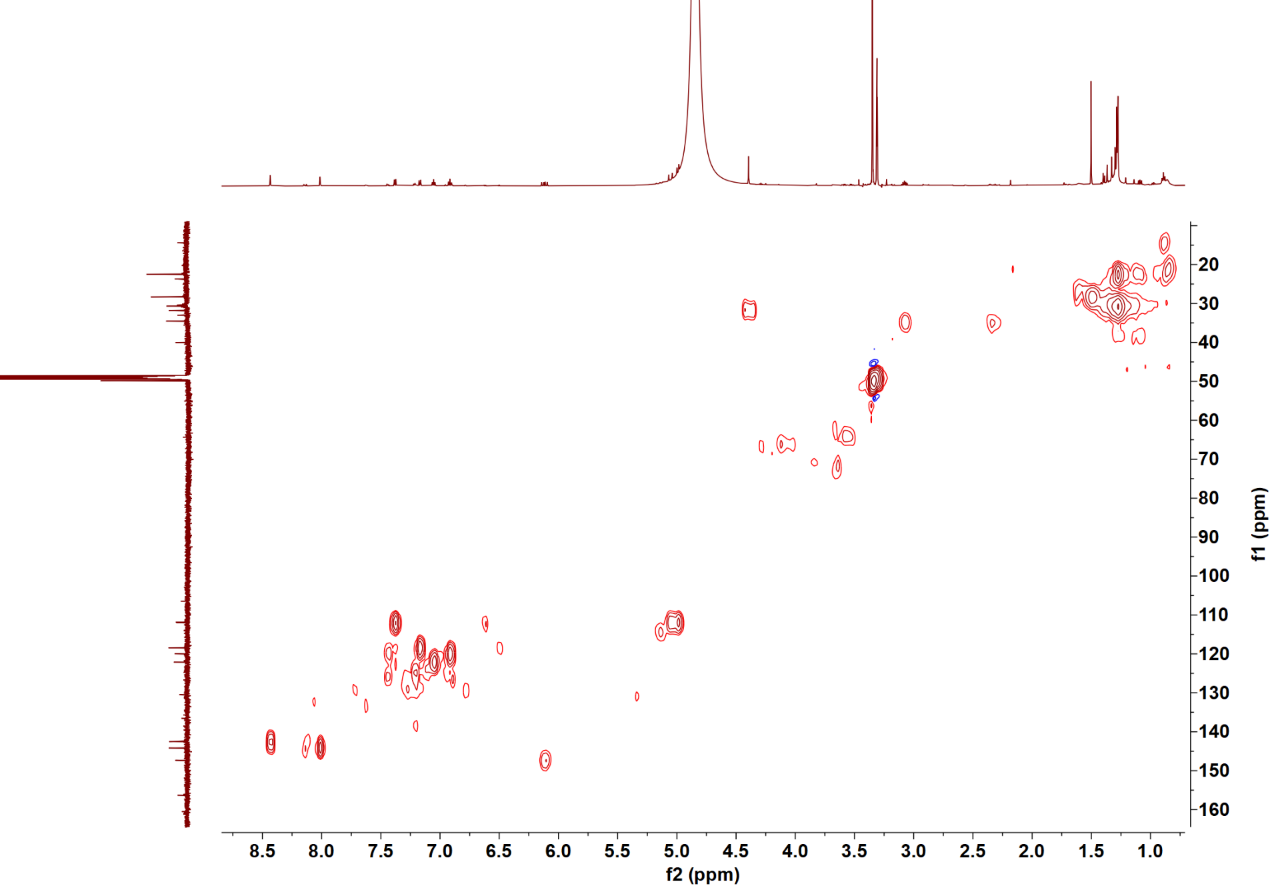


**Figure S80.** HSQC spectrum of compound **10** in CD_3_OD (600 MHz)


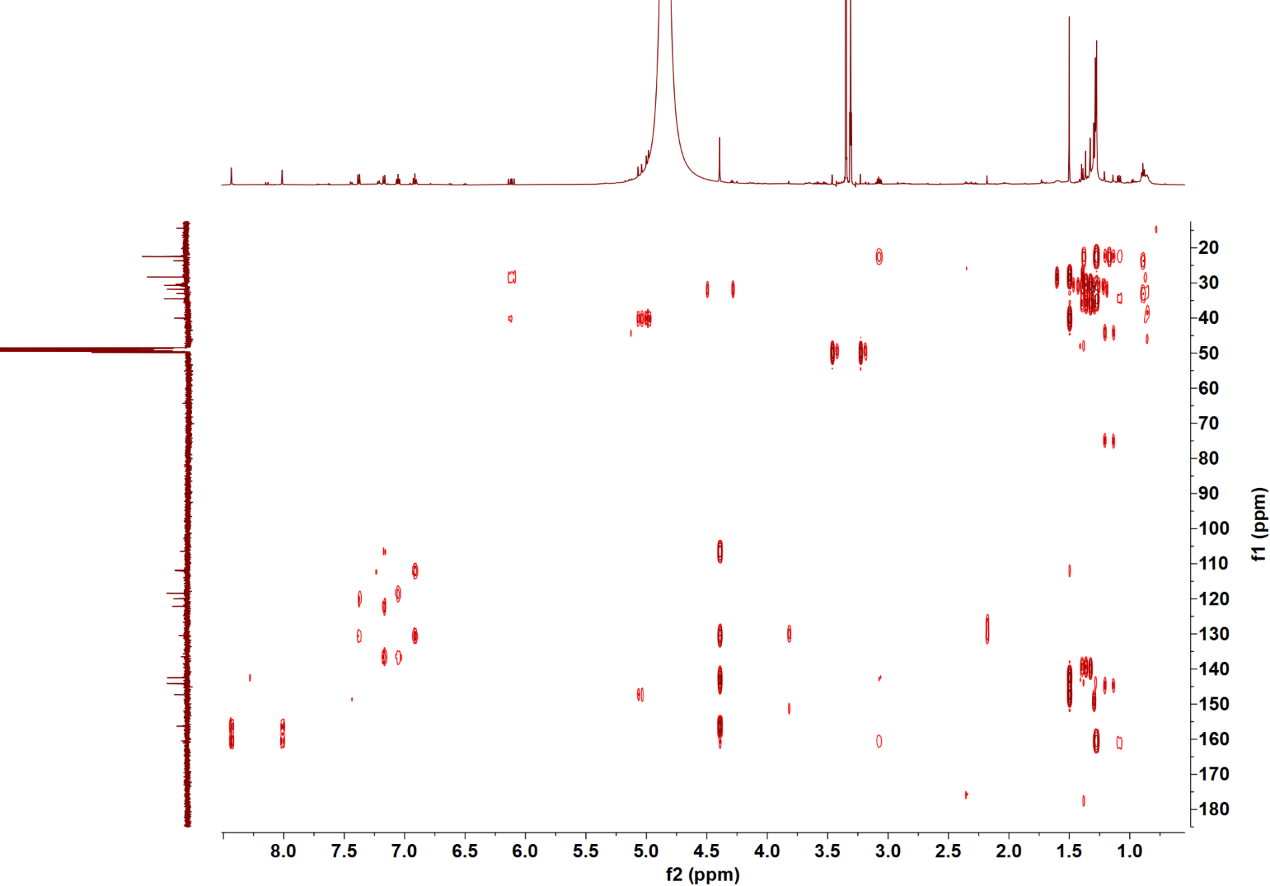


**Figure S81.** HMBC spectrum of compound **10** in CD_3_OD (600 MHz)


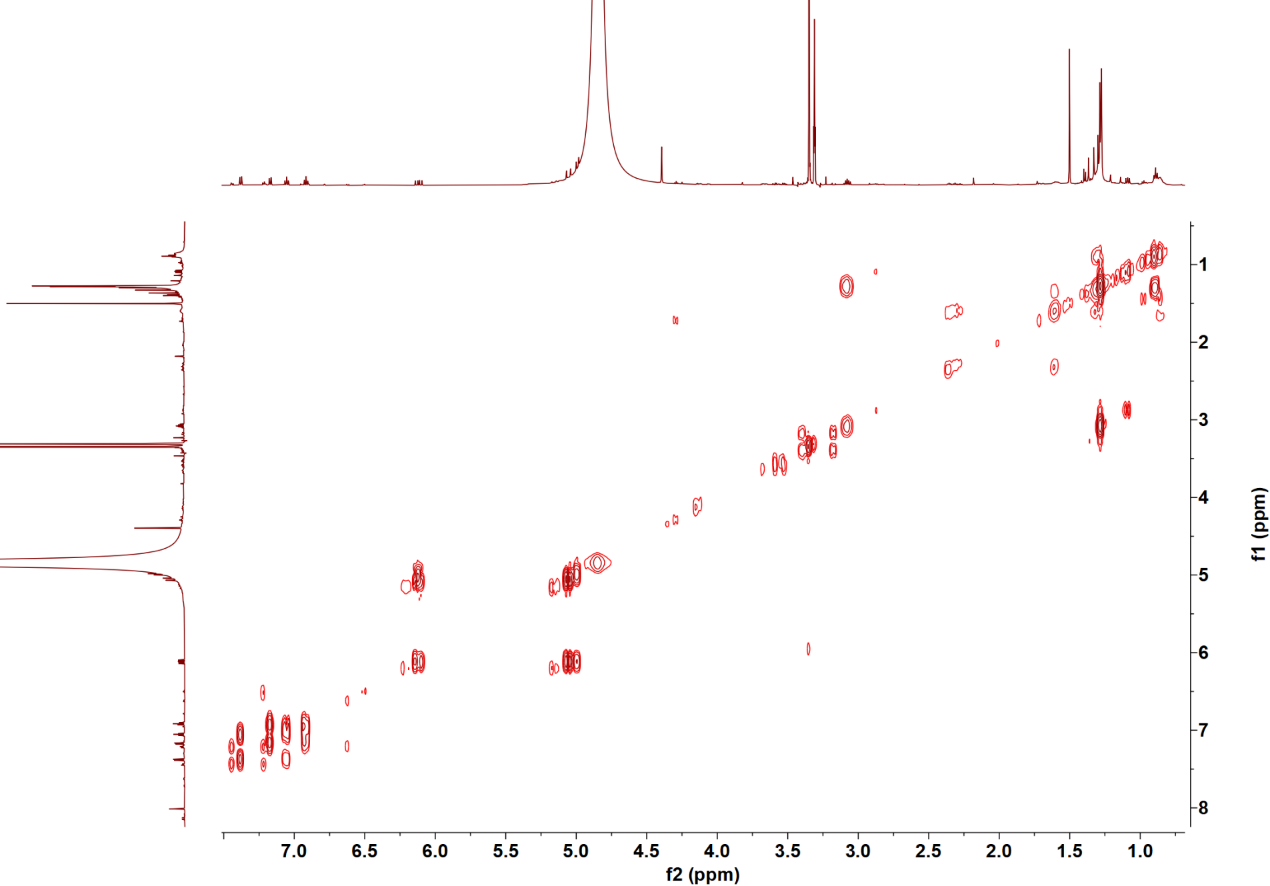


**Figure S82**. ^1^H-^1^H COSY spectrum of compound **10** in CD_3_OD (600 MHz)


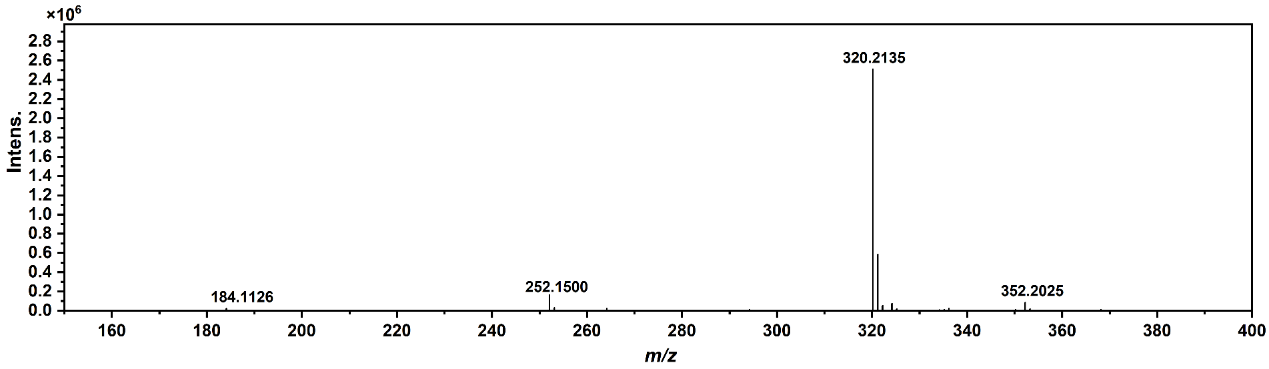


**Figure S83**. HRESIMS spectrum of compound **11**


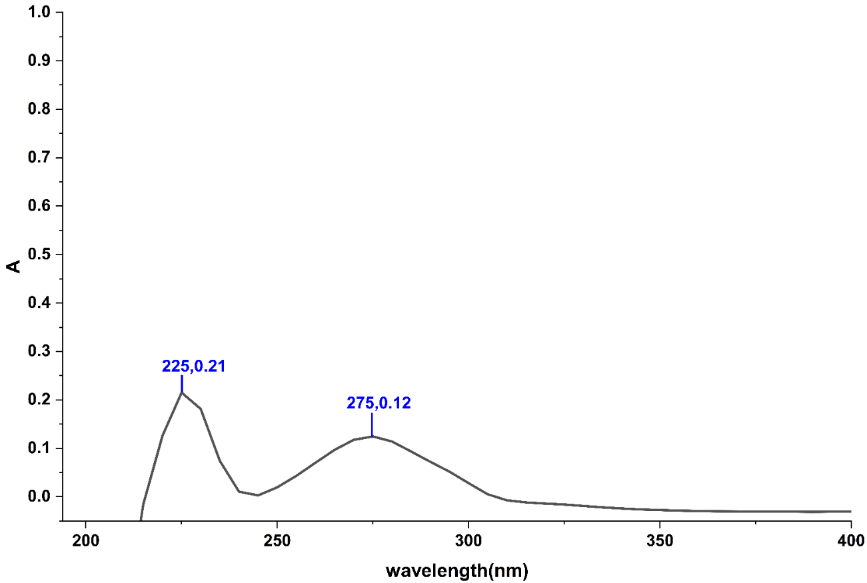


**Figure S84**. UV spectrum of compound **11**


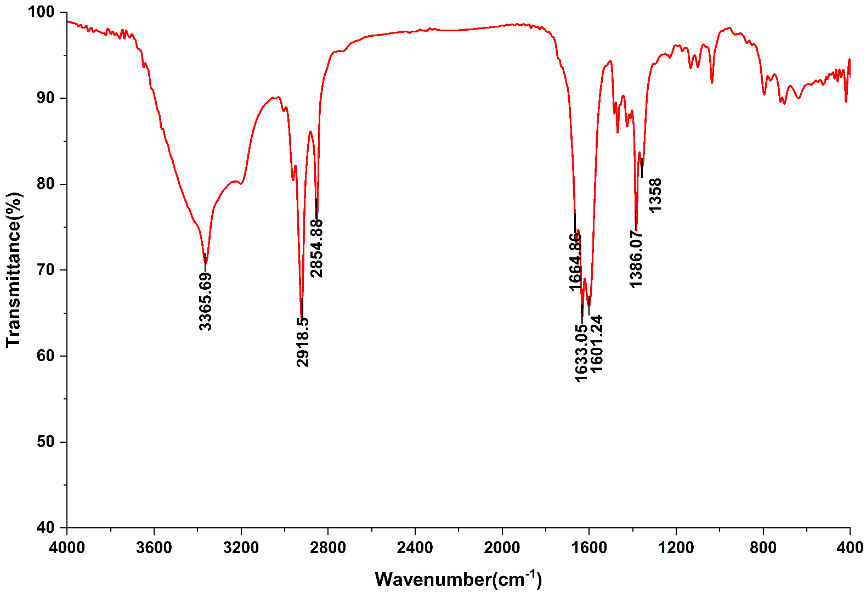


**Figure S85**. IR spectrum of compound **11**


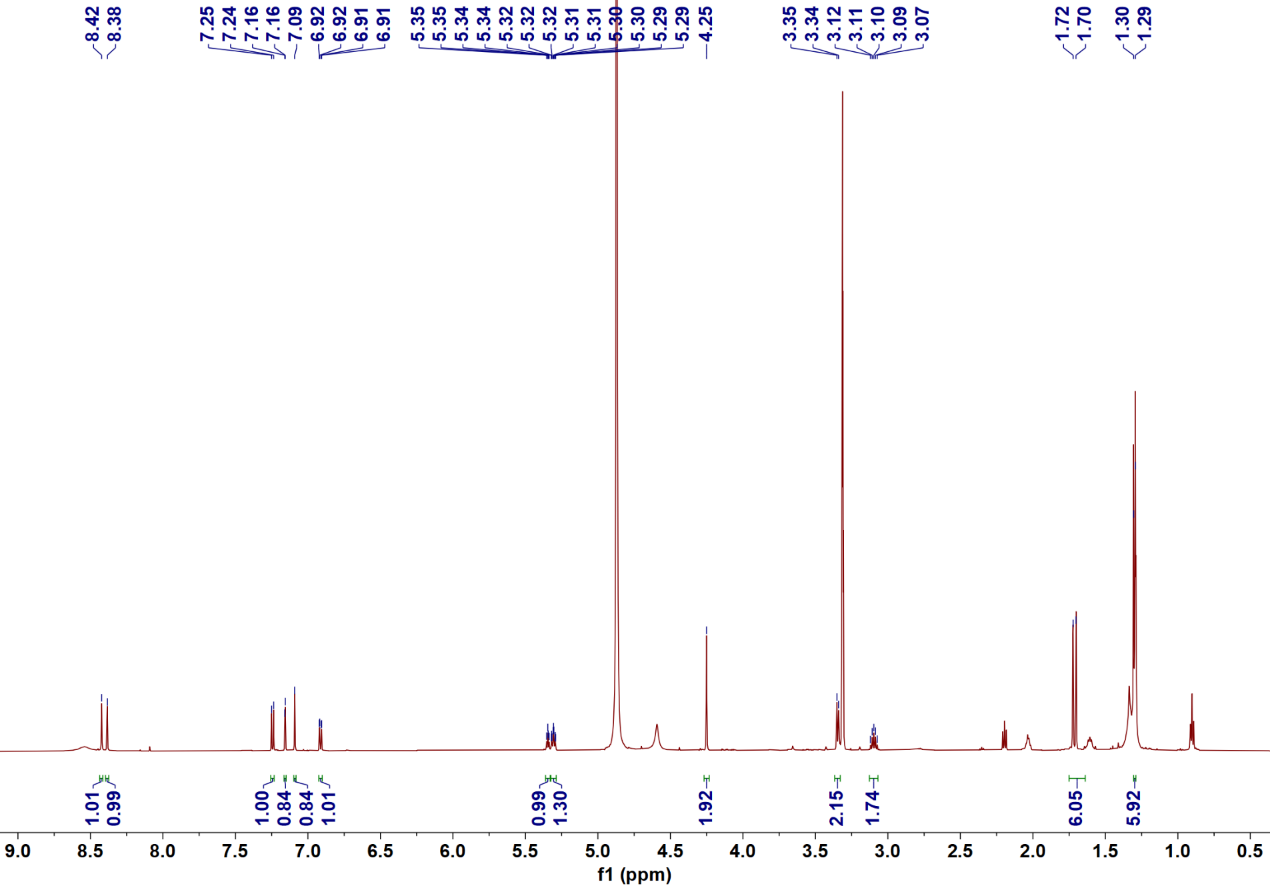


**Figure S86.** ^1^H NMR spectrum of compound **11** in CD_3_OD (600 MHz)


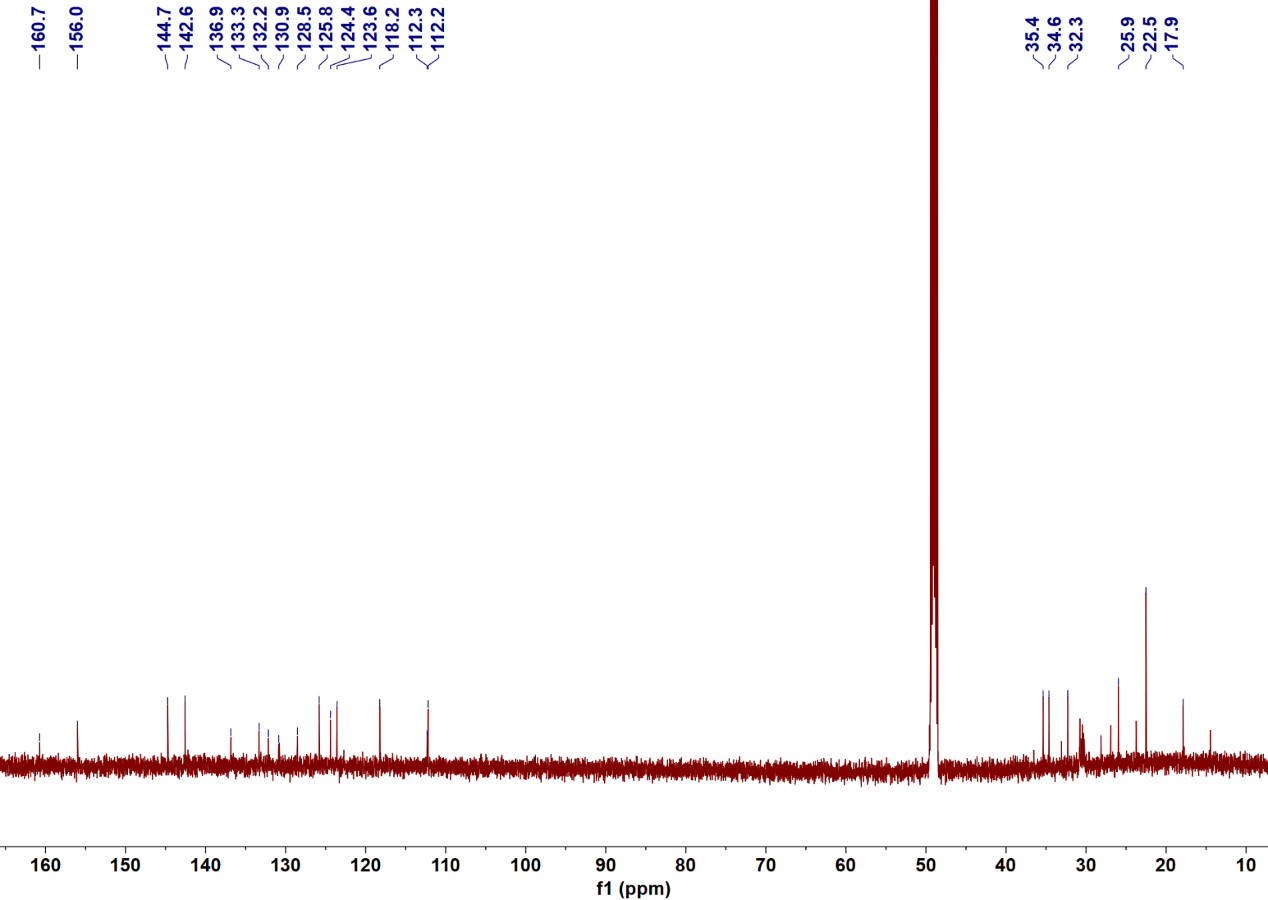


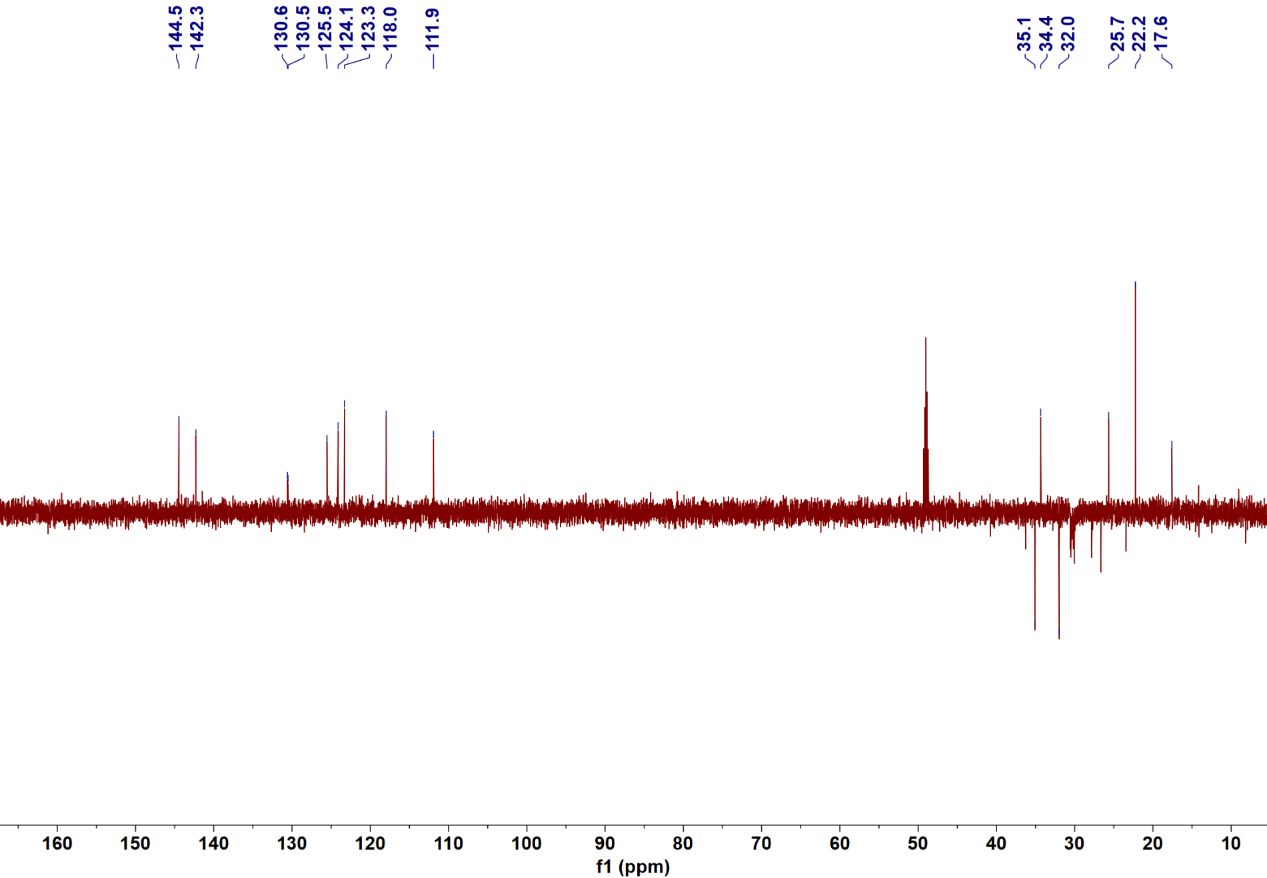


**Figure S87.** ^13^C NMR spectrum of compound **11** in CD_3_OD (150 MHz)


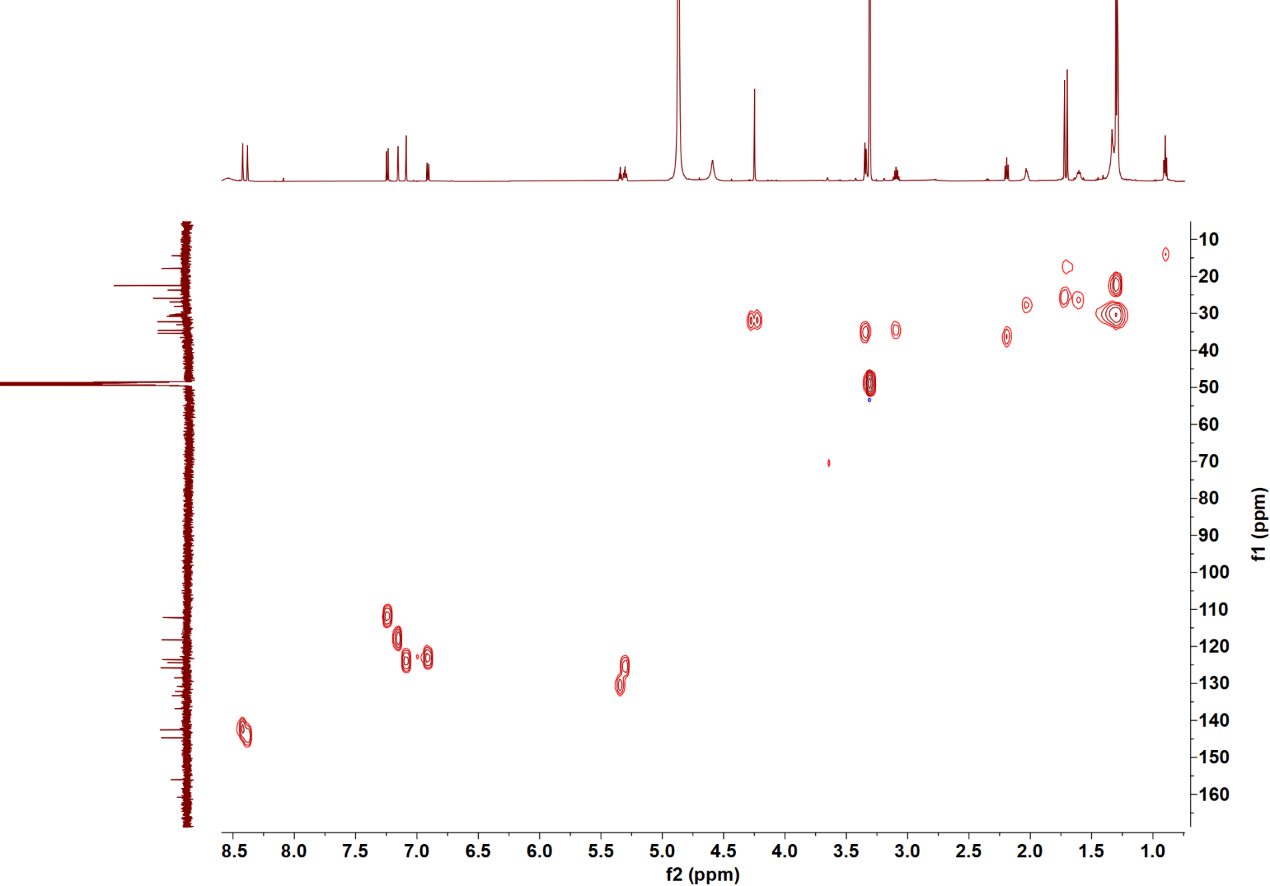


**Figure S88.** HSQC spectrum of compound **11** in CD_3_OD (600 MHz)


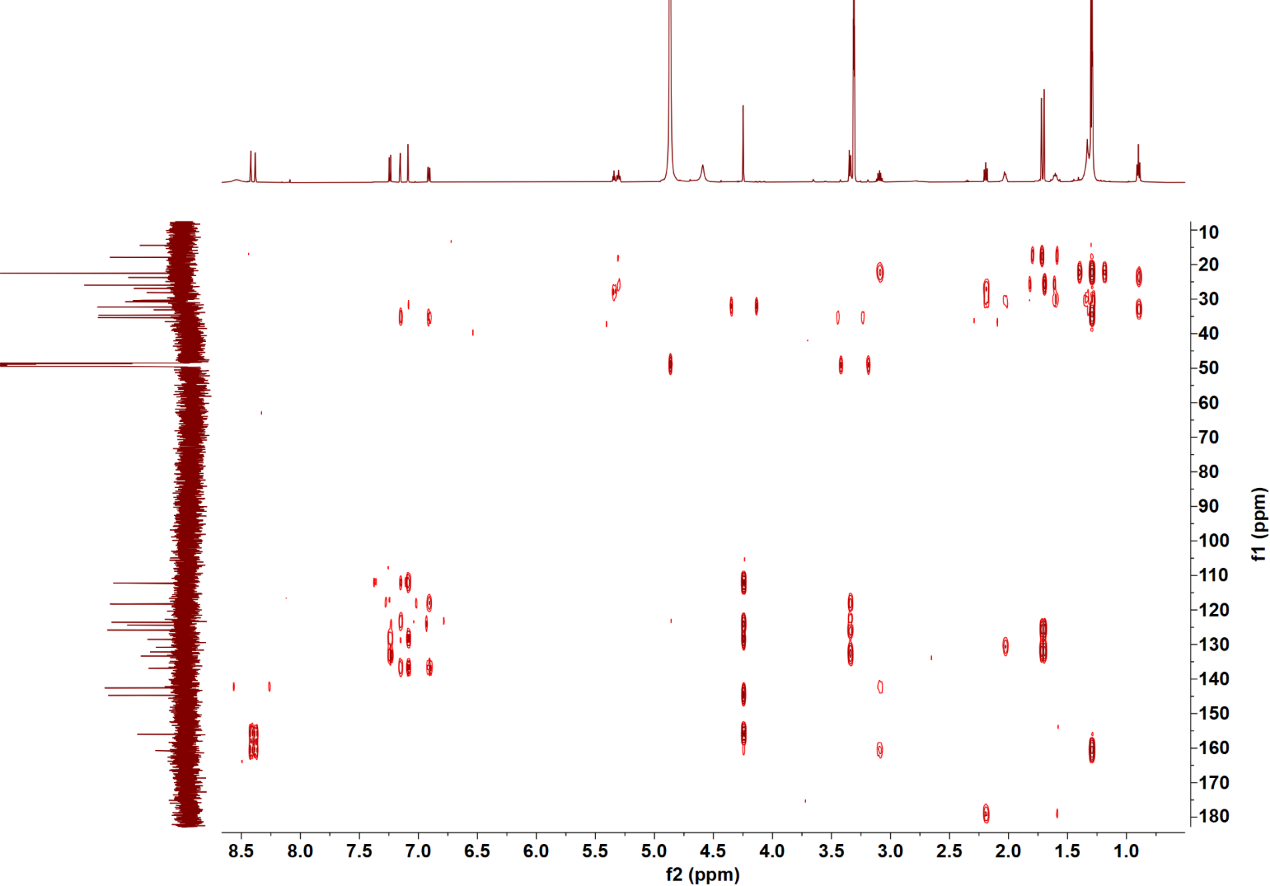


**Figure S89.** HMBC spectrum of compound **11** in CD_3_OD (600 MHz)


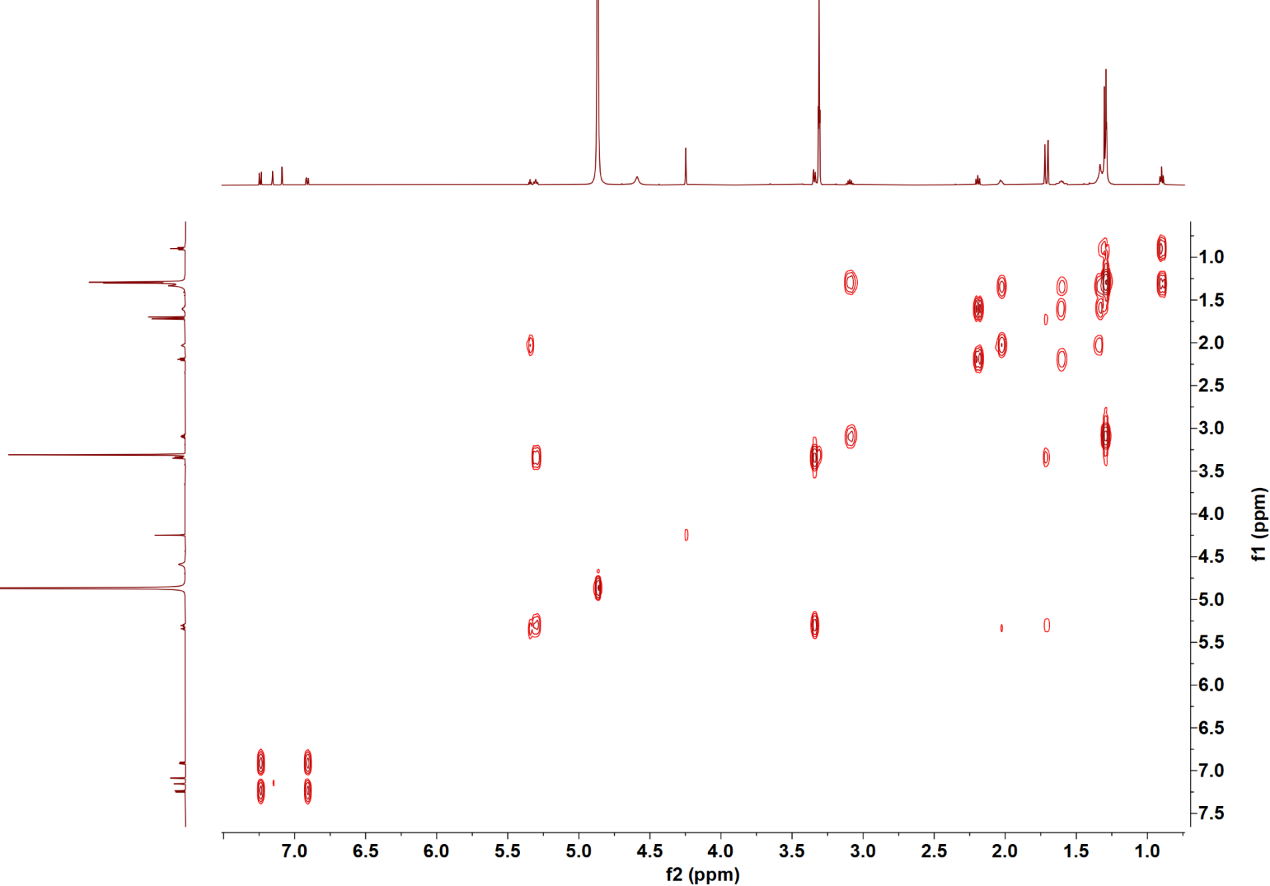


**Figure S90**. ^1^H-^1^H COSY spectrum of compound **11** in CD_3_OD (600 MHz)


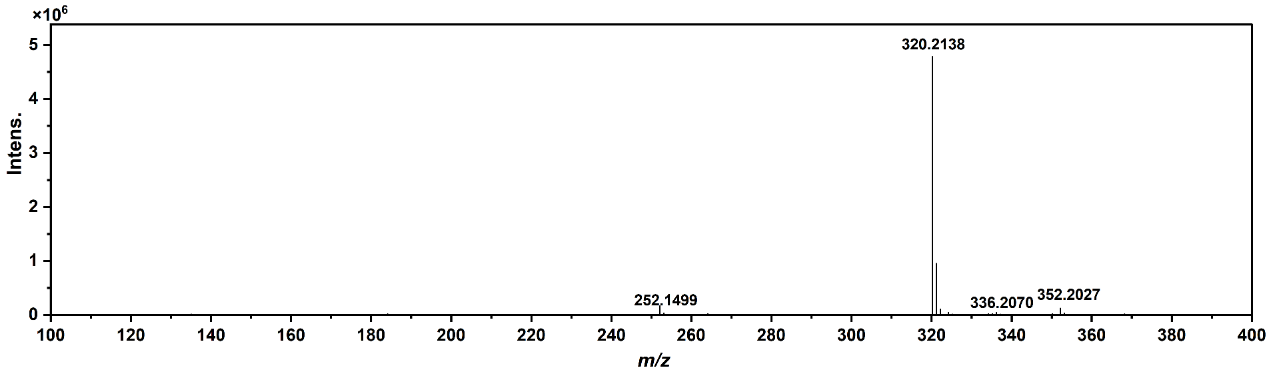


**Figure S91**. HRESIMS spectrum of compound **12**


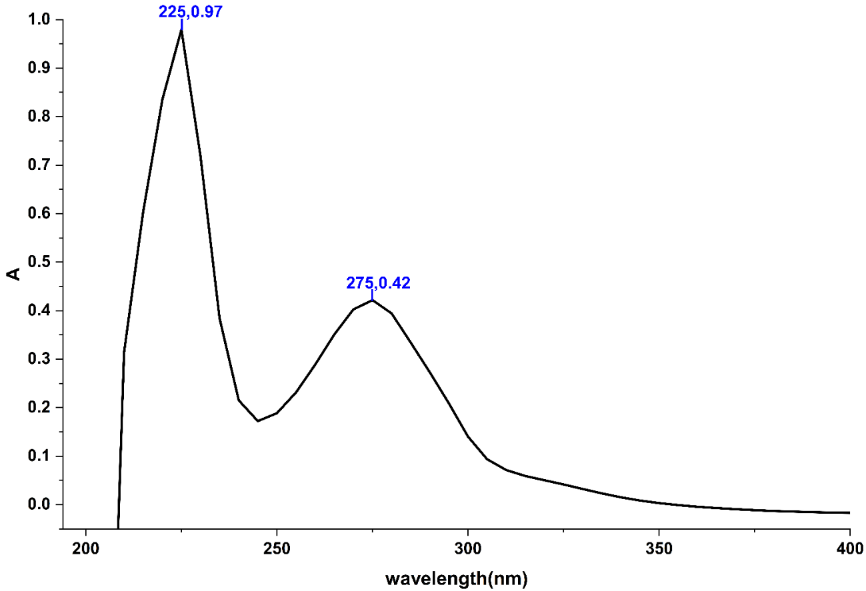


**Figure S92**. UV spectrum of compound **12**


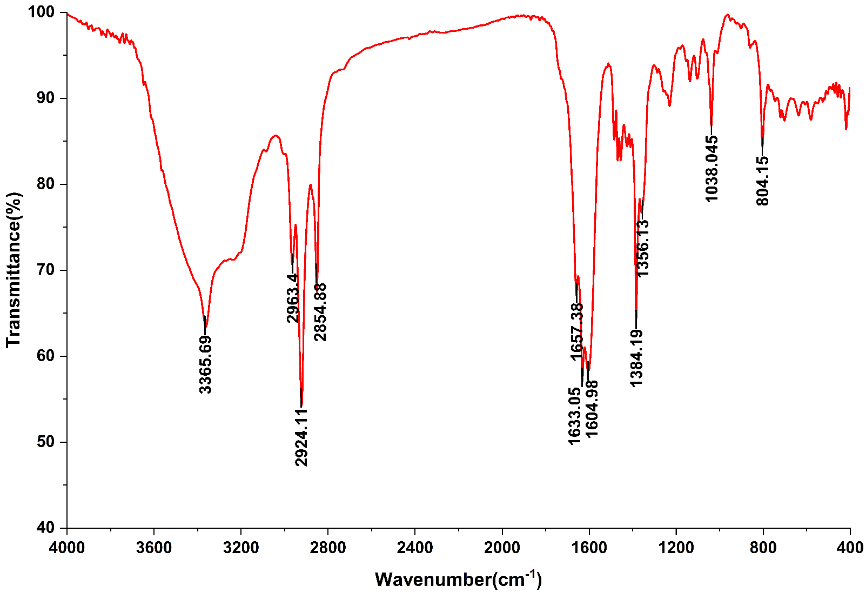


**Figure S93**. IR spectrum of compound **12**


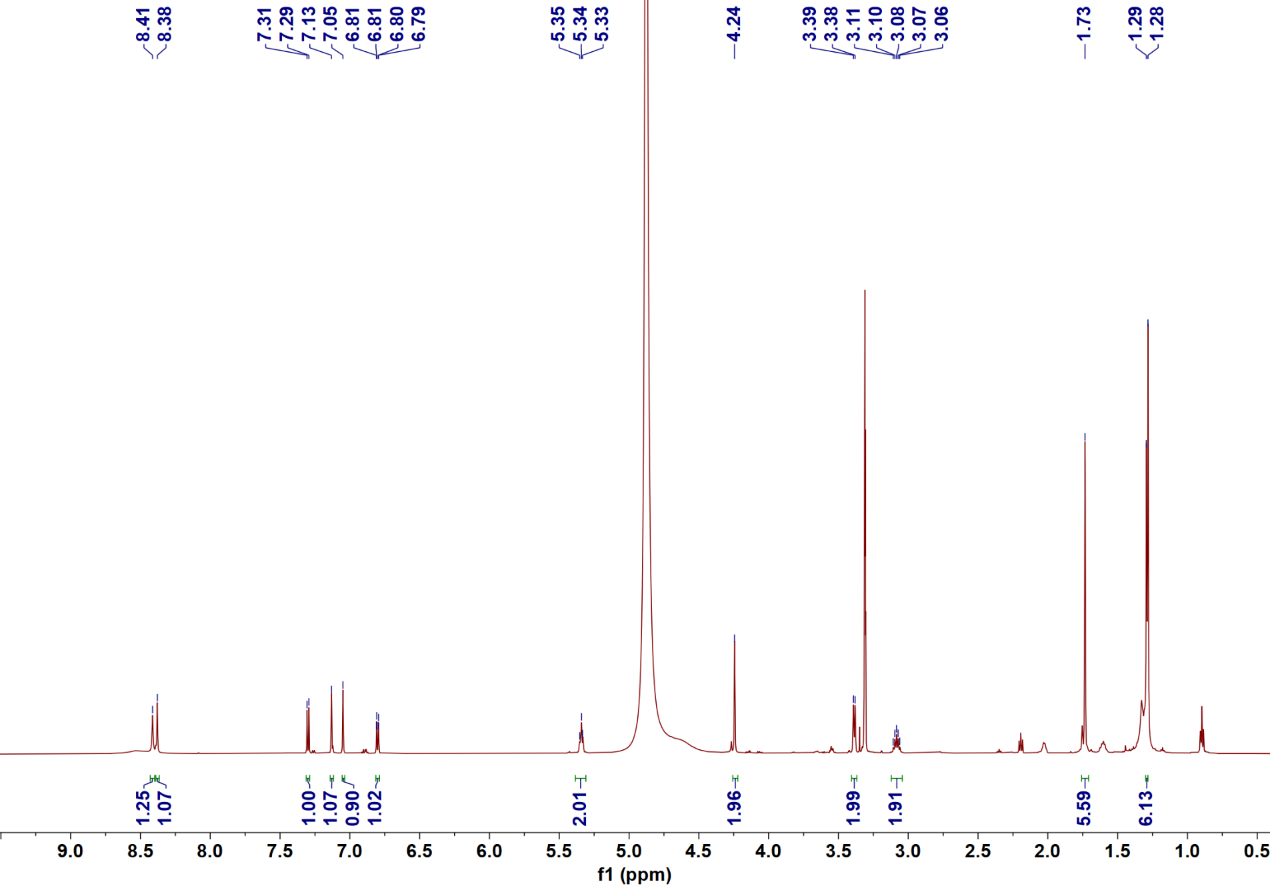


**Figure S94.** ^1^H NMR spectrum of compound **12** in CD_3_OD (600 MHz)


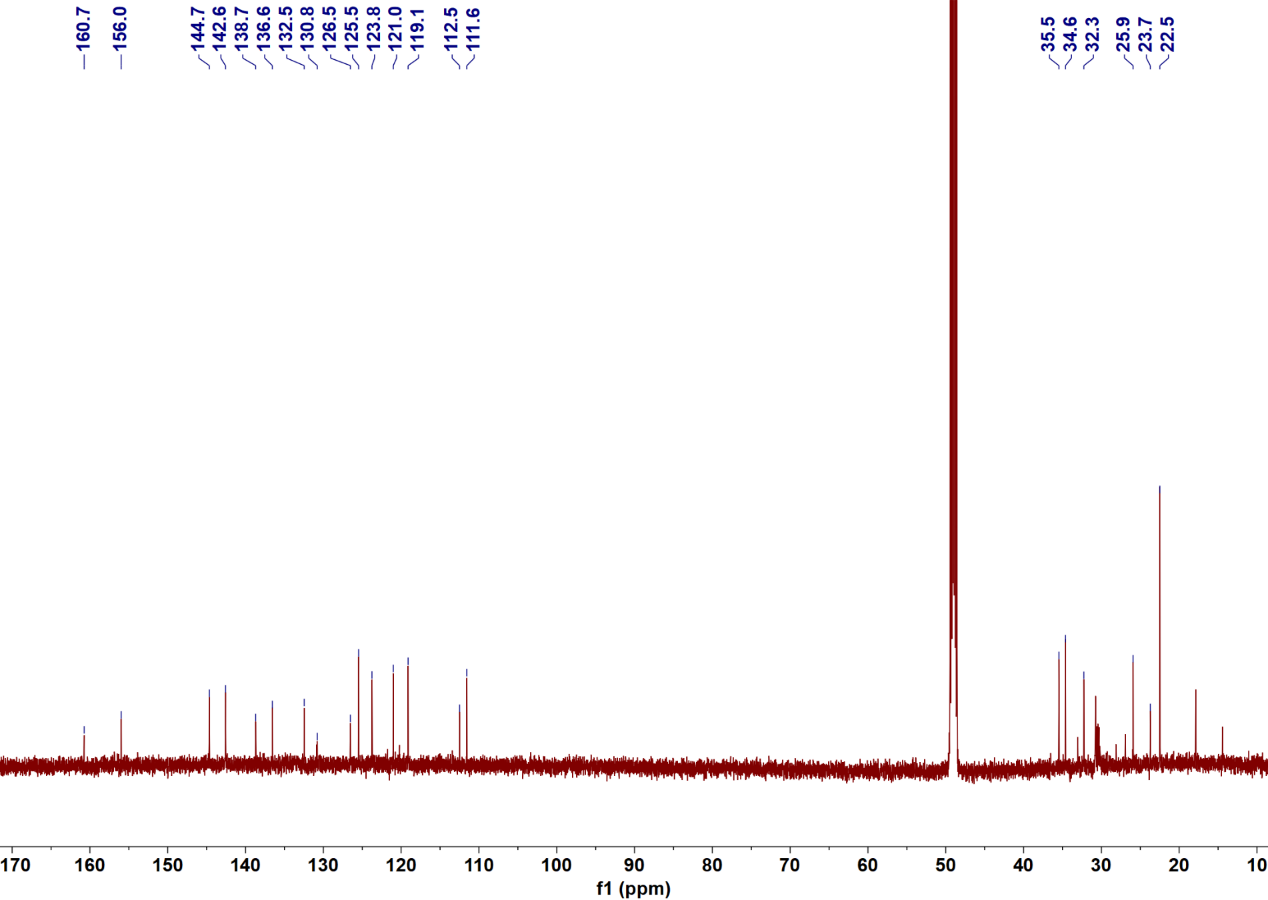


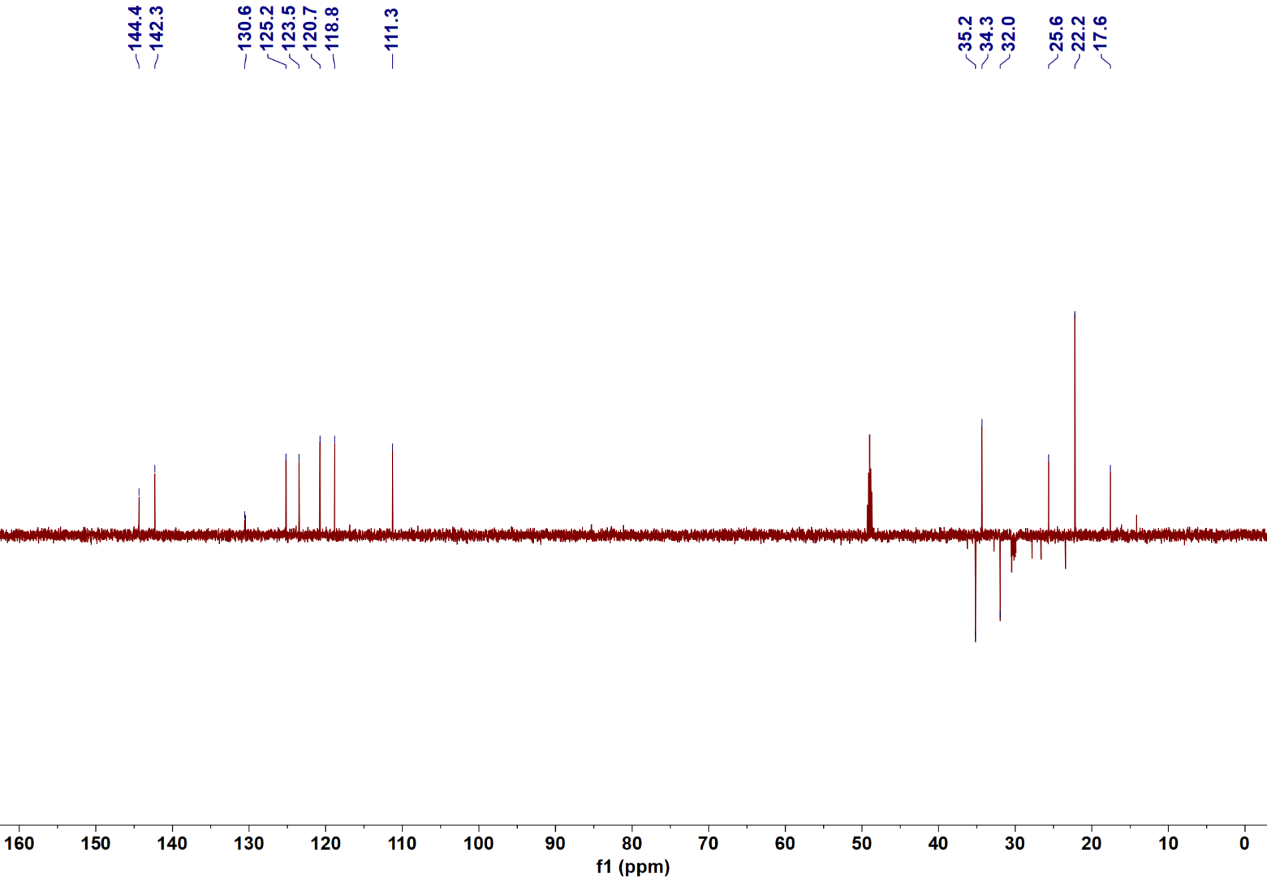


**Figure S95.** ^13^C NMR spectrum of compound **12** in CD_3_OD (150 MHz)


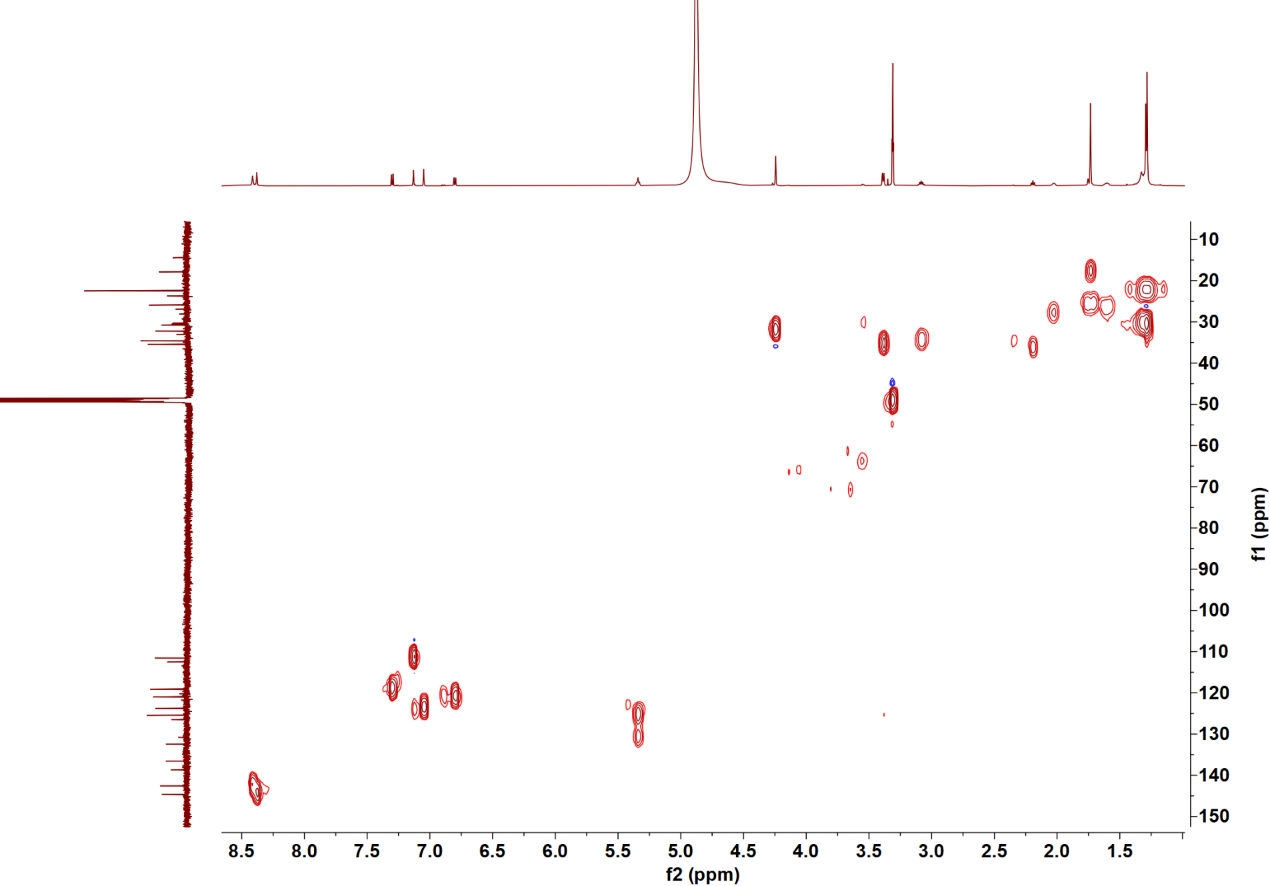


**Figure S96.** HSQC spectrum of compound **12** in CD_3_OD (600 MHz)


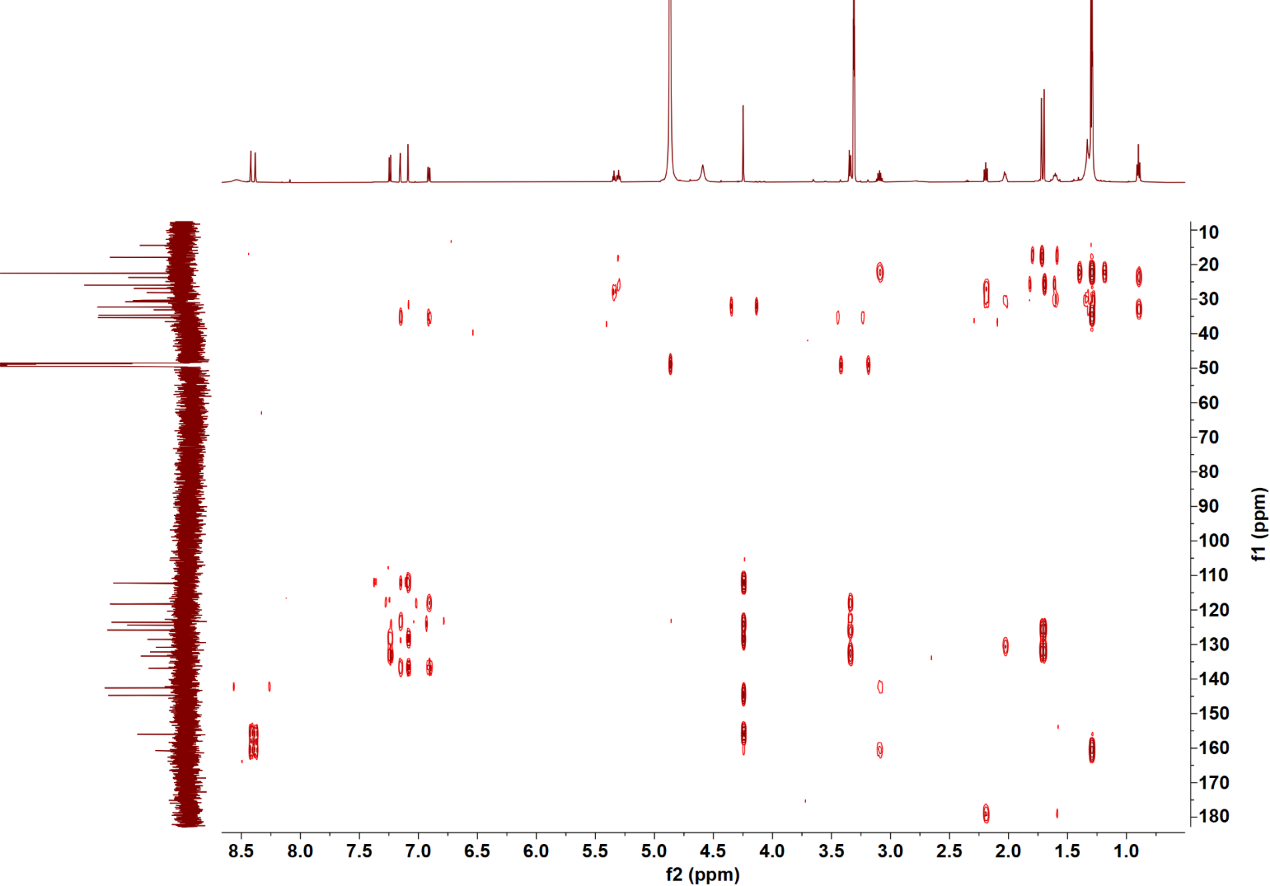


**Figure S97.** HMBC spectrum of compound **12** in CD_3_OD (600 MHz)


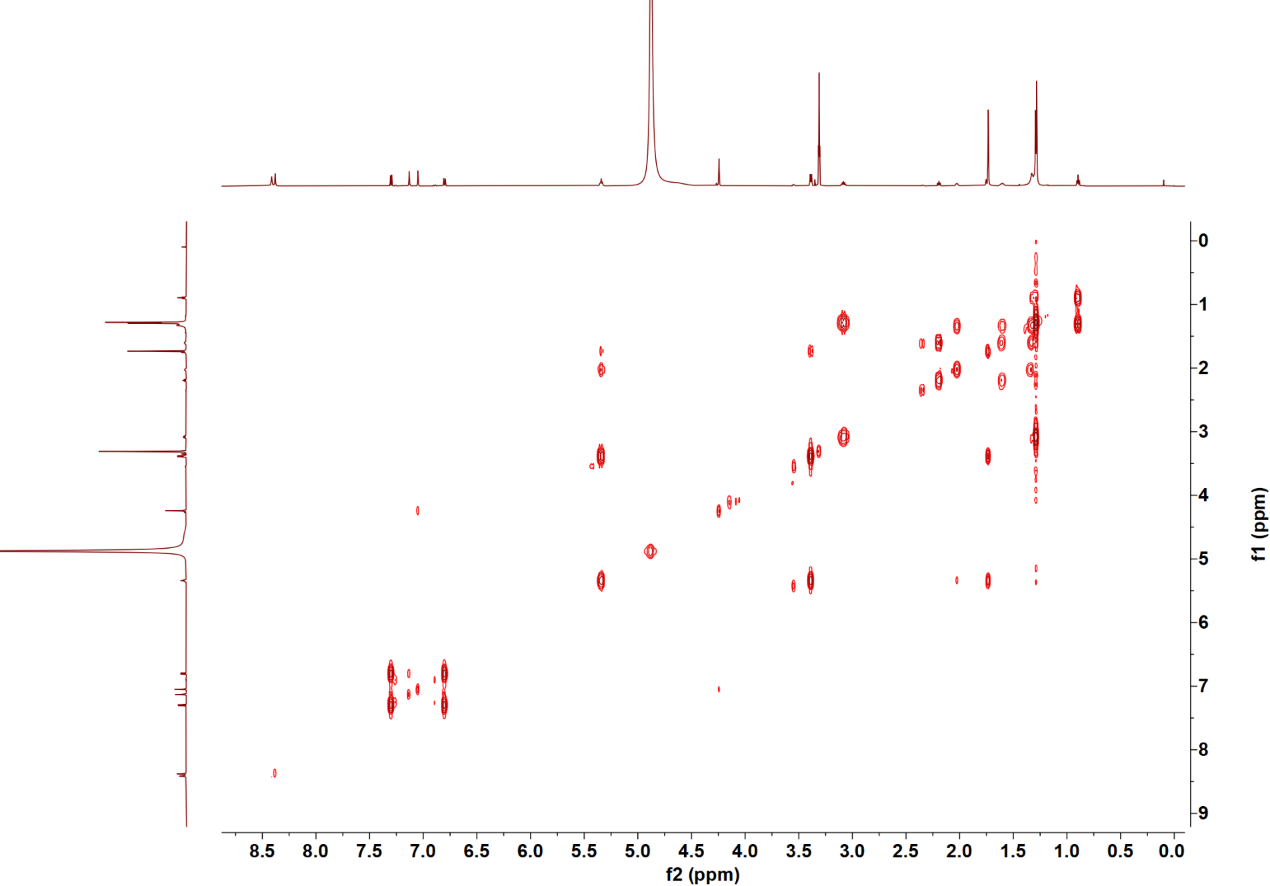


**Figure S98**. ^1^H-^1^H COSY spectrum of compound **12** in CD_3_OD (600 MHz)


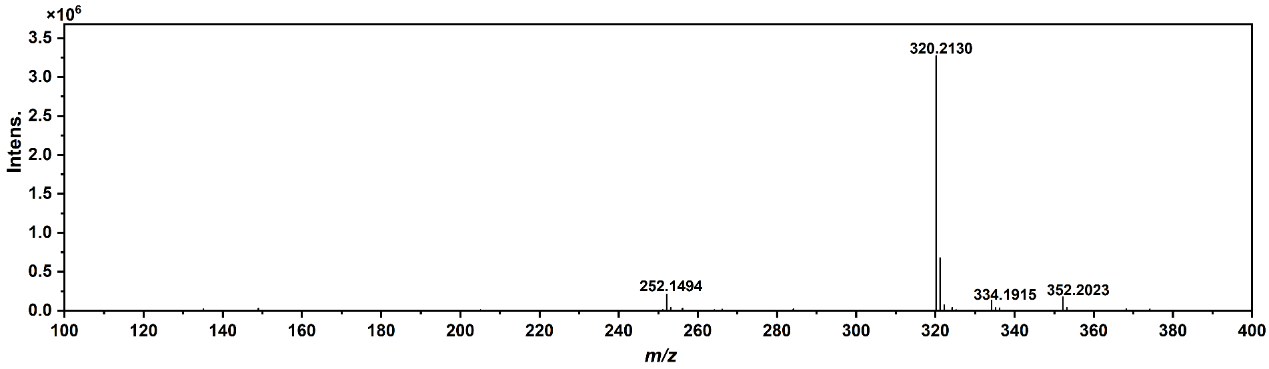


**Figure S99**. HRESIMS spectrum of compound **13**


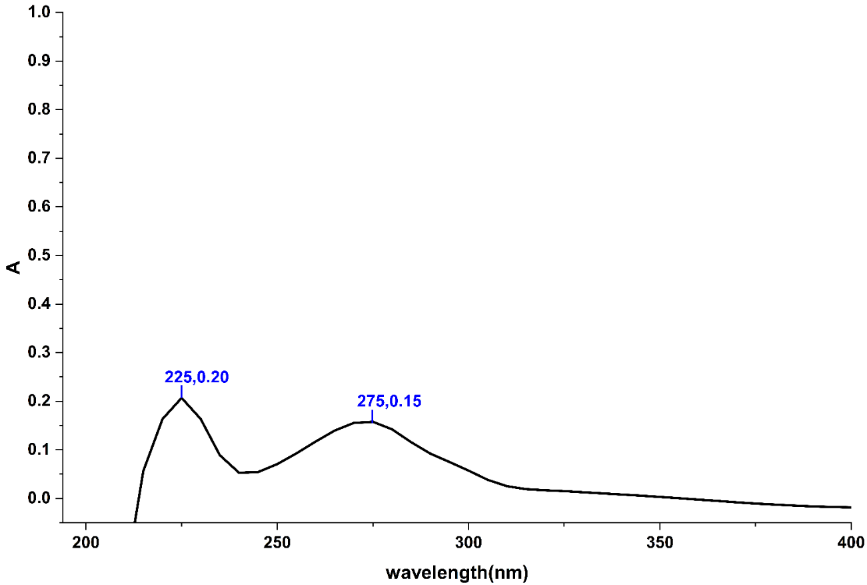


**Figure S100**. UV spectrum of compound **13**

**
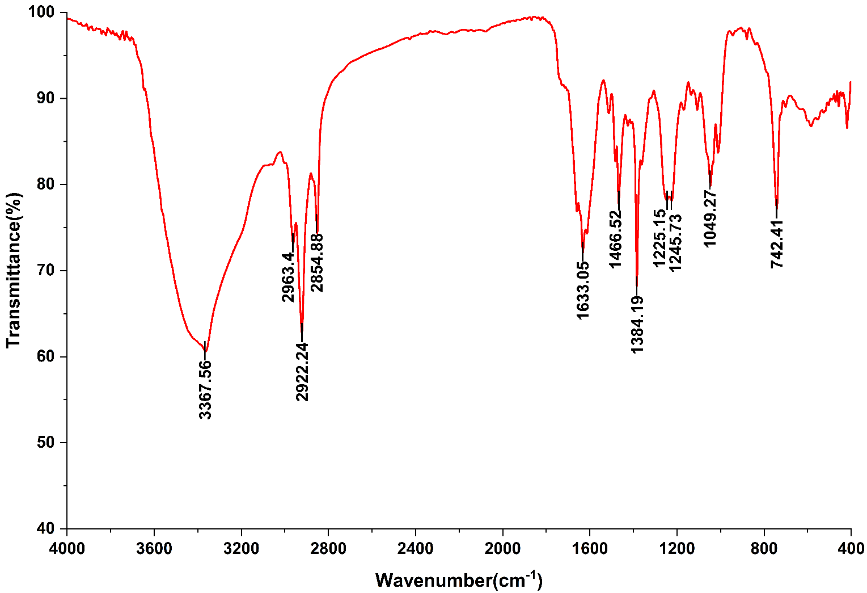
**

**Figure S101**. IR spectrum of compound **13**


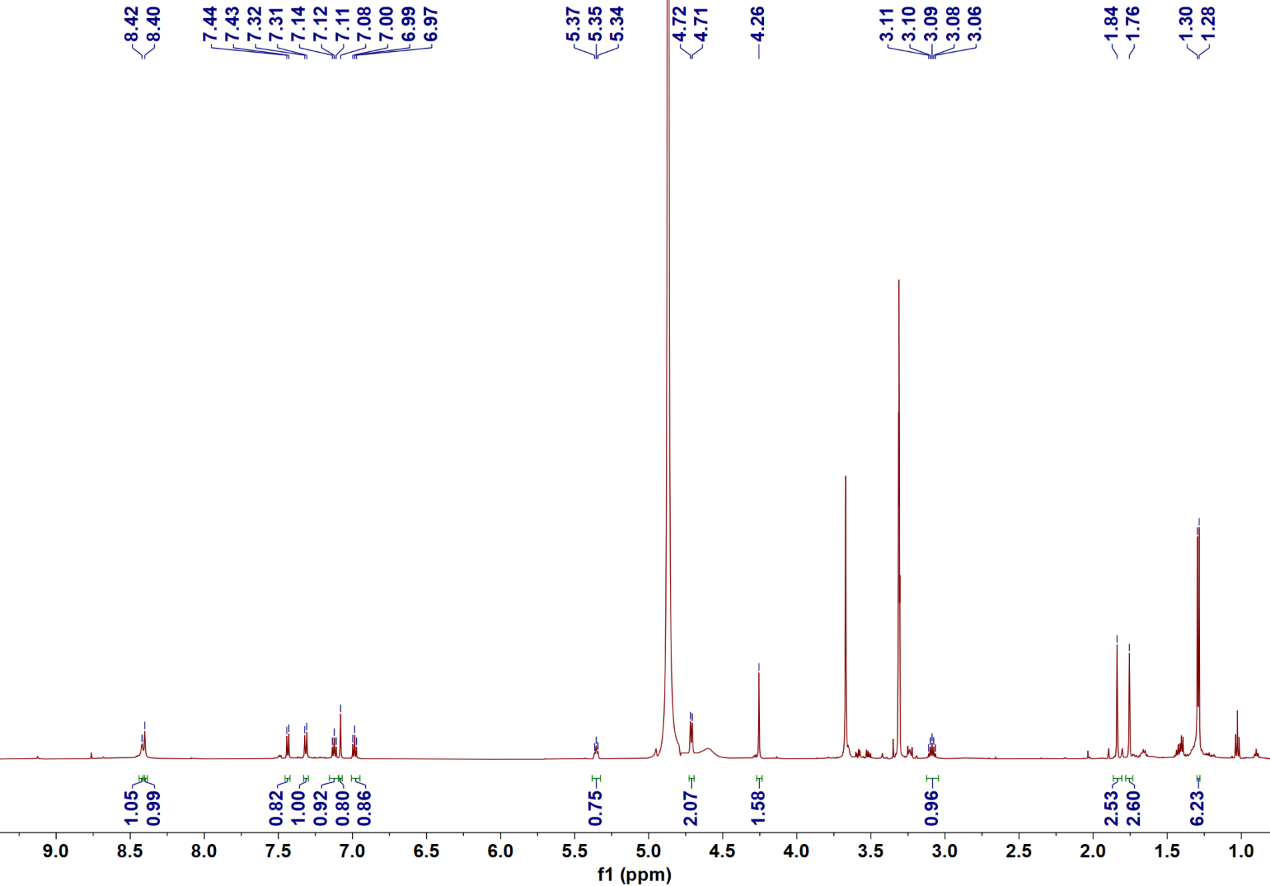


**Figure S102.** ^1^H NMR spectrum of compound **13** in CD_3_OD (600 MHz)

**Figure S103.** ^13^C NMR spectrum of compound **13** in CD_3_OD (150 MHz)

**Figure S104.** HSQC spectrum of compound **13** in CD_3_OD (600 MHz)

**Figure S105.** HMBC spectrum of compound **13** in CD_3_OD (600 MHz)

**Figure S106**. ^1^H-^1^H COSY spectrum of compound **13** in CD_3_OD (600 MHz)

**Figure S107.** ^1^H NMR spectrum of compound **14** in DMSO-*d*_6_ (600 MHz)

**Figure S108.** ^1^H NMR spectrum of compound **15** in DMSO-*d*_6_ (600 MHz)

**Figure S109.** ^13^C NMR spectrum of compound **15** in DMSO-*d*_6_ (150 MHz)

**Figure S110.** HSQC spectrum of compound **15** in DMSO-*d*_6_ (600 MHz)

**Figure S111.** HMBC spectrum of compound **15** in DMSO-*d*_6_ (600 MHz)

**Figure S112**. ^1^H-^1^H COSY spectrum of compound **15** in DMSO-*d*_6_ (600 MHz)

**Figure S113.** ^1^H NMR spectrum of compound **16** in DMSO-*d*_6_ (600 MHz)

**Figure S114.** ^13^C NMR spectrum of compound **16** in DMSO-*d*_6_ (150 MHz)

**Figure S115**. HRESIMS spectrum of compound **17**

**Figure S116**. UV spectrum of compound **17**

**Figure S117**. IR spectrum of compound **17**

**Figure S118.** ^1^H NMR spectrum of compound **17** in CD_3_OD (600 MHz)

**Figure S119.** ^13^C NMR spectrum of compound **17** in CD_3_OD (150 MHz)

**Figure S120.** HSQC spectrum of compound **17** in CD_3_OD (600 MHz)

**Figure S121.** HMBC spectrum of compound **17** in CD_3_OD (600 MHz)

**Figure S122**. ^1^H-^1^H COSY spectrum of compound **17** in CD_3_OD (600 MHz)

**Figure S123**. NOESY spectrum of compound **17** in CD_3_OD (600 MHz)

**Figure S124**. Experimental and calculated ECD spectra of **17** in MeOH. The calculated ECD spectra of the model molecules of **17** at PBE0/def2-TZVP level.

**References**

[1] M. Sato, Y. Ogata, T. Kodani, K. Watanabe, Understanding the Scope of Cytochrome P450-Catalyzed Radical Dimerization of Diketopiperazines. *Biochemistry* **2025**, *64*, 490–497.

[2] H. Bai, P. Cui, C. Zang, S. Li, Enantioselective total synthesis, divergent optimization and preliminary biological evaluation of (indole-*N*-alkyl)-diketopiperazines. *Bioorg. Med. Chem. Lett.* **2019**, *29*, 126718.

[3] R. Wang, A. M. Piggott, Y.-H. Chooi, H. Li, Discovery, bioactivity and biosynthesis of fungal piperazines. *Nat. Prod. Rep.* **2023**, *40*, 387–411.
