# Supplementary material for: Discovery of unprecedented prenylated indole piperazines and pyrazines through cryptic biosynthetic gene cluster heterologous expression
Source: Nat Prod Bioprospect. 2026 Apr 3;16(1):49. doi: 10.1007/s13659-026-00601-7 (PMC13047030; doi:10.1007/s13659-026-00601-7)

checkCIF/PLATON report

Structure factors have been supplied for datablock(s) 20231215_c73-1

THIS REPORT IS FOR GUIDANCE ONLY. IF USED AS PART OF A REVIEW PROCEDURE FOR PUBLICATION, IT SHOULD NOT REPLACE THE EXPERTISE OF AN EXPERIENCED CRYSTALLOGRAPHIC REFEREE.

No syntax errors found. CIF dictionary Interpreting this report

Datablock: 20231215_c73-1

Bond precision: C-C = 0.0016 A Wavelength=1.54184

Cell:	a=5.73607(5)	b=10.90927(7)	c=20.88001(14)
	alpha=90	beta=96.7304(6)	gamma=90
Temperature:	100 K		

	Calculated	Reported
Volume	1297.590(17)	1297.591(16)
Space group	P 21/n	P 1 21/n 1
Hall group	-P 2yn	-P 2yn
Moiety formula	C16 H17 N3	C16 H17 N3
Sum formula	C16 H17 N3	C16 H17 N3
Mr	251.33	251.32
Dx,g cm-3	1.286	1.286
Z	4	4
Mu (mm-1)	0.607	0.607
F000	536.0	536.0
F000'	537.41	
h,k,lmax	7,13,26	6,13,26
Nref	2649	2608
Tmin,Tmax	0.916,0.935	0.454,1.000
Tmin'	0.913	

Correction method= # Reported T Limits: Tmin=0.454 Tmax=1.000
AbsCorr = MULTI-SCAN

Data completeness= 0.985 Theta(max)= 74.325

R(reflections)= 0.0378(2443) wR2(reflections)= 0.0973(2608)

S = 1.050 Npar= 174

The following ALERTS were generated. Each ALERT has the format
test-name_ALERT_alert-type_alert-level.
Click on the hyperlinks for more details of the test.

Alert level G

```
PLAT007_ALERT_5_G Number of Unrefined Donor-H Atoms .............          1 Report
            H3
PLAT142_ALERT_4_G s.u. on b - Axis Small or Missing .............    0.00007 Ang.
PLAT143_ALERT_4_G s.u. on c - Axis Small or Missing .............    0.00014 Ang.
PLAT912_ALERT_4_G Missing # of FCF Reflections Above STh/L=  0.600        17 Note
PLAT978_ALERT_2_G Number C-C Bonds with Positive Residual Density.        16 Info
```

0 **ALERT level A** = Most likely a serious problem - resolve or explain
0 **ALERT level B** = A potentially serious problem, consider carefully
0 **ALERT level C** = Check. Ensure it is not caused by an omission or oversight
5 **ALERT level G** = General information/check it is not something unexpected

0 ALERT type 1 CIF construction/syntax error, inconsistent or missing data
1 ALERT type 2 Indicator that the structure model may be wrong or deficient
0 ALERT type 3 Indicator that the structure quality may be low
3 ALERT type 4 Improvement, methodology, query or suggestion
1 ALERT type 5 Informative message, check

It is advisable to attempt to resolve as many as possible of the alerts in all categories. Often the minor alerts point to easily fixed oversights, errors and omissions in your CIF or refinement strategy, so attention to these fine details can be worthwhile. In order to resolve some of the more serious problems it may be necessary to carry out additional measurements or structure refinements. However, the purpose of your study may justify the reported deviations and the more serious of these should normally be commented upon in the discussion or experimental section of a paper or in the "special_details" fields of the CIF. checkCIF was carefully designed to identify outliers and unusual parameters, but every test has its limitations and alerts that are not important in a particular case may appear. Conversely, the absence of alerts does not guarantee there are no aspects of the results needing attention. It is up to the individual to critically assess their own results and, if necessary, seek expert advice.

Publication of your CIF in IUCr journals

A basic structural check has been run on your CIF. These basic checks will be run on all CIFs submitted for publication in IUCr journals (*Acta Crystallographica*, *Journal of Applied Crystallography*, *Journal of Synchrotron Radiation*); however, if you intend to submit to *Acta Crystallographica Section C* or *E* or *IUCrData*, you should make sure that full publication checks are run on the final version of your CIF prior to submission.

Publication of your CIF in other journals

Please refer to the *Notes for Authors* of the relevant journal for any special instructions relating to CIF submission.

PLATON version of 29/11/2023; check.def file version of 14/09/2023

Datablock 20231215_c73-1 - ellipsoid plot

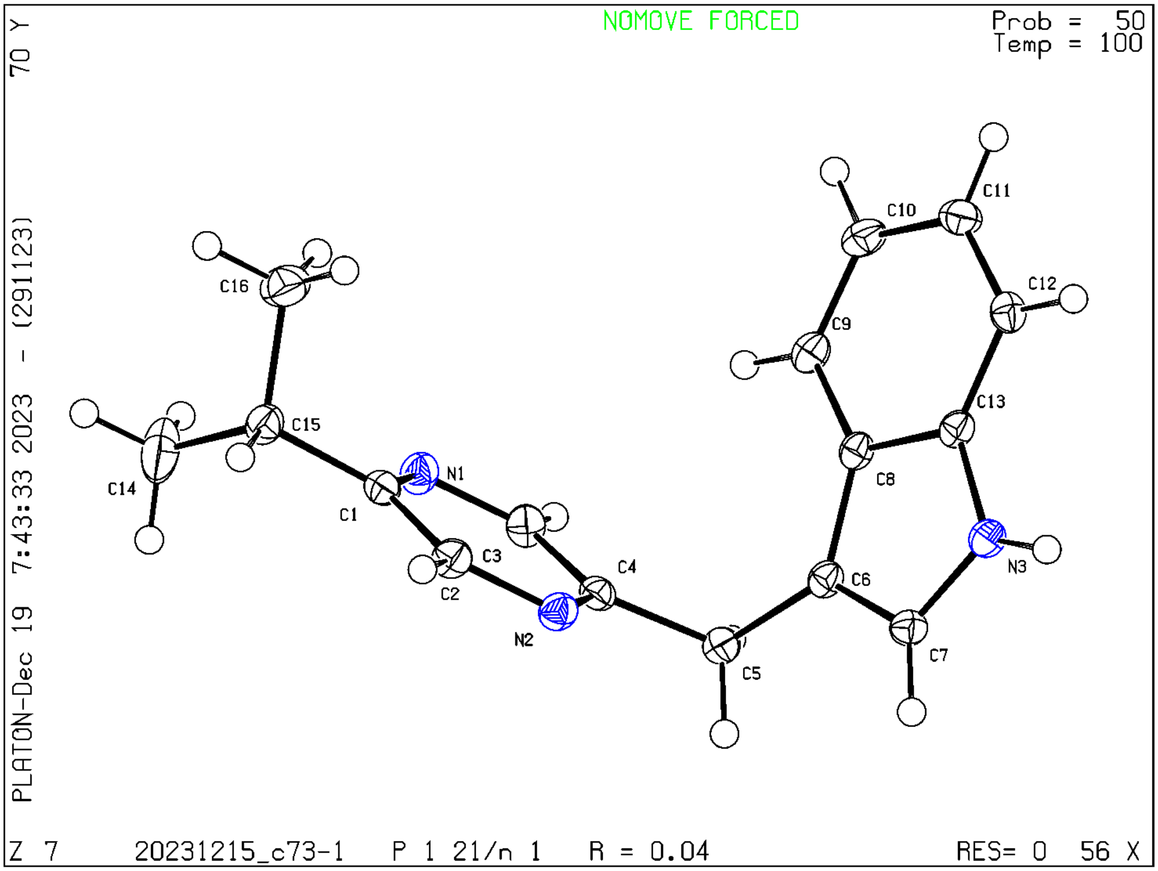

Supplement: Supplementary file 3 — Additional file 3. [file 13659_2026_601_MOESM3_ESM.pdf]
