# Supplementary material for: Discovery of unprecedented prenylated indole piperazines and pyrazines through cryptic biosynthetic gene cluster heterologous expression
Source: Nat Prod Bioprospect. 2026 Apr 3;16(1):49. doi: 10.1007/s13659-026-00601-7 (PMC13047030; doi:10.1007/s13659-026-00601-7)

checkCIF/PLATON report

You have not supplied any structure factors. As a result the full set of tests cannot be run.

THIS REPORT IS FOR GUIDANCE ONLY. IF USED AS PART OF A REVIEW PROCEDURE FOR PUBLICATION, IT SHOULD NOT REPLACE THE EXPERTISE OF AN EXPERIENCED CRYSTALLOGRAPHIC REFEREE.

No syntax errors found. CIF dictionary Interpreting this report

Datablock: Ag-220-253

```
Bond precision:   C-C = 0.0027 A          Wavelength=1.54184

Cell:            a=15.2897(13)    b=5.4351(4)        c=17.7181(16)
                 alpha=90         beta=94.308(8)     gamma=90
Temperature:     150 K

                Calculated                  Reported
Volume          1468.2(2)                   1468.2(2)
Space group     P 21/c                      P 1 21/c 1
Hall group      -P 2ybc                     -P 2ybc
Moiety formula  C16 H17 N2 O, C H O2        C16 H17 N2 O, C H O2
Sum formula     C17 H18 N2 O3               C17 H18 N2 O3
Mr              298.33                      298.34
Dx,g cm-3       1.350                       1.350
Z               4                           4
Mu (mm-1)       0.762                       0.762
F000            632.0                       634.1
F000'           633.96
h,k,lmax        19,6,22                     18,6,21
Nref            2959                        2865
Tmin,Tmax       0.865,0.899                 0.567,1.000
Tmin'           0.865

Correction method= # Reported T Limits: Tmin=0.567 Tmax=1.000
AbsCorr = MULTI-SCAN
```

Data completeness= 0.968 Theta(max)= 73.630

R(reflections)= 0.0580(2473) wR2(reflections)= 0.1727(2865)

S = 1.053 Npar= 201

The following ALERTS were generated. Each ALERT has the format
test-name_ALERT_alert-type_alert-level.
Click on the hyperlinks for more details of the test.

Alert level C

```
PLAT911_ALERT_3_C Missing FCF Refl Between Thmin & STh/L=    0.600          5 Report
                7  6  0,   7  6  1,   6  3  2,  -7  1  9,   3  4 16,
```

Alert level G

```
PLAT068_ALERT_1_G Reported F000 Differs from Calcd (or Missing)...     Please Check
PLAT072_ALERT_2_G SHELXL First  Parameter in WGHT  Unusually Large       0.10 Report
PLAT073_ALERT_1_G H-atoms ref., but hydrogen treatment Reported as     constr Check
PLAT720_ALERT_4_G Number of Unusual/Non-Standard Labels ..........         40 Note
              O001    O002    O003    N004    N005    C006    C007    C008
              C009    C00A    C00B    C00C    C00D    C00E    C00F    C00G
              C00H    C00I    C00J    C00K    C00L    C00M    H00I    H00K
              H00J    H00C    H00F    H009    H00E    H00G    H00N    H00A
              H00B    H00D    H00H    H00L    H00M    H001    H004    H005
PLAT769_ALERT_4_G CIF Embedded Explicitly Supplied Scattering Data          4 Note
PLAT912_ALERT_4_G Missing # of FCF Reflections Above STh/L=  0.600         86 Note
PLAT948_ALERT_5_G Externally Supplied Scattering Factors       CIF          4 Note
PLAT960_ALERT_3_G Number of Intensities with I < - 2*Sigma(I) ....          3 Check
PLAT969_ALERT_5_G The 'Henn et al.' R-Factor-gap value ..........       2.601 Note
              Predicted wR2: Based on SigI**2  6.64 or SHELX Weight 16.40
PLAT978_ALERT_2_G Number C-C Bonds with Positive Residual Density.          3 Info
PLAT982_ALERT_1_G The  C-f'=    0.0192 Deviates from  IT-Value =       0.0181 Check
PLAT982_ALERT_1_G The  N-f'=    0.0326 Deviates from  IT-Value =       0.0311 Check
PLAT982_ALERT_1_G The  O-f'=    0.0524 Deviates from  IT-Value =       0.0492 Check
PLAT983_ALERT_1_G The  O-f"=    0.0338 Deviates from  IT-Value =       0.0322 Check
```

---

0 **ALERT level A** = Most likely a serious problem - resolve or explain
0 **ALERT level B** = A potentially serious problem, consider carefully
1 **ALERT level C** = Check. Ensure it is not caused by an omission or oversight
14 **ALERT level G** = General information/check it is not something unexpected

6 ALERT type 1 CIF construction/syntax error, inconsistent or missing data
2 ALERT type 2 Indicator that the structure model may be wrong or deficient
2 ALERT type 3 Indicator that the structure quality may be low
3 ALERT type 4 Improvement, methodology, query or suggestion
2 ALERT type 5 Informative message, check

Validation response form

Please find below a validation response form (VRF) that can be filled in and pasted into your CIF.

```
# start Validation Reply Form
_vrf_PLAT911_Ag-220-253
;
PROBLEM: Missing FCF Refl Between Thmin & STh/L=    0.600          5 Report
RESPONSE: ...
;
# end Validation Reply Form
```

It is advisable to attempt to resolve as many as possible of the alerts in all categories. Often the minor alerts point to easily fixed oversights, errors and omissions in your CIF or refinement strategy, so attention to these fine details can be worthwhile. It is up to the individual to critically assess their own results and, if necessary, seek expert advice.

PLATON version of 26/09/2025; check.def file version of 20/09/2025

Datablock Ag-220-253 - ellipsoid plot

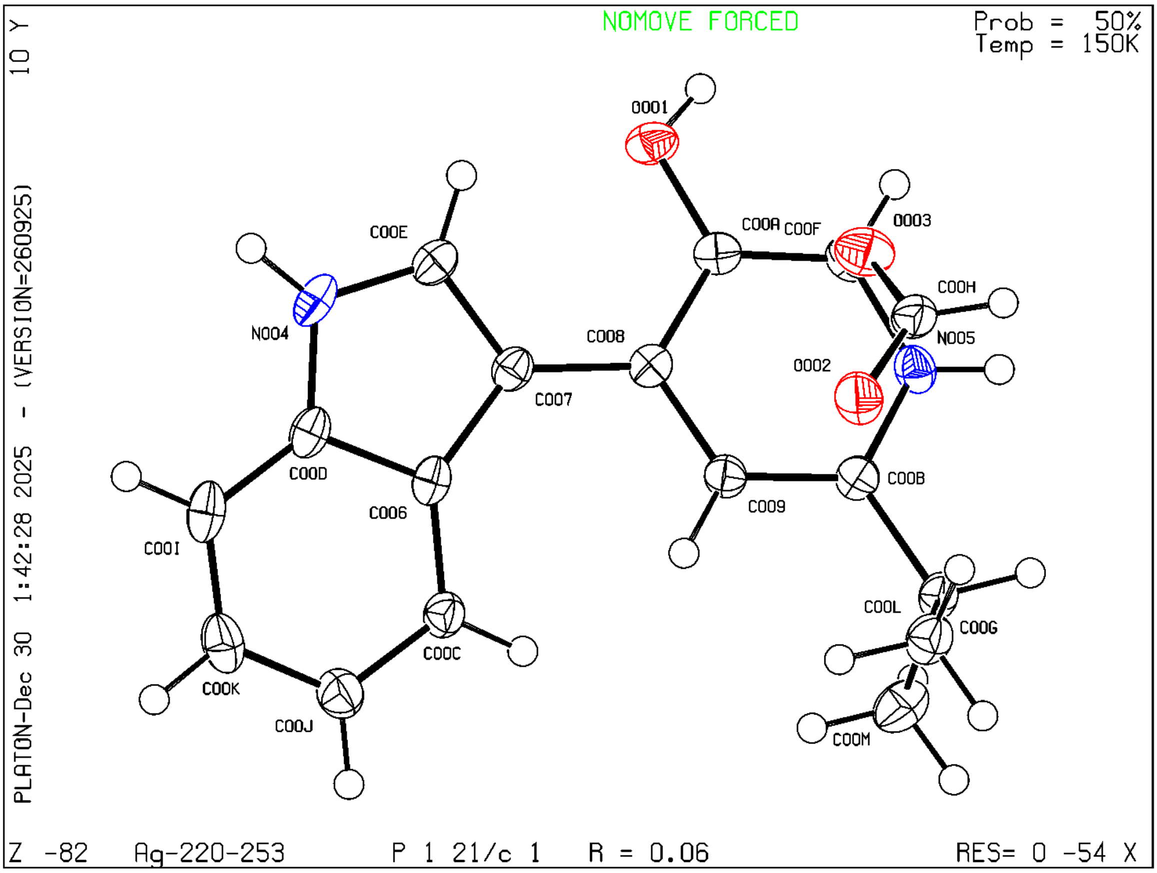

Supplement: Supplementary file 5 — Additional file 5. [file 13659_2026_601_MOESM5_ESM.pdf]
